# The Computational Preventive Potential of the Rare Flavonoid, Patuletin, Isolated from *Tagetes patula*, against SARS-CoV-2

**DOI:** 10.3390/plants11141886

**Published:** 2022-07-20

**Authors:** Ahmed M. Metwaly, Eslam B. Elkaeed, Bshra A. Alsfouk, Abdulrahman M. Saleh, Ahmad E. Mostafa, Ibrahim H. Eissa

**Affiliations:** 1 Pharmacognosy and Medicinal Plants Department, Faculty of Pharmacy (Boys), Al-Azhar University, Cairo 11884, Egypt; aemostafa@azhar.edu.eg; 2 Biopharmaceutical Products Research Department, Genetic Engineering and Biotechnology Research Institute, City of Scientific Research and Technological Applications (SRTA-City), Alexandria 21934, Egypt; 3 Department of Pharmaceutical Sciences, College of Pharmacy, AlMaarefa University, Riyadh 13713, Saudi Arabia; ikaeed@mcst.edu.sa; 4 Department of Pharmaceutical Sciences, College of Pharmacy, Princess Nourah bint Abdulrahman University, P.O. Box 84428, Riyadh 11671, Saudi Arabia; baalsfouk@pnu.edu.sa; 5 Pharmaceutical Medicinal Chemistry & Drug Design Department, Faculty of Pharmacy (Boys), Al-Azhar University, Cairo 11884, Egypt; abdo.saleh240@azhar.edu.eg

**Keywords:** *Tagetes patula*, SARS-CoV-2 RNA-dependent RNA polymerase, patuletin, molecular similarity, 3D-Flexible alignment, molecular docking, ADMET, toxicity, MD simulations

## Abstract

The rare flavonoid, patuletin, was isolated from the flowers of *Tagetes patula* growing in Egypt. The rarity of the isolated compound inspired us to scrutinize its preventive effect against COVID-19 utilizing a multi-step computational approach. Firstly, a structural similarity study was carried out against nine ligands of nine SARS-CoV-2 proteins. The results showed a large structural similarity between patuletin and **F86**, the ligand of SARS-CoV-2 RNA-dependent RNA polymerase (RdRp). Then, a 3D-Flexible alignment study of patuletin and **F86** verified the proposed similarity. To determine the binding opportunity, patuletin was docked against the RdRp showing a correct binding inside its active pocket with an energy of −20 kcal/mol that was comparable to that of **F86** (−23 kcal/mol). Following, several MD simulations as well as MM-PBSA studies authenticated the accurate binding of patuletin in the RdRp via the correct dynamic and energetic behaviors over 100 ns. Additionally, in silico ADMET studies showed the general safety and drug-likeness of patuletin.

## 1. Introduction

Since the oldest historical records, nature granted humans their primary needs including treatments, food, as well as cosmetical products [1,2]. Modern science relates the biological activities of natural products to the presence of various sorts of secondary metabolites such as hydrocarbons [3,4,5], isochromenes [6], α-pyrones [7,8], diterpenes [9], sesquiterpenes [10,11], steroids [12,13], and saponins [14,15,16]. The computational (computer-based or in silico) chemistry approaches are efficient tools that have been employed to examine the biological activities of compounds virtually. These approaches have been effectively utilized in drug design and drug discovery. The computational chemistry methods were employed to determine the biological activities of natural, synthesized, and semi-synthesized compounds. The huge advancement that occurred in software in the last decade enabled researchers to apply the structure–activity relationship principles to precisely predict the biological activity of a new compound based on its physical and chemical properties. Our team employed computer-based chemistry strategies to disclose the potential inhibitive effects of the several secondary metabolites against SARS-CoV-2 that have been isolated from *Asteriscus* sp. [17], *Monanchora* sp. [18], and *Artemisia* spp. [19,20,21]. Additionally, we presented a well-designed in silico approach to select the most relevant inhibitor compound among a huge set of compounds and we applied that method to introduce several opportune anti-COVID-19 compounds from 69 isoflavonoids [22], semi-synthetic compounds [23], 310 natural antivirals [24,25], and 3009 FDA-approved compounds [26,27].

Patuletin is a rare flavonol that has been isolated for the first time from *Tagetes patula* in 1941 [28]; then, it was isolated a few times from other plant species such as *Eriocaulon* sp. [29] and *Urtica urens* [30]. Later, patuletin was employed as a chemotaxonomic marker for *Tagetes patula* [31]. Despite the rarity of patuletin, it exhibited several promising biological activities such as anti-inflammatory [32,33], cytotoxic [34,35], antimicrobial [36], and neuroprotective [37].

Here in this study, we report the isolation of the rare flavonol, patuletin, from the flowers of *Tagetes patula*. Due to being a rare flavonol, its potential effect as a treatment for COVID-19 was examined. The start point of our work was the chemical structures of diverse ligands of different SARS-CoV-2 proteins. Our study indicated the great structural similarity of patuletin and **F86**, the co-crystallized ligand of RdRp (PDB ID: 7BV2), expecting an efficient binding to patuletin in the active site of RdRp. This correct binding was confirmed by applying molecular docking as well as MD simulations and MM-PBSA.

## 2. Results

### 2.1. Isolation and Characterization

**Figure 1:**
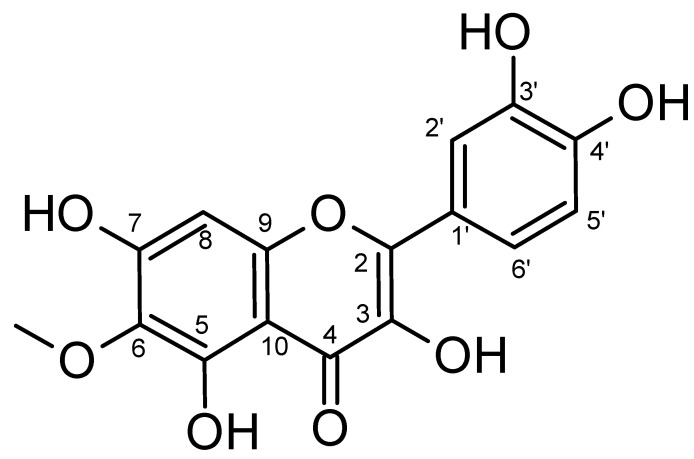
Patuletin’s chemical structure.

A total of 2 kg of *Tagetes patula* L. flowers were extracted with 70% ethanol three times to afford 210 gm of total extract. The extract was suspended in water and fractionated against hexane, CH_2_Cl_2_, and n-butanol. Then, the butanol fraction was subjected to a silica gel column to provide 8 different fractions. Fraction 3 was further purified with Sephadex LH-20 to furnish 110 mg of patuletin (Figure 1). The ^1^H NMR spectrum of patuletin showed one singlet aromatic signal at δH 6.54 ppm for H-8 in addition to three other multiplied aromatic signals resonating at 7.70 ppm d (*J* = 2 Hz, H-2′), 6.92 ppm d (*J* = 8 Hz, H-5′), and 7.56 ppm dd (*J* = 2 Hz, *J* = 8 Hz, H-6′). Furthermore, a signal of a methoxy group was detected at δH 3.79 ppm (s). Additionally, the distinctive chelated proton signal of the OH of C-5 resonated as a sharp singlet at δH 12.62 ppm (because of the formation of an intramolecular hydrogen bond (H-B) with the carbonyl group) (see Table 1). The ^13^C spectral data indicated the existence of 15 carbon atoms in addition to a methoxy group. The obtained data was completely consistent with the previously published spectral data of patuletin [38]. 

### 2.2. Molecular Similarity 

**Figure 2:**
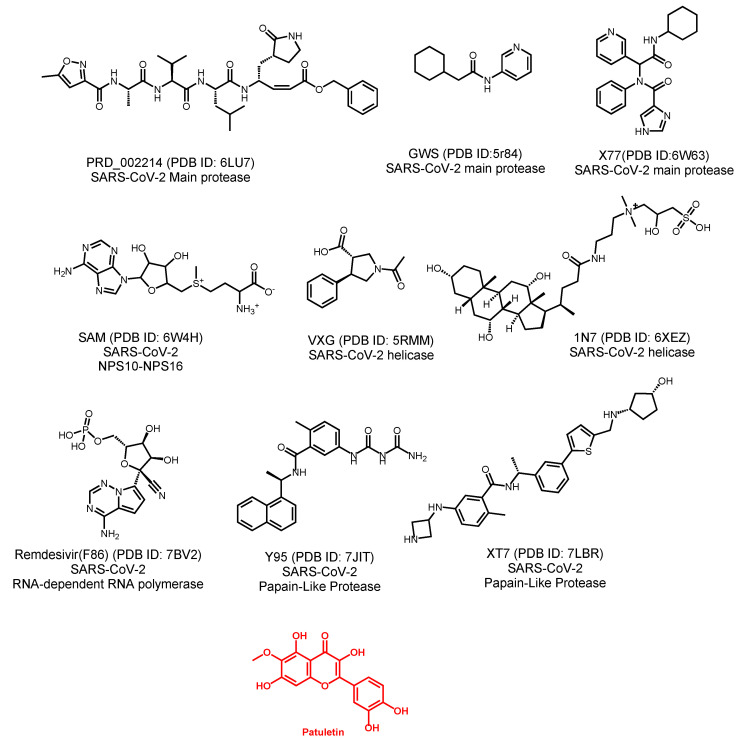
The co-crystallized ligands of SARS-CoV-2 proteins and patuletin.

Our key point in this investigation is the co-crystallized ligand. The co-crystallized ligand is a molecule that can bind efficiently with a particular protein and crystallize it [39]. The structure–activity relationship rules indicate that any two compounds that have a resemblance in chemical structures, are expected to show similar biological activities through binding with the same receptor [40]. The molecular similarity study describes and compares the whole structures of the reference compound as well as the examined compound, using descriptors such as steric, topological, electronic, and/or physical characteristics [41]. Accordingly, a molecular similarity study was conducted to compare the chemical structure of patuletin with those of nine co-crystallized ligands of vital proteins of SARS-CoV-2 (Figure 2). Our aim is to investigate the structural similarity that may be associated with the binding affinity. Accordingly, we utilized a 2D molecular similarity assay to examine the similarity.

**Figure 3:**
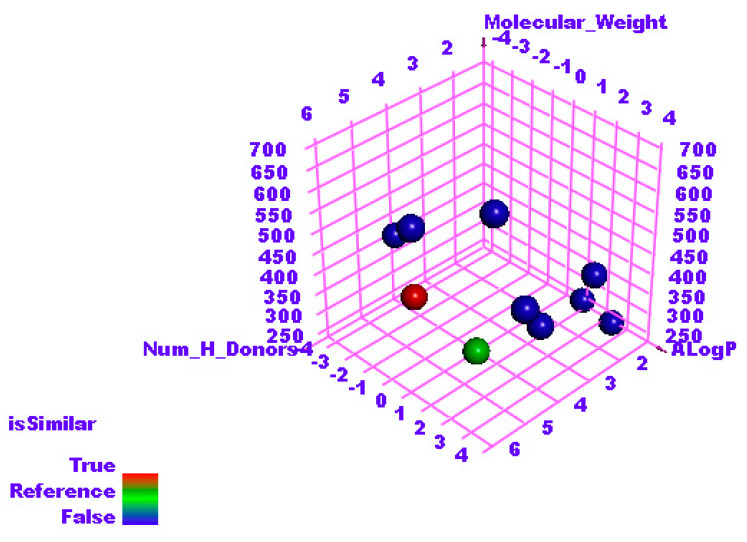
The results of similarity analysis of the considered ligands and patuletin.

The structural similarity between patuletin and the considered ligands was checked by applying the software, Discovery studio. The subsequent structural characteristics were investigated and compared in patuletin and the examined ligands; molecular weight (M-W) [42], partition coefficient (ALog p) [43], H-B donors (H.B-D) [44], H-B acceptors (H.B-A) [45], molecular fractional polar surface area (MFP-SA) [46], number of rotatable bonds (N-RB) [47], number of rings (N-R) and aromatic rings (N-AR) [48]. The outputs indicated the existence of a great degree of structural similarity between patuletin and the co-crystallized ligand **F86**, of RdRp, (PDB ID: 7BV2) (Table 2 and Figure 3).

### 2.3. Flexible Alignment 

**Figure 4:**
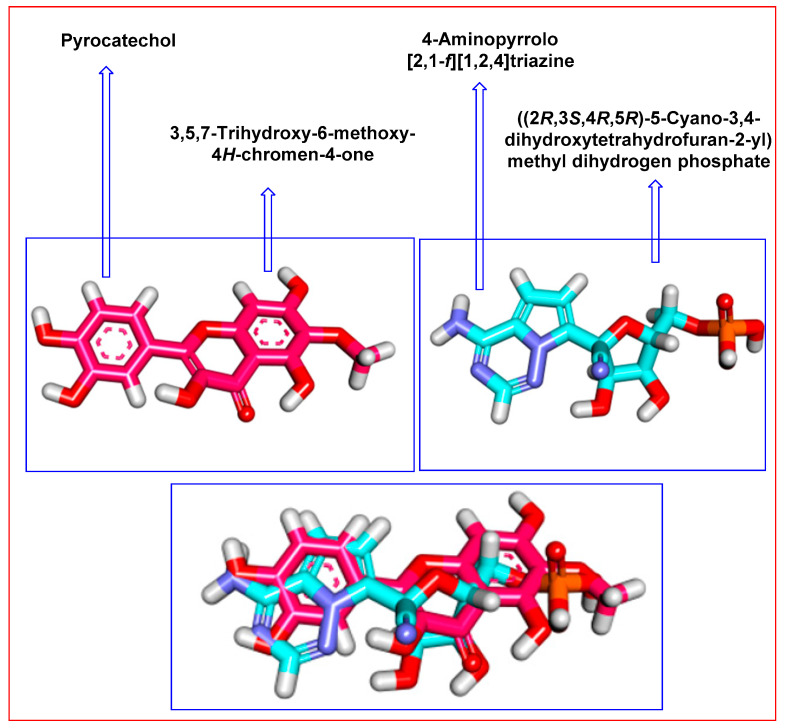
Flexible alignment of patuletin (**pink**) with **F86** (**turquoise**).

To substantiate the obtained results, a 3D-Flexible alignment of patuletin with **F86** was directed. The result revealed the general good overlapping. Interestingly, as shown in Figure 4, patuletin showed the same spatial orientation as **F86**. In detail, the pyrocatechol moiety of patuletin showed the same orientation as the 4-aminopyrrolo [2,1-*f*][1,2,4] triazine moiety of **F86**. Additionally, the 3,5,7-Trihydroxy-6-methoxy-4H-chromen-4-one moiety of patuletin exhibited close orientation to the ((2*R*,3*S*,4*R*,5*R*)-5-cyano-3,4-dihydroxytetrahydrofuran-2-yl)methyl dihydrogen phosphate moiety of **F86**.

### 2.4. Docking Studies 

To investigate the binding interactions of patuletin with the RdRp’s active pocket, docking studies were performed using **F86** as a reference. The binding free energy (∆G) between patuletin and RdRp’s active pocket, besides to the correct binding mode were the factors of evaluation. 

**Figure 5:**
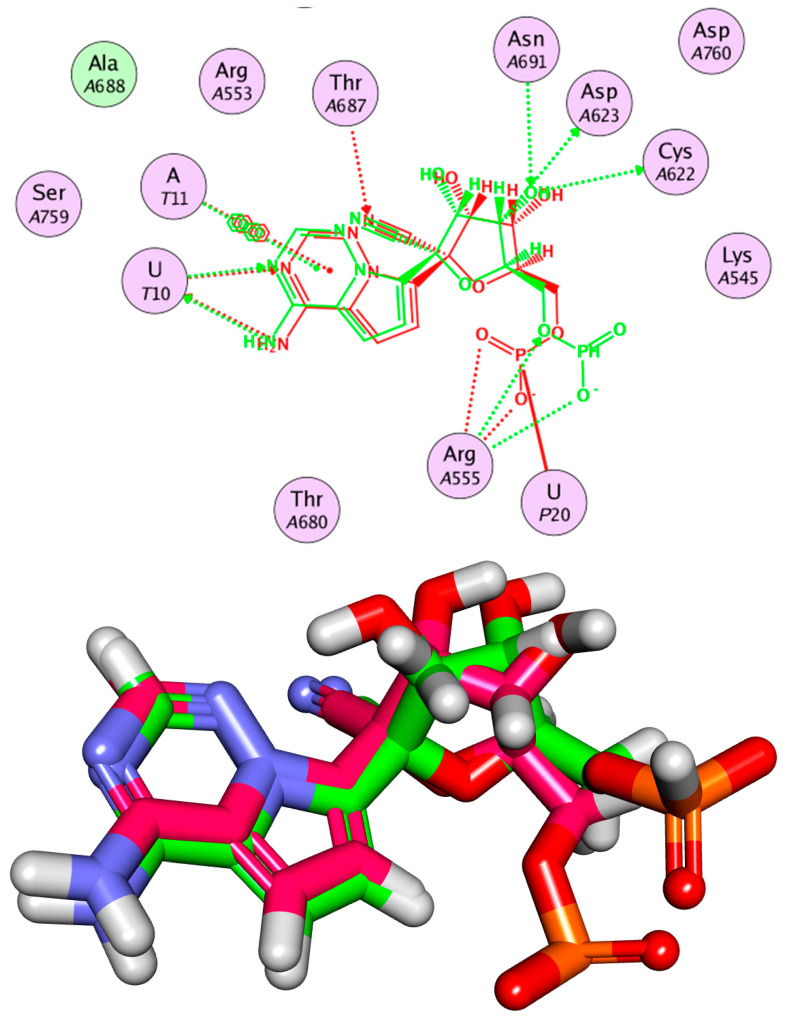
Superimposition of docked **F86** (**green**) and the original one (**pink**) in RdRp’s active pocket.

At first, verification of the docking process was carried out through the re-docking procedure for **F86** against the active pocket of RdRp. The the validity of the docking process was confirmed as the obtained RMSD value between the generated pose and the original one was 1.61 °A (Figure 5).

**Figure 6:**
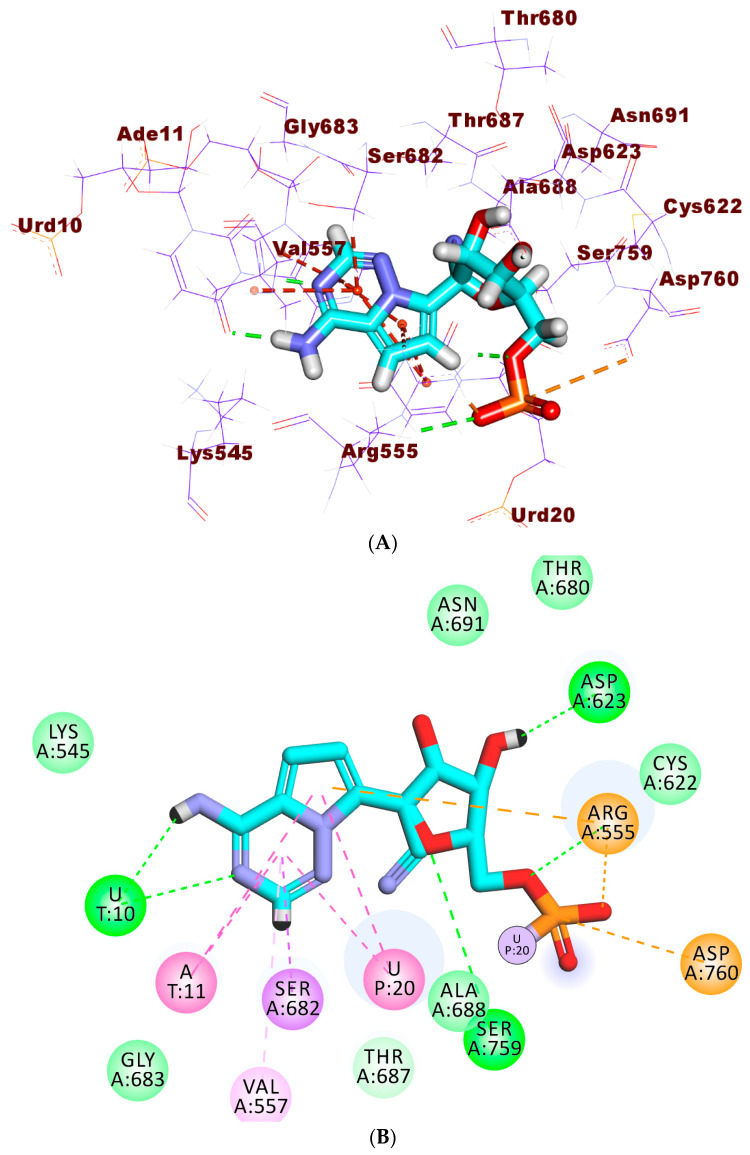

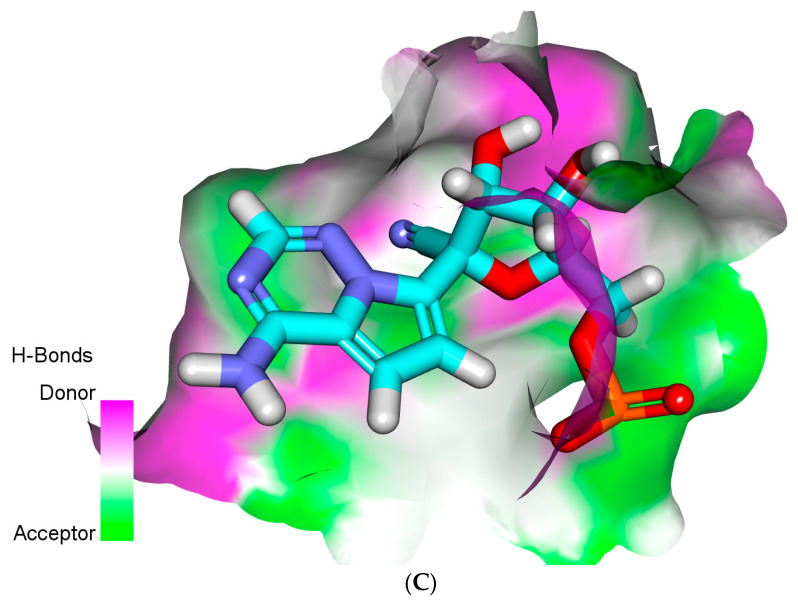
(**A**) The 3D, (**B**,**C**) surface mapping of **F86** in RdRp’s active site.

Regarding **F86**, it exhibited a binding free energy value of −23.71 kcal/mol. Compound **F86** exhibited five H-Bs, six hydrophobic interactions (H-I), and three electrostatic interactions (E-I). The 4-aminopyrrolo [2,1-*f*][1,2,4] triazine moiety oriented to the 1st pocket of the active site forming two H-Bs with Urd10. In addition, it formed six H-I with Urd20, Ade11, Arg555, and Val557. Additionally, it formed an electrostatic attraction with Arg555. The sugar moiety formed two H-Bs with Ser757 and Asp623. The phosphate derivative moiety formed one H-B with Arg555, and two E-Is with Asp760 and Arg555 (Figure 6).

**Figure 7:**
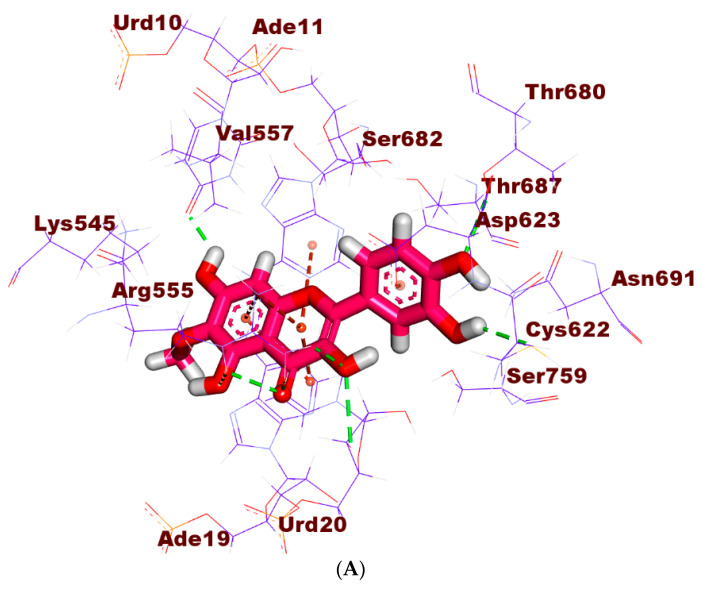

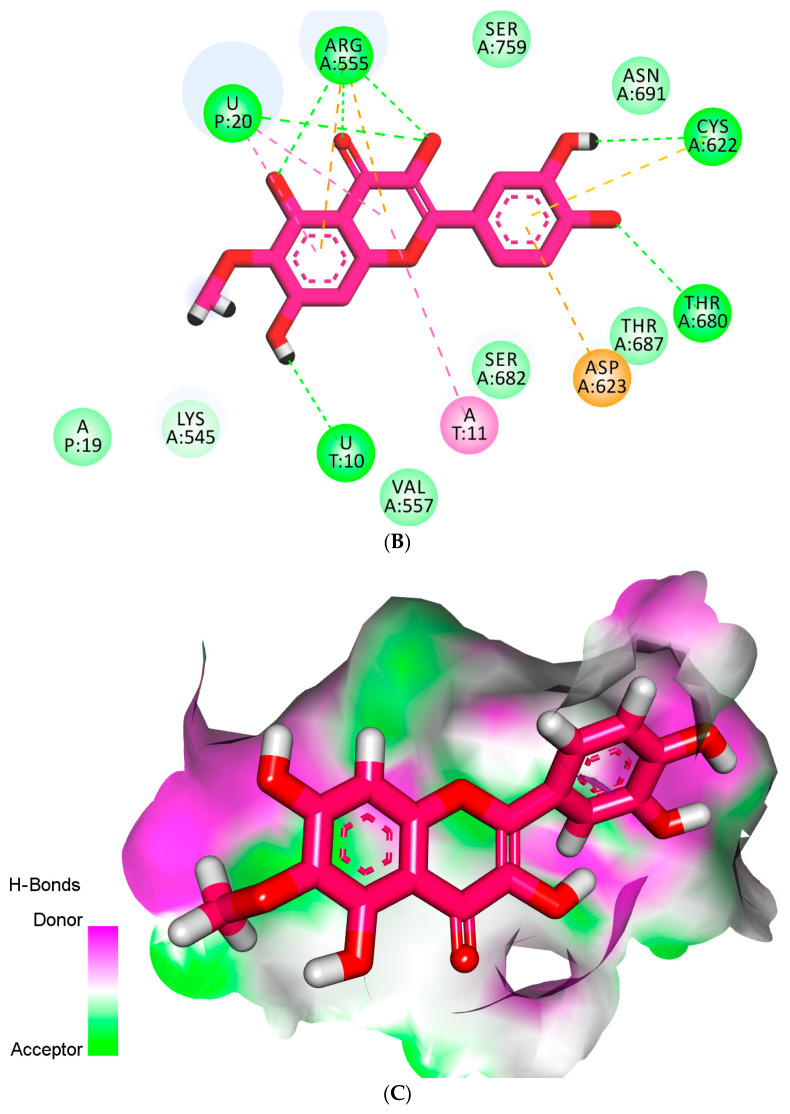
(**A**) The 3D, (**B**) 2D, and (**C**) surface mapping of patuletin in RdRp’s active site.

The binding mode of patuletin showed a binding free energy value of −20.30 kcal/mol. The pyrocatechol moiety was oriented into the first pocket of the receptor to form two H-Bs with Cys622 and Thr680. In addition, it was incorporated in two E-Is with Cys622 and Asp623. Furthermore, the 3,5,7-Trihydroxy-6-methoxy-4H-chromen-4-one moiety formed five H-Bs with Urd20, Urd10, and Arg555. In addition, it formed three H-I with Urd20 and Ade11. Additionally, it formed two electrostatic attractions with Arg555 (Figure 7).

### 2.5. In Silico ADMET Analysis

In order to prevent late drug withdrawals, the analysis of the ADMET properties of any new compound should be conducted early in drug discovery. Despite the fact that various in vitro studies can investigate ADMET properties, in silico studies are still more advantageous given the limitations of cost, time, effort, and strict regulations regarding animal lives [49]. The ADMET profile of patuletin was determined using discovery studio against remdesivir, **F86**, as a reference.

**Figure 8:**
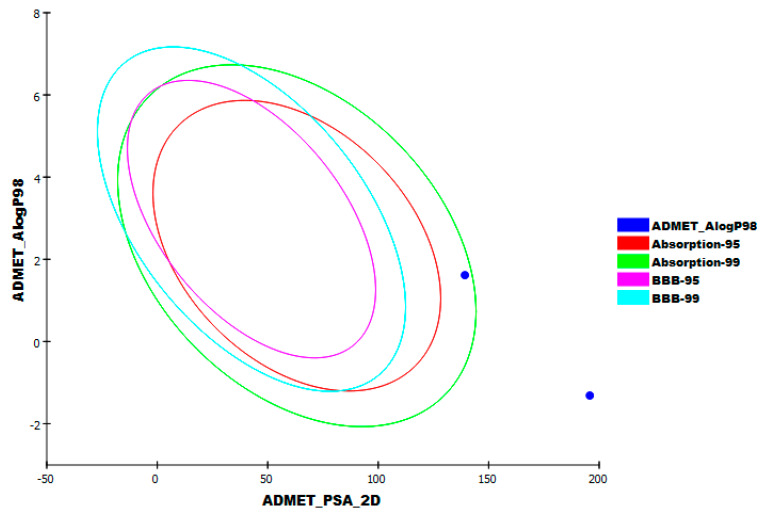
ADMET study of patuletin and remdesivir.

As Figure 8 illustrates, patuletin displayed a very low potential to penetrate the BBB. Patuletin presented a good aqueous solubility as well as moderate intestinal absorption levels. The ability of patuletin to inhibit the cytochrome P450, CYP2D6, and to bind to the plasma protein were predicted as non-inhibitory and less than 90%, respectively. The results of remdesivir were similar to those of patuletin except for the poor absorption level.

### 2.6. In Silico Toxicity Studies

The in silico approach has had an essential contribution in toxicity, prediction through drug development in order to avoid ethical regulations, resource availability, as well as time-wasting in usual in vitro and in vivo studies [50]. The purpose of in silico toxicity prediction is to predict toxicity using the structure–activity relationship (SAR) through comparing basic chemical structural properties of the molecules with the structures of thousands of compounds of known safety and toxicity [51].

Seven models of toxicity were predicted to patuletin using discovery studio against remdesivir, **F86**, as a reference (Table 3). The examined models are: Ames prediction (A-C), carcinogenic potency in rats (R-TD_50_), rat maximum tolerated dose (R-MTD), Rat Oral LD_50_ (R- LD_50_), chronic LOAEL in rats (R- LOAEL), eye, ocular, irritation model (O-Ir), and skin irritation model (S-Ir).

### 2.7. MD Simulations

**Figure 9:**
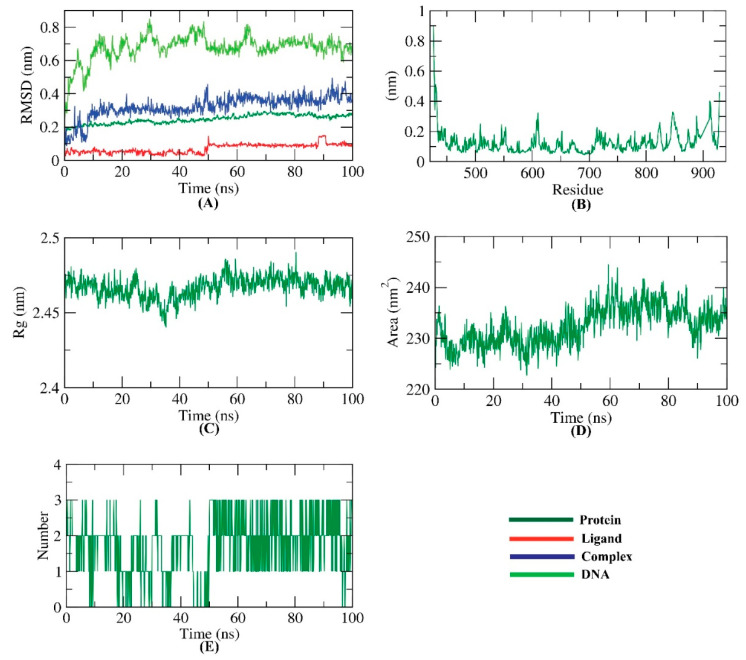
MD’s results; (**A**) RMSD values of the patuletin–RdRp complex, (**B**) RMSF of the patuletin–RdRp complex, (**C**) Rg of the patuletin–RdRp complex, (**D**) SASA of the patuletin–RdRp complex, (**E**) H-bonding of the patuletin–RdRp complex.

A molecular docking study is an in silico study that can reveal a ligand’s exact location inside a protein based on its structure. However, docking studies have the disfavor that they describe the interaction of proteins as a rigid (fixed) unit disregarding the conformational changes in the protein and ligand structures after binding [52]. Contradictory, the MD simulations experiments can provide a thorough understanding of how proteins behave at a cellular and atomic level as well as how their structure changes over time [53]. Accordingly, MD simulations can be used to describe exactly ligands’ effects on protein conformation from both dynamic and energy perspectives [54]. As a result of the interaction of a compound inside a protein’s active site, structural changes have occurred [55]. The RdRp’s active site is a complex of active polymerase protein (composed of amino acids) and nucleotides triphosphate [56]. The obtained conformational changes have been explored as RMSD for RdRp (protein and nucleotides), patuletin, and the patuletin–RdRp complex in order to evaluate the stability of the patuletin–RdRp complex after binding. Intriguingly, low RMSD values were recorded with no major fluctuations in the patuletin–RdRp complex as well as its single components (Figure 9A).

The flexibility of the patuletin–RdRp complex was examined in terms of RMSF to predict the degree of fluctuation of RdRp in the MD simulation experiment. Stimulatingly, the binding of patuletin did not cause significant changes in the RdRp flexibility (Figure 9B).

The radius of gyration, R_g_, which describes the RMSD of a weighted mass unit of RdRp’s atoms from their mass center, provides accurate information about the 3D changes in the enzyme alongside its compactness. The degree of fluctuation, R_g_ value, during simulation time is inversely proportional to compactness and stability. Captivatingly, the patuletin–RdRp complex R_g_ was found to be less than the starting time (Figure 9C) indicating a good degree of stability.

The interaction of the patuletin–RdRp complex with the circumferential solvents was also computed by SASA during the simulation time. Engagingly, the SASA values of the patuletin–RdRp complex were lower than the starting period (Figure 9D), which implies a reduction in the surface area and, subsequently, higher stability.

It is clear that H-bonding is a critical factor in stabilizing the patuletin–RdRp complex, so MD simulation experiments were conducted to indicate that the highest number of conformations of the complex formed three H-Bs (Figure 9E).

**Figure 10:**
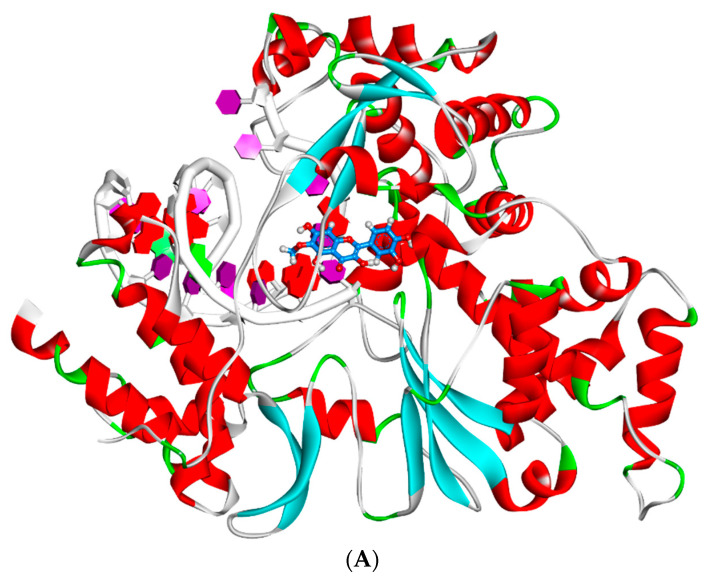

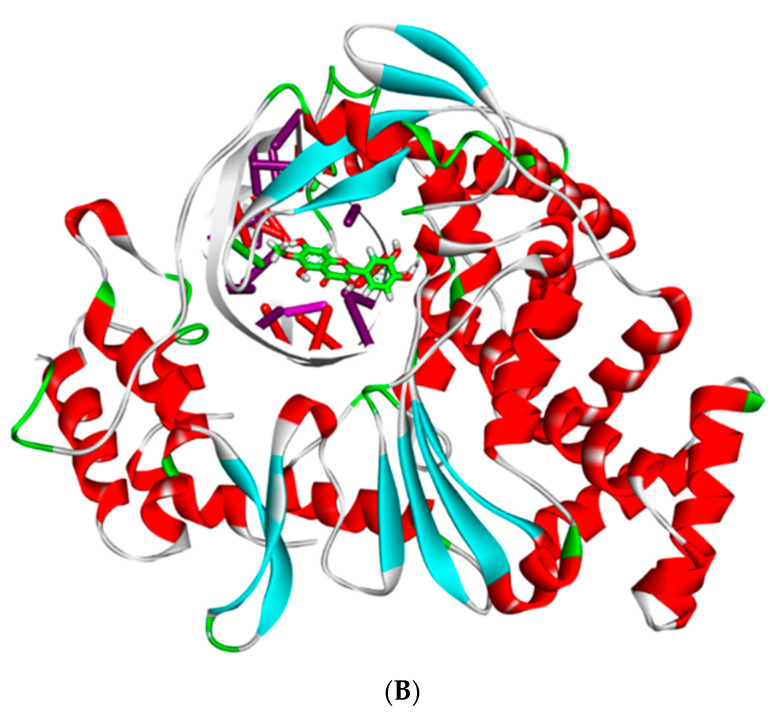
Conformational structures for the patuletin and RdRp at the first (**A**) and 100th (**B**) nanoseconds of the MD run.

The conformational changes that occurred because of the binding of patuletin to RdRp were examined during the first and 100^th^ nanoseconds of the MD run as explained in Figure 10. It was confirmed that conformational changes have occurred in the patuletin–RdRp complex, as well as the binding stability and integrity of the patuletin–RdRp complex were indicated as patuletin was bonded perfectly to the RdRp’s active pocket through the 100 ns of the run.

### 2.8. MM-PBSA

**Figure 11:**
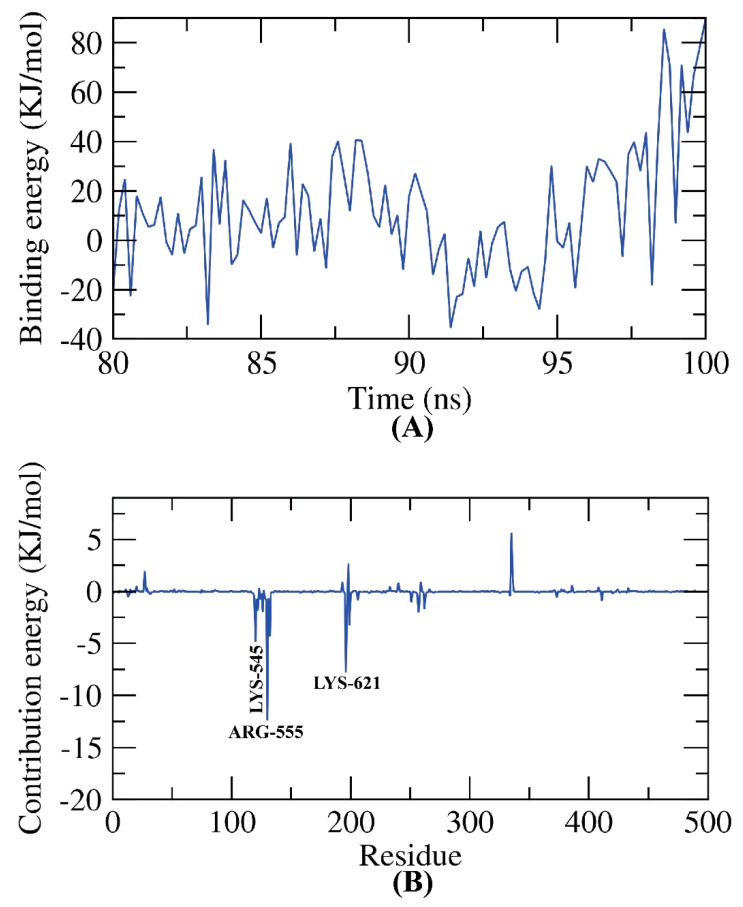
MM-PBSA patuletin–amino acids of RdRp.

As we mentioned, the RdRp’s active site is a complex of active polymerase protein and nucleotides triphosphate [56]. The average free binding energy of both types of bindings (patuletin–amino acids and patuletin–nucleotides) was based on MD trajectories from the last stable 20 ns of MD production run at a time interval of 100 ps. Figure 11A presents the average free binding energy of patuletin–amino acids of RdRp showing a very low binding free energy of −25 KJ/mol (−6 kcal/mol). Additionally, the binding energy remained stable throughout the examination run time indicating the accurate binding of the complex.

Next, the total binding free energy of the patuletin–amino acids of RdRp was analyzed in order to establish which of the amino acid residues participated most in the binding with patuletin. Three amino acids (Figure 11B) of the polymerase residues contributed more than −5 KJ/mol (−1.2 kcal/mol) regarding the binding energy and were considered essential (vital) residues.

**Figure 12:**
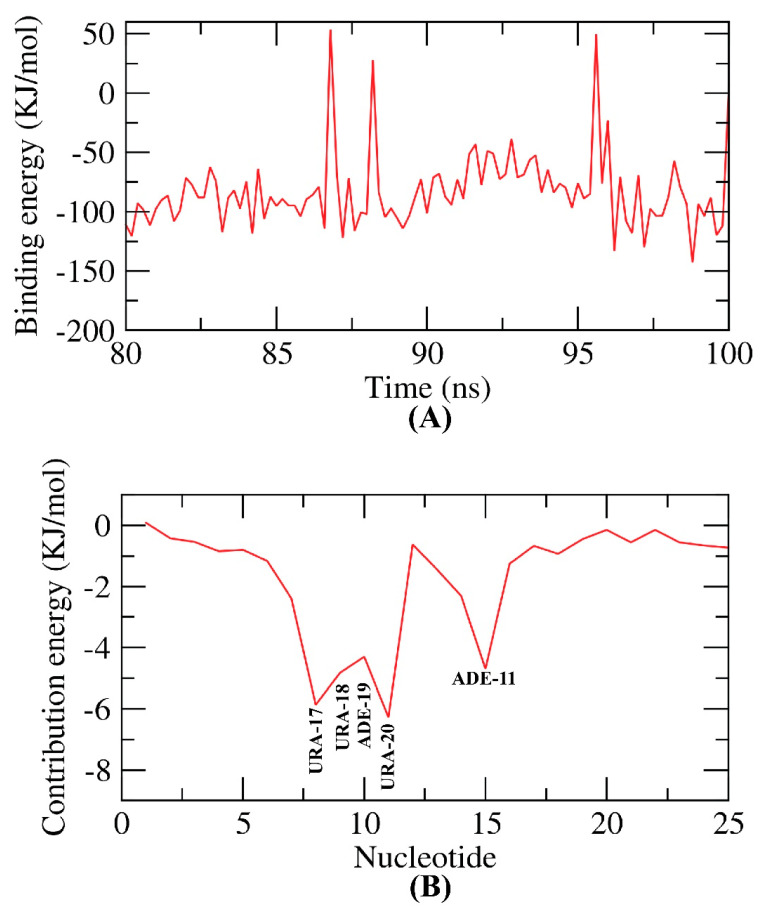
MM-PBSA patuletin–nucleotides of RdRp.

The average free binding energy of patuletin–nucleotides is illustrated in Figure 12A. Interestingly, the average free binding energy of patuletin–nucleotides of RdRp showed a very low binding free energy of −120 KJ/mol (−28.7 kcal/mol). Additionally, the binding energy was stable among all the examination run times showing the precise binding of the complex.

Next, the total binding free energy of the patuletin–nucleotides of RdRp was analyzed in order to establish which of the nucleotides participated most in the binding with patuletin. Five nucleotides (Figure 12B) of the RdRp contributed more than −5 KJ/mol (−1.2 kcal/mol) regarding the binding energy and were considered vital nucleotides.

## 3. Materials and Methods

### 3.1. Isolation of Patuletin

Extraction, isolation, and identification of patuletin were addressed scrupulously in the supporting data (Supplementary Materials).

### 3.2. Molecular Similarity

Molecular similarity of patuletin was accomplished using Discovery Studio 4.0 [24,57] and was addressed scrupulously in the supporting data.

### 3.3. Docking Studies

Docking of patuletin against RdRp was accomplished using MOE2014 and outputted files were visualized using Discovery Studio 4.0 software [58,59,60] and were addressed scrupulously in the Supporting Data.

### 3.4. ADMET

ADMET patuletin was accomplished using Discovery Studio 4.0 [61,62] and was addressed scrupulously in the Supporting Data.

### 3.5. Toxicity Studies

Toxicity prediction of patuletin was accomplished using Discovery studio 4.0 [63,64,65] and was addressed scrupulously in the Supporting Data.

### 3.6. MD Simulations

MD simulations of the patuletin–RdRp system were accomplished using the web-based CHARMM-GUI [66,67,68] and were addressed scrupulously in the Supporting Data.

## 4. Conclusions

This study presented the isolation and characterization of the rare flavonoid, patuletin, from the flowers of *Tagetes patula* growing in Egypt. Patuletin exhibited a high degree of structural similarity with **F86**, the ligand of SARS-CoV-2 RdRp. This similarity was verified by a 3D-Flexible alignment study. A molecular docking study indicated the excellent binding of patuletin inside the active pocket of RdRp with an energy of −20 kcal/mol that was almost the same as that of **F86** (−23 kcal/mol). Then, five MD simulation studies, over 100 ns, confirmed the accurate binding of patuletin in RdRp via the correct dynamic and energetic changes. Additionally, in silico ADMET studies indicated the general safety and drug-likeness of patuletin.

## Figures and Tables

**Table 1:** ^1^H and ^13^C data of patuletin (DMSO).

Position	δ ^1^H	δ ^13^C	Position	δ ^1^H (*J* = Hz)	δ ^13^C
2	-	147.1	10	-	103.3
3	-	135.6	1′	-	122.1
4	-	176.2	2′	7.70 d (*J* = 2)	115.8
5	-	152.0	3′	-	145.4
6	-	131.0	4′	-	147.9
7	-	151.6	5′	6.92 d (*J* = 8)	115.1
8	6.54 (s)	93.9	6′	7.56 dd (*J* = 2, *J* = 8)	120.3
9	-	157.3	O-CH_3_	3.79 (s)	60.3

**Table 2:** Structural properties of patuletin with the co-crystallized ligands.

Compound	ALog p	M-W	H.B-A	H.B-D	N-RB	N-R	N-AR	MFP-SA	Minimum Distance
**F86**	−1.502	371.243	11	5	4	3	2	0.612	0.758059
Patuletin	1.614	332.262	8	5	2	3	2	0.448	0.00
**PRD_002214**	2.453	680.791	8	5	18	3	2	0.273	1.5254
**GWS**	2.171	218.295	2	1	3	2	1	0.179	1.44878
**X77**	2.622	403.477	4	2	6	4	3	0.22	1.19065
**VXG**	0.711	233.263	3	1	2	2	1	0.237	1.31639
**1N7**	0.231	631.884	8	6	12	4	0	0.256	1.46363
**SAM**	−4.254	399.445	9	4	7	3	2	0.483	1.015
**Y95**	3.084	390.435	3	4	4	3	3	0.283	0.911118
**XT7**	3.873	504.687	5	5	9	5	3	0.224	1.35497

**Table 3:** Toxicity (predicted) of patuletin and remdesivir.

Test	Patuletin	Remdesivir
A-C	Non-Mutagen	Mutagen
R-TD_50_ (mg/kg)	7.45837	1.01218
R-MTD (g/kg)	1.05597	0.234965
R- LD_50_ (g/kg)	0.902102	0.308859
R- LOAEL (g/kg)	0.188616	0.0037911
O-Ir	Mild	None
S-Ir	Mild	Mild

## Data Availability

Not applicable.

## Supplementary Materials

Full method, spectral data and toxicity report can be downloaded at: https://www.mdpi.com/article/10.3390/plants11141886/s1.
