# Supplementary material for: The Computational Preventive Potential of the Rare Flavonoid, Patuletin, Isolated from Tagetes patula, against SARS-CoV-2
Source: Plants (Basel). 2022 Jul 20;11(14):1886. doi: 10.3390/plants11141886 (PMC9323967; doi:10.3390/plants11141886)
Supplement: Supplementary file 1 [file plants-11-01886-s001.zip › plants-1812855-supplementary.pdf]

Supporting materials

Isolation and *in silico* prohibitive activity of the rare flavonoid, patuletin, from *Tagetes patula* against SARS-CoV-2 RNA-dependent RNA polymerase

Ahmed M. Metwaly[1,2*], Eslam B. Elkaeed[3], Bshra A. Alsfouk[4], Abdelrahman M. Saleh[5], Ahmad E. Mostafa[1], Ibrahim. H. Eissa[5]*

[1] Pharmacognosy and Medicinal Plants Department, Faculty of Pharmacy (Boys), Al-Azhar University, Cairo, Egypt ametwaly@azhar.edu.eg; aemostafa@azhar.edu.eg
[2] Biopharmaceutical Products Research Department, Genetic Engineering and Biotechnology Research Institute, City of Scientific Research and Technological Applications (SRTA-City), Alexandria 21934, Egypt
[3] Department of Pharmaceutical Sciences, College of Pharmacy, AlMaarefa University, Riyadh 13713, Saudi Arabia ikaeed@mcst.edu.sa
[6] Department of Pharmaceutical Sciences, College of Pharmacy, Princess Nourah bint Abdulrahman University, P.O. Box 84428, Riyadh 11671, Saudi Arabia baalsfouk@pnu.edu.sa
[7] Pharmaceutical Medicinal Chemistry & Drug Design Department, Faculty of Pharmacy (Boys), Al-Azhar University, Cairo11884, Egypt ibrahimeissa@azhar.edu.eg; abdo.saleh240@azhar.edu.eg

***Corresponding authors:**

Ibrahim H. Eissa

Ahmed M. Metwaly

Content

Method	General
	Isolation
	Molecular Similarity
	Docking studies
	ADMET studies
	Toxicity studies
	Molecular dynamics
Spectral data	
Toxicity report	

Method

1. General experimental section

NMR spectra were carried out on a commercial instrument (Bruker Avance 500 MHz), chemical shifts (δ) are presented in parts per million (ppm) and re-calculated with respect to tetramethylsilane (TMS) (^{1}H) or carbon signals of deuterium solvents (^{13}C). Spin-spin coupling constants (J) are given in hertz (Hz). For column chromatography, silica gel 0.06-0.2 mm was used as the stationary phase. Silica gel 32-63 mesh and Sephadex LH-20 were used for column chromatography.

2. Isolation

To study the component composition of *Tagetes patula*, were used. Flowers were extracted with 70% ethanol three times. The extracts were combined, the solvent was evaporated on a rotary evaporator at 70 C, and the resulting extract afforded 210 gm of the total extract. The extract was suspended in water and fractionated against hexane, CH_2Cl_2, and n-butanol. Then, the butanol fraction (32 gm) was subjected to a silica gel column to provide 8 different fractions. Fraction 3 was further purified with Sephadex LH-20 to furnish 110 mg of patuletin.

Patuletin's chemical structure was determined by ^{1}H and ^{13}C NMR spectroscopy.

3. Molecular Similarity

Molecular Similarity of the tested compound against the nine co-crystallized ligands of SARS-Cov-2 was carried out calculated using Discovery studio 4.0. At first, the CHARMM force field was applied then the compound was prepared using prepare ligand protocol. Then, the tested compound was used as a reference compound while the co-crystallized ligands were used as a test set. The protocol was adjusted to give one output. The default molecular properties were applied. The following parameters were examined in patuletin and in the examined co-crystallized ligands (**F86, PRD_002214, GWS, X77, VXG, 1N7, SAM, Y95**, and **XT7**);

- Number of rotatable bonds,
- Number of rings,
- Number of aromatic rings,
- Number of hydrogen bond donor atoms,
- Number of hydrogen bond acceptor atoms,
- Partition coefficient (ALog p),
- Molecular weight (M. Wt), and
- Molecular fractional polar surface area (MFPSA).

4. Molecular Docking studies

Crystal structure of the examined protein was obtained from Protein Data Bank. The docking investigation was accomplished using MOE2014 software. At first, the crystal structure of target protein was prepared by removing water molecules. Then, the selected chain was protonated and subjected to minimization of energy process. Next, the active site of the target protein was defined. Structures of the tested compounds and the co-crystallized ligand were drawn using ChemBioDraw Ultra 14.0 and saved as MDL-SD format. Such file was opened using MOE to display the 3D structures which were protonated and subjected to energy minimization. Formerly, validation of the docking process was performed by docking the co-crystallized ligand against the isolated pocket of the active site. The produced RMSD value indicated the validity of the process. Finally, docking of the tested compounds was done through the dock option inserted in compute window. For each docked molecule, 30 docked poses were produced using ASE for scoring function and force field for refinement. The results of the docking process were then visualized using Discovery Studio 4.0 software.

5. ADMET

The following ADMET descriptors

- Absorption,
- Distribution,
- Metabolism,
- Excretion, and
- Toxicity

of the compounds were determined using Discovery studio 4.0. At first, the CHARMM force field was applied then the tested compounds were prepared and minimized according to the preparation of small molecule protocol. Then ADMET descriptors protocol was applied to carry out these studies.

6. Toxicity studies

The toxicity parameters of the tested compounds were calculated using Discovery studio 4.0. Remdesivir was used as a reference drug. At first, the CHARMM force field was applied then the compounds were prepared and minimized according to the preparation of small molecule protocol. Then different parameters were calculated from the toxicity prediction (extensible) protocol.

The examined parameter include

- Ames mutagenicity prediction,
- Carcinogenic potency in rats (R-TD_{50}),
- Rat maximum tolerated dose (R-MTD),
- Rat Oral LD_{50} (R- LD_{50}),
- Chronic (Lowest-observed-adverse-effect level) LOAEL in rats (R- LOAEL),

- Eye, ocular, irritation model (O-Ir), and
- Skin irritation model (S-Ir).

7. Molecular dynamics simulations

The system was prepared using the web-based CHARMM-GUI interface with the CHARMM36 force field. All the simulations were done using the NAMD 2.13 package. The TIP3P explicit solvation model was used, and the periodic boundary conditions were set with a dimension of the dimensions Å in x, y, and z, respectively. The parameters for the top docking results were generated using the CHARMM general force field Afterward, the system was neutralized using ---- (Cl^-/Na^+) ions. The MD protocols involved minimization, equilibration, and production. a 2 *fs* time step of integration was chosen for all MD simulations, the equilibration was carried in the canonical (*NVT*) ensemble, while the isothermal–isobaric (*NPT*) ensemble was for the production. Through the 100 *ns* of MD production, the pressure was set at 1 atm using the Nose´–Hoover Langevin piston barostat with a Langevin piston decay of 0.05 *ps* and a period of 0.1 *ps*. The temperature was set at 298.15 K using the Langevin thermostat. A distance cutoff of 12.0 Å was applied to short-range nonbonded interactions with a pair list distance of 16 Å, and Lennard Jones interactions were smoothly truncated at 8.0 Å. Long-range electrostatic interactions were treated using the particle-mesh Ewald (PME) method, where a grid spacing of 1.0 Å was used for all simulation cells. All covalent bonds involving hydrogen atoms were constrained using the SHAKE algorithm. For consistency, we have applied the same protocol for all MD simulations.

Spectral data NMR of compound 1

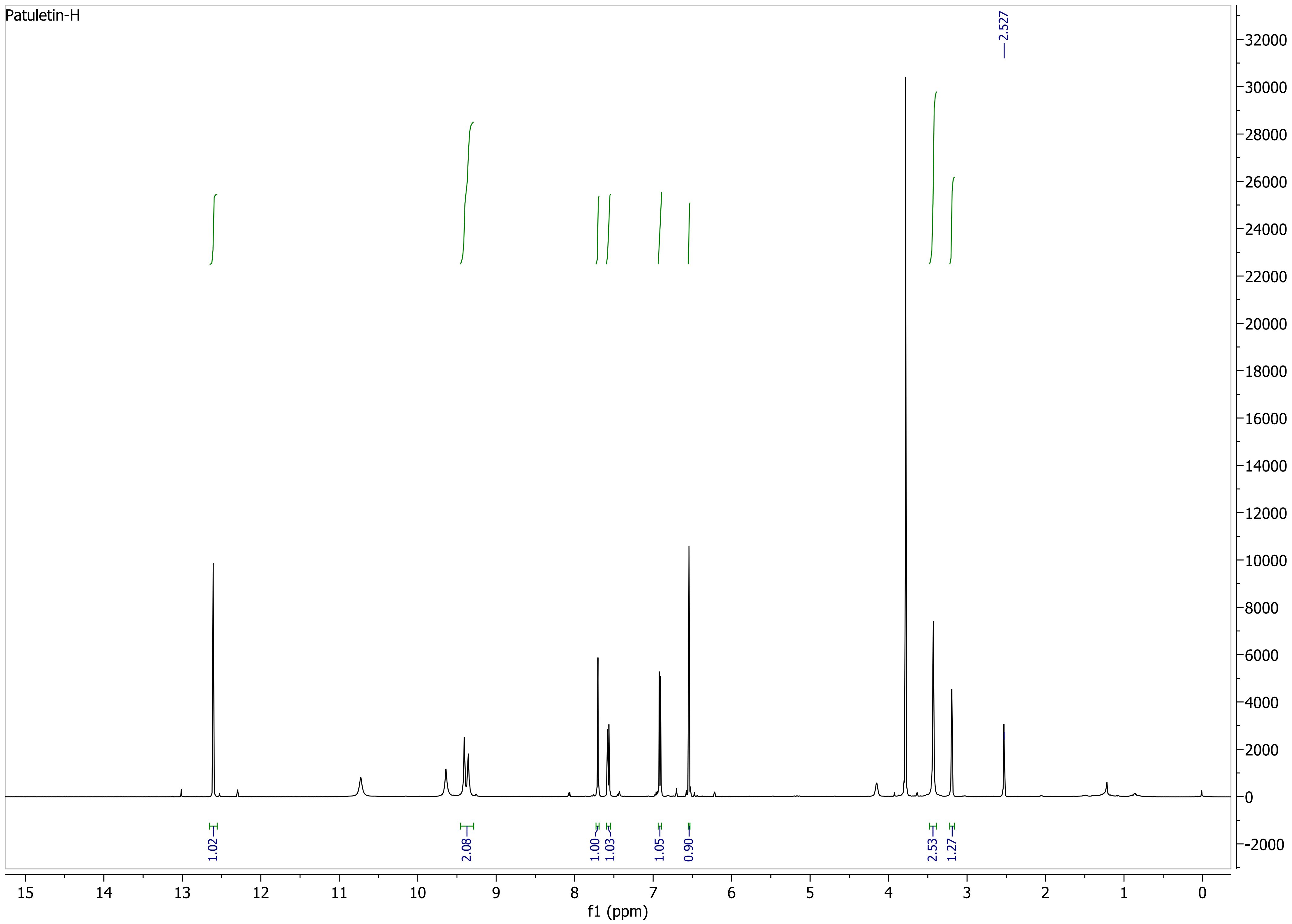

Patuletin-H
2.527
1.02
2.08
1.00
1.03
1.05
0.90
2.53
1.27
f1 (ppm)

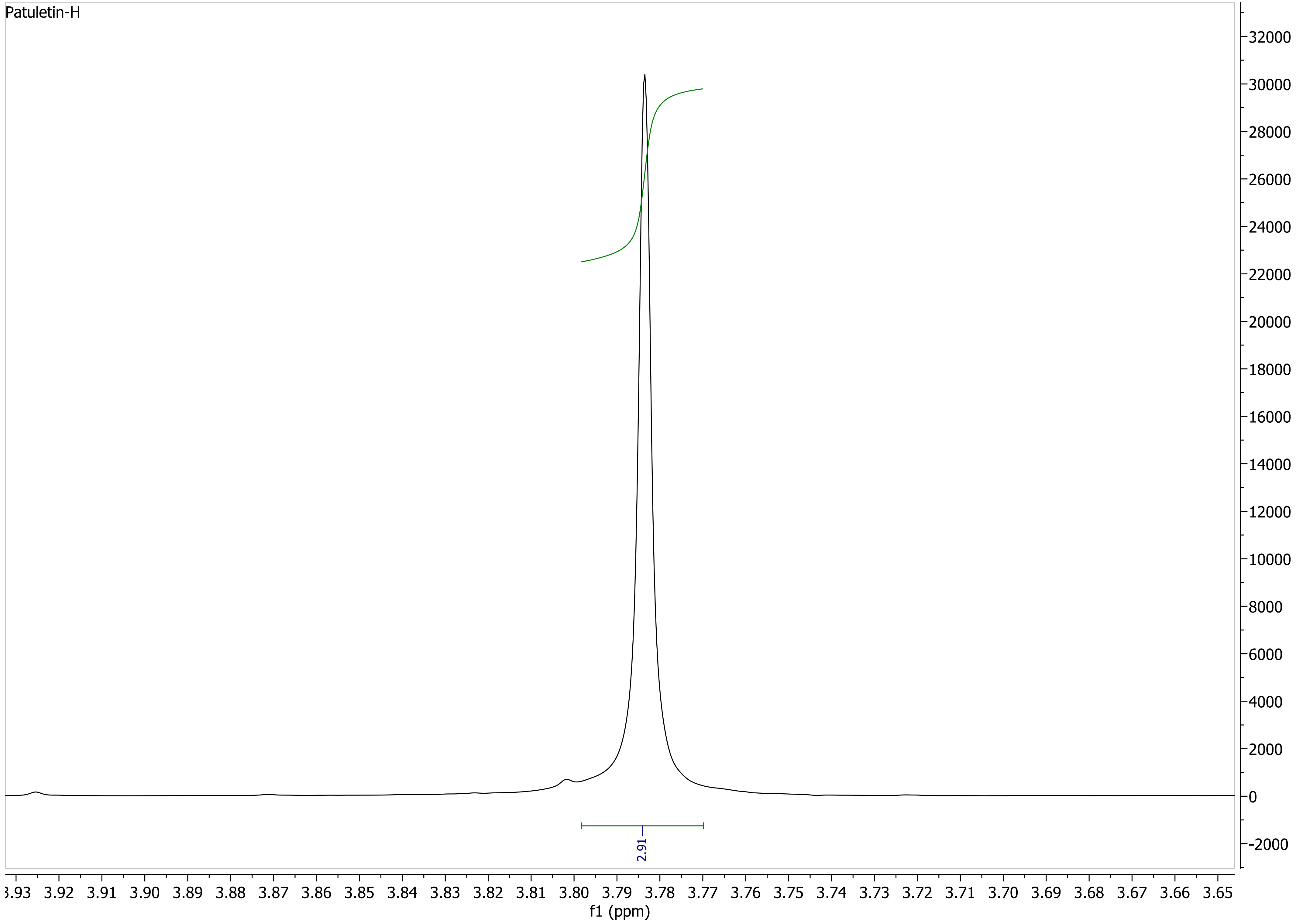

Patuletin-H
2.91
f1 (ppm)

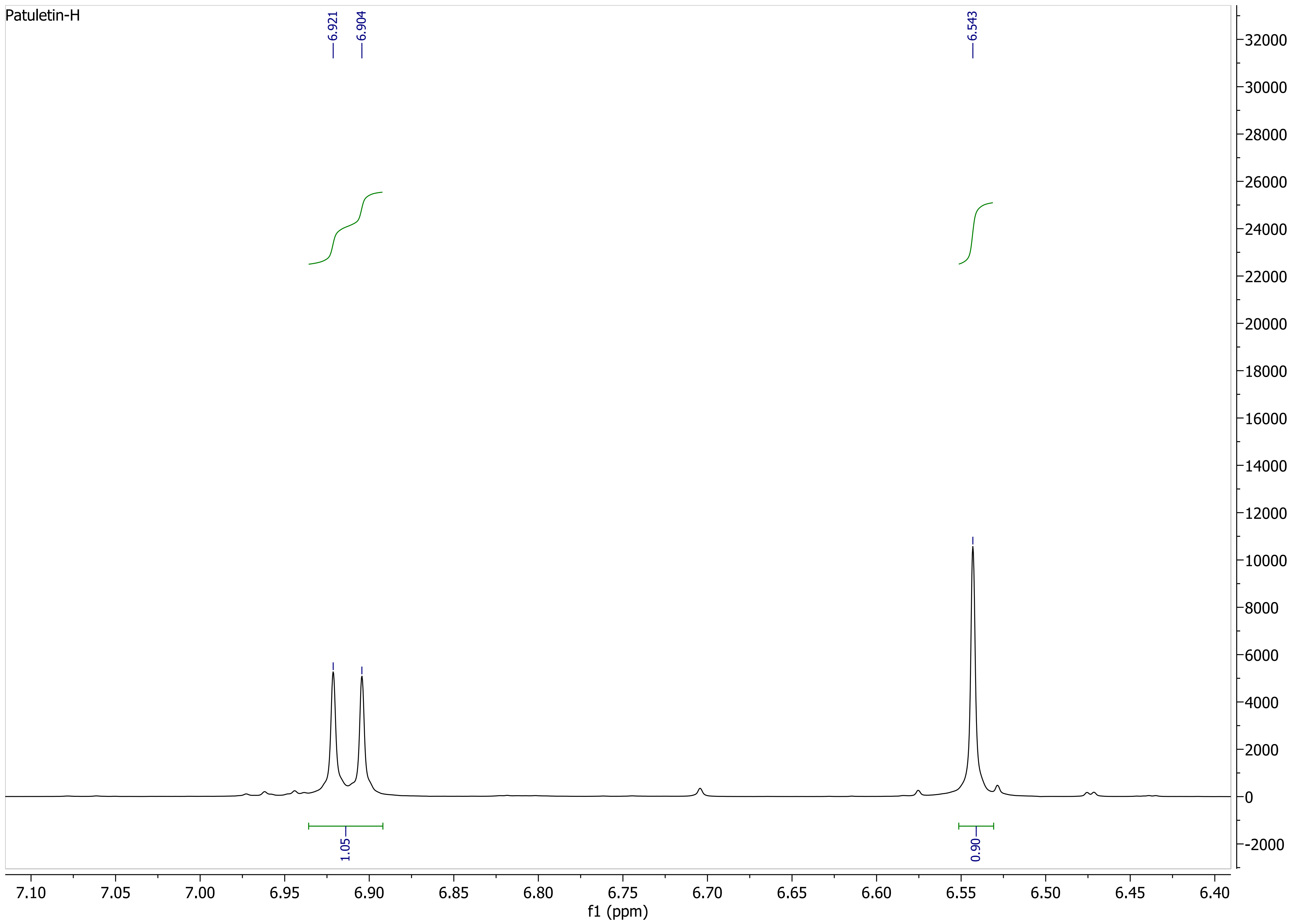
Patuletin-H
6.921
6.904
6.543
1.05
0.90
f1 (ppm)

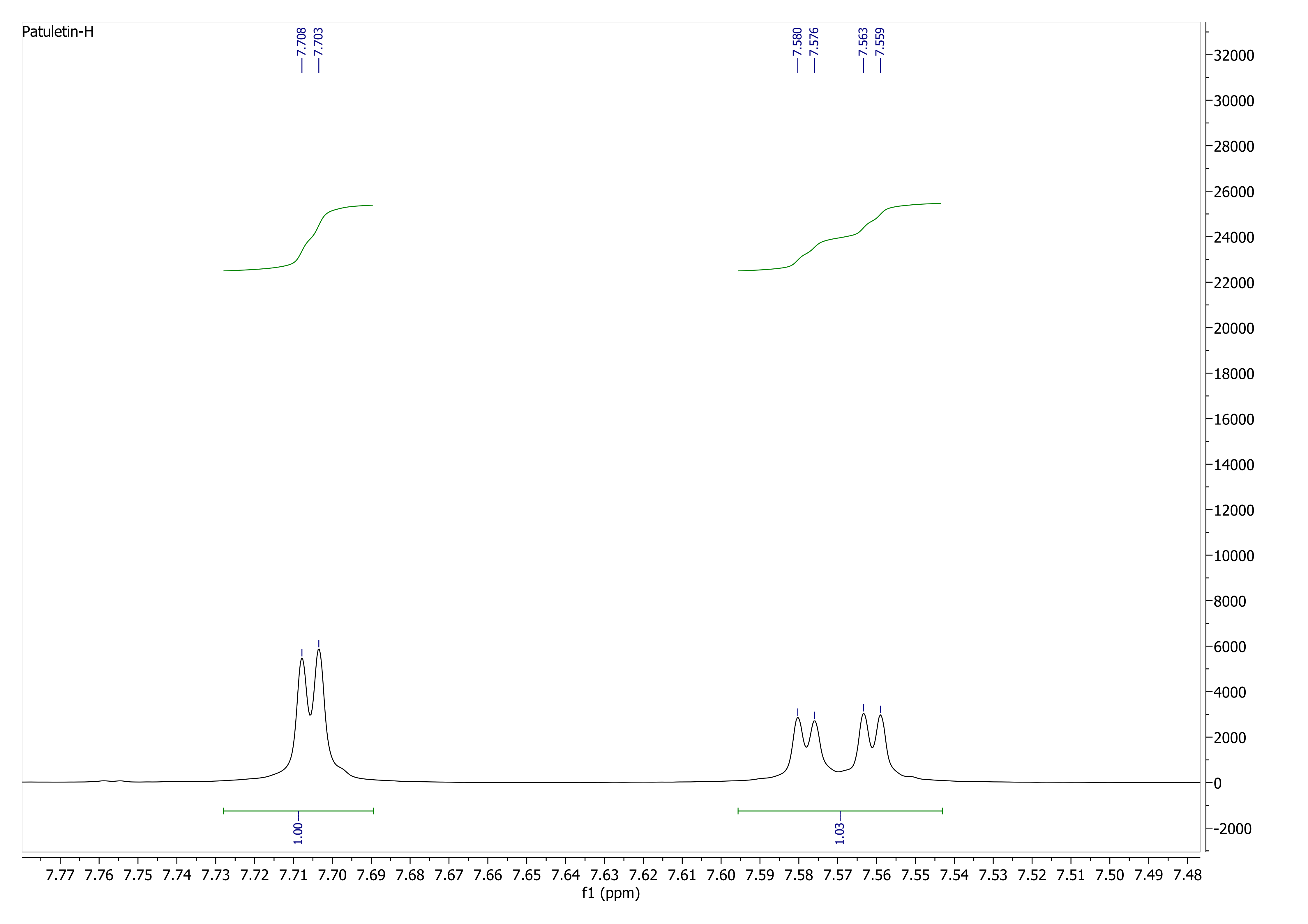
Patuletin-H
7.708
7.703
7.580
7.576
7.563
7.559
1.00
1.03
f1 (ppm)

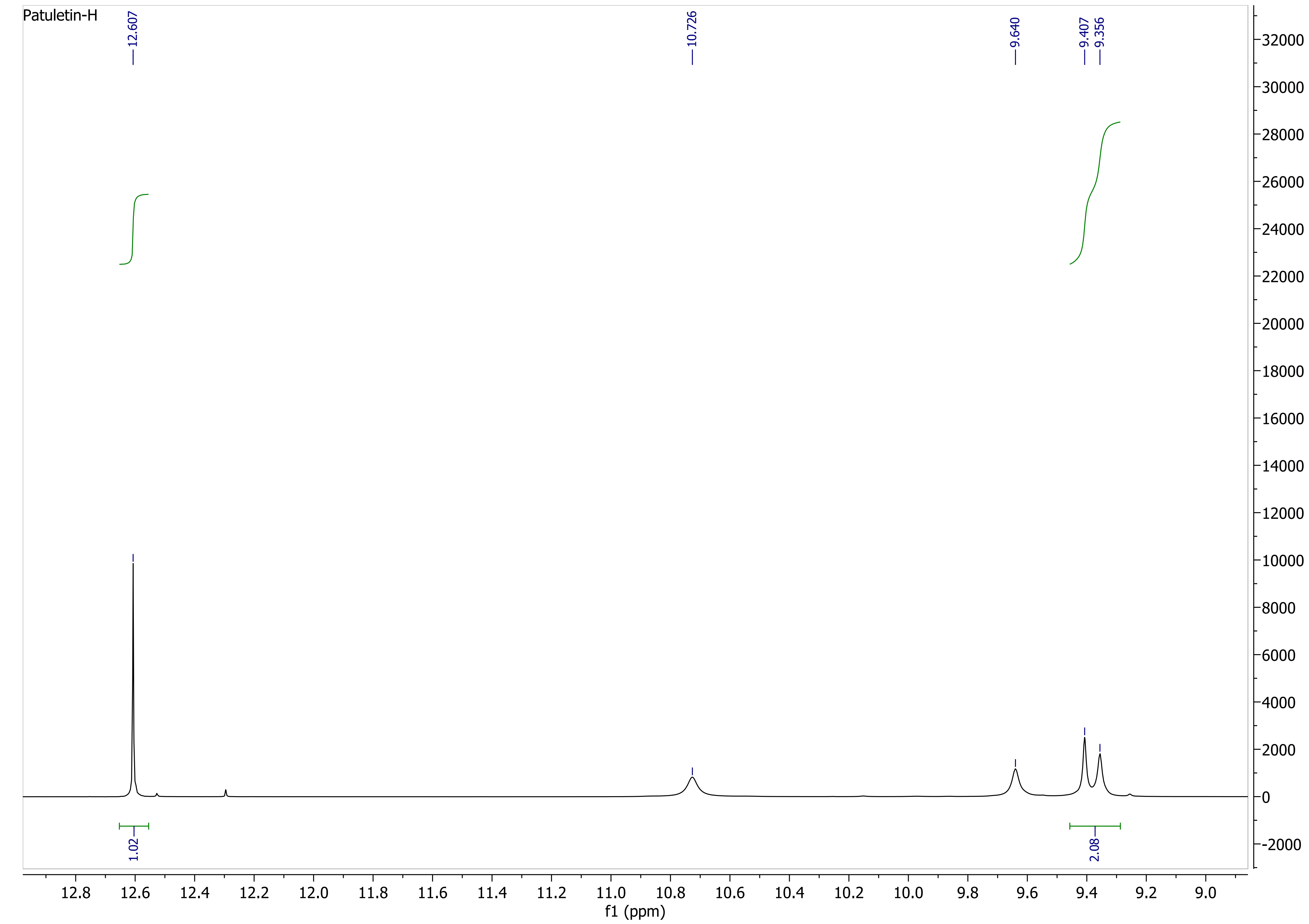
Patuletin-H
12.607
10.726
9.640
9.407
9.356
1.02
2.08
f1 (ppm)

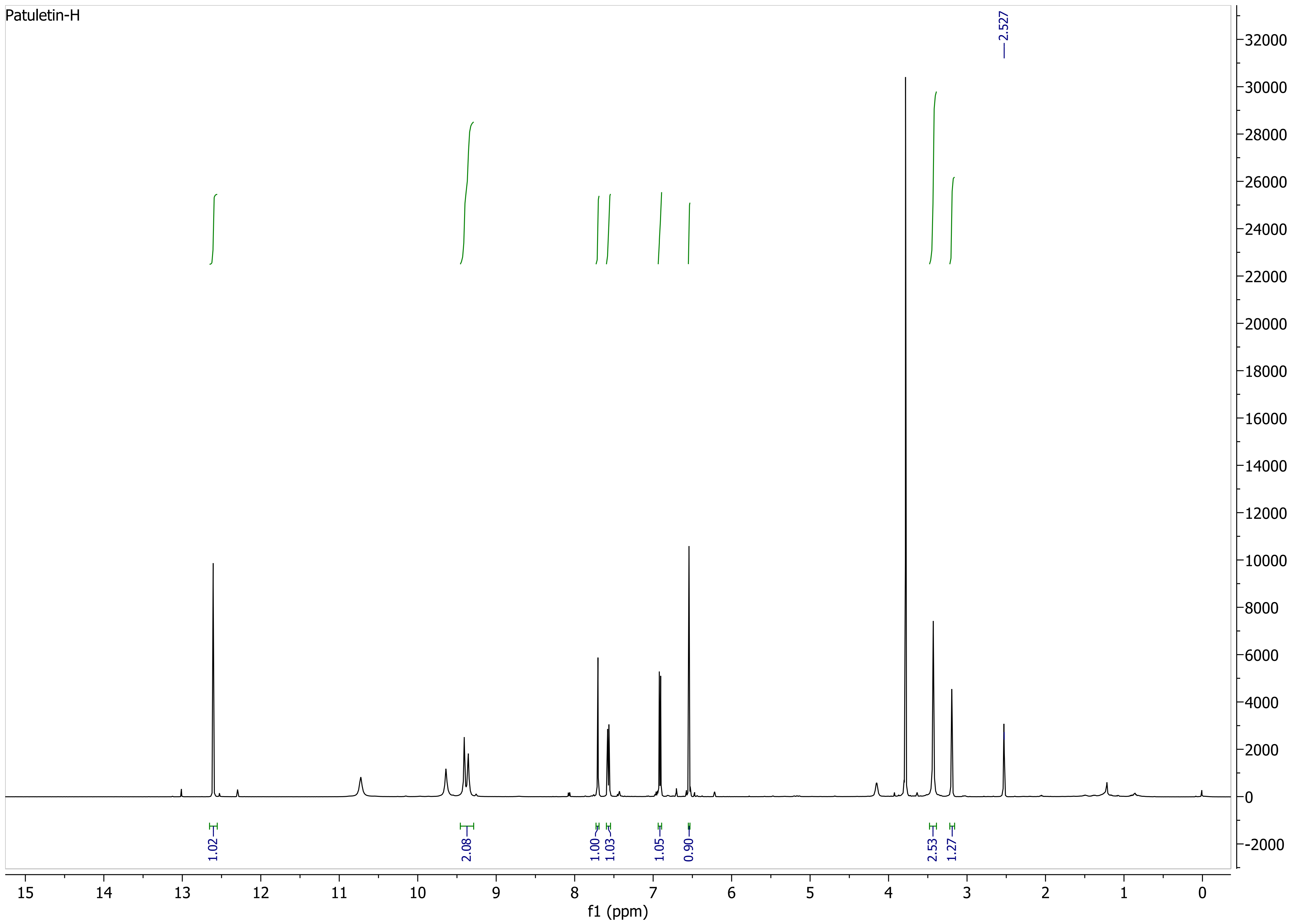
Patuletin-H
2.527
1.02
2.08
1.00
1.03
1.05
0.90
2.53
1.27
f1 (ppm)

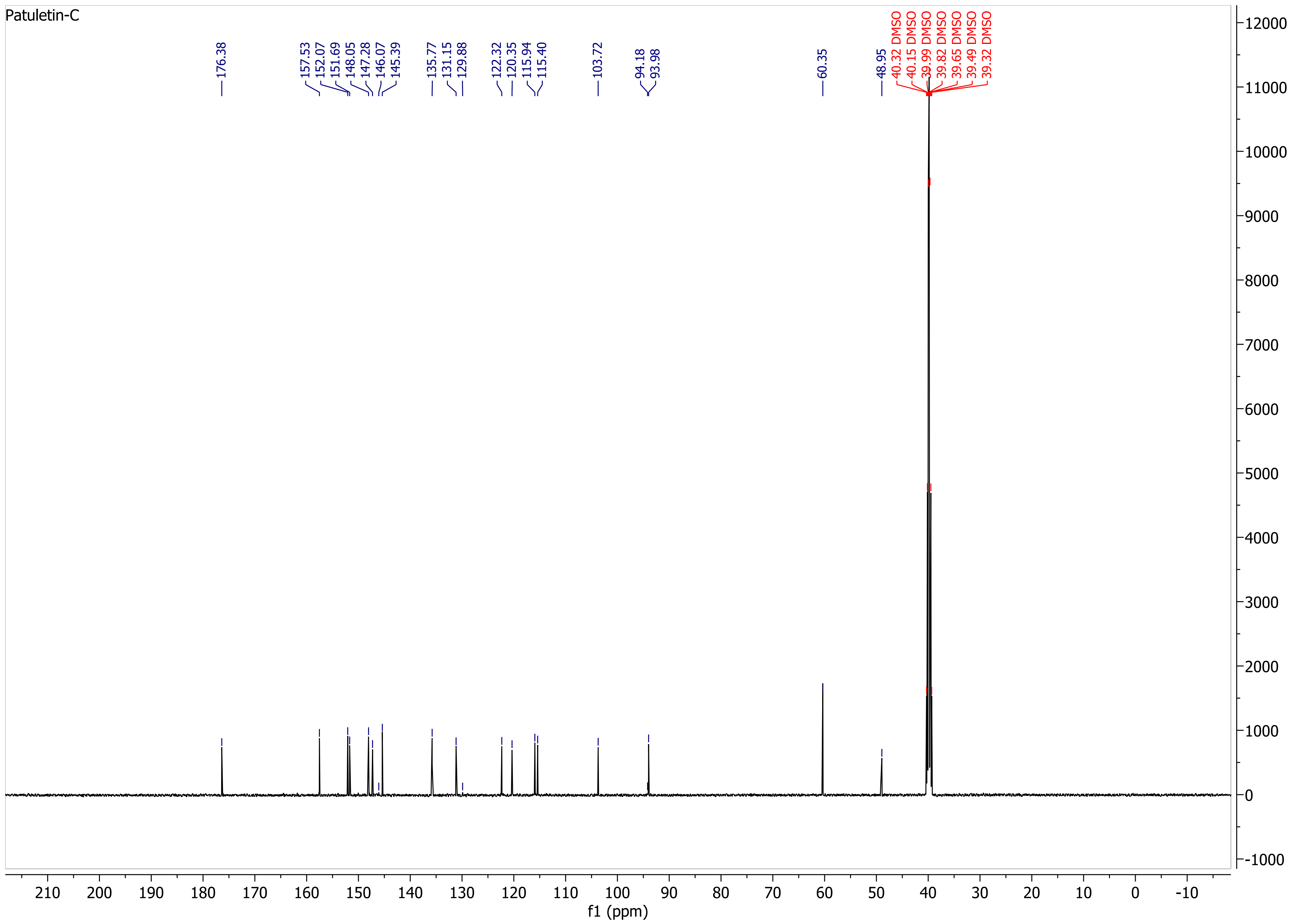

Patuletin-C
176.38
157.53
152.07
151.69
148.05
147.28
146.07
145.39
135.77
131.15
129.88
122.32
120.35
115.94
115.40
103.72
94.18
93.98
60.35
48.95
40.32 DMSO
40.15 DMSO
39.99 DMSO
39.82 DMSO
39.65 DMSO
39.49 DMSO
39.32 DMSO
f1 (ppm)

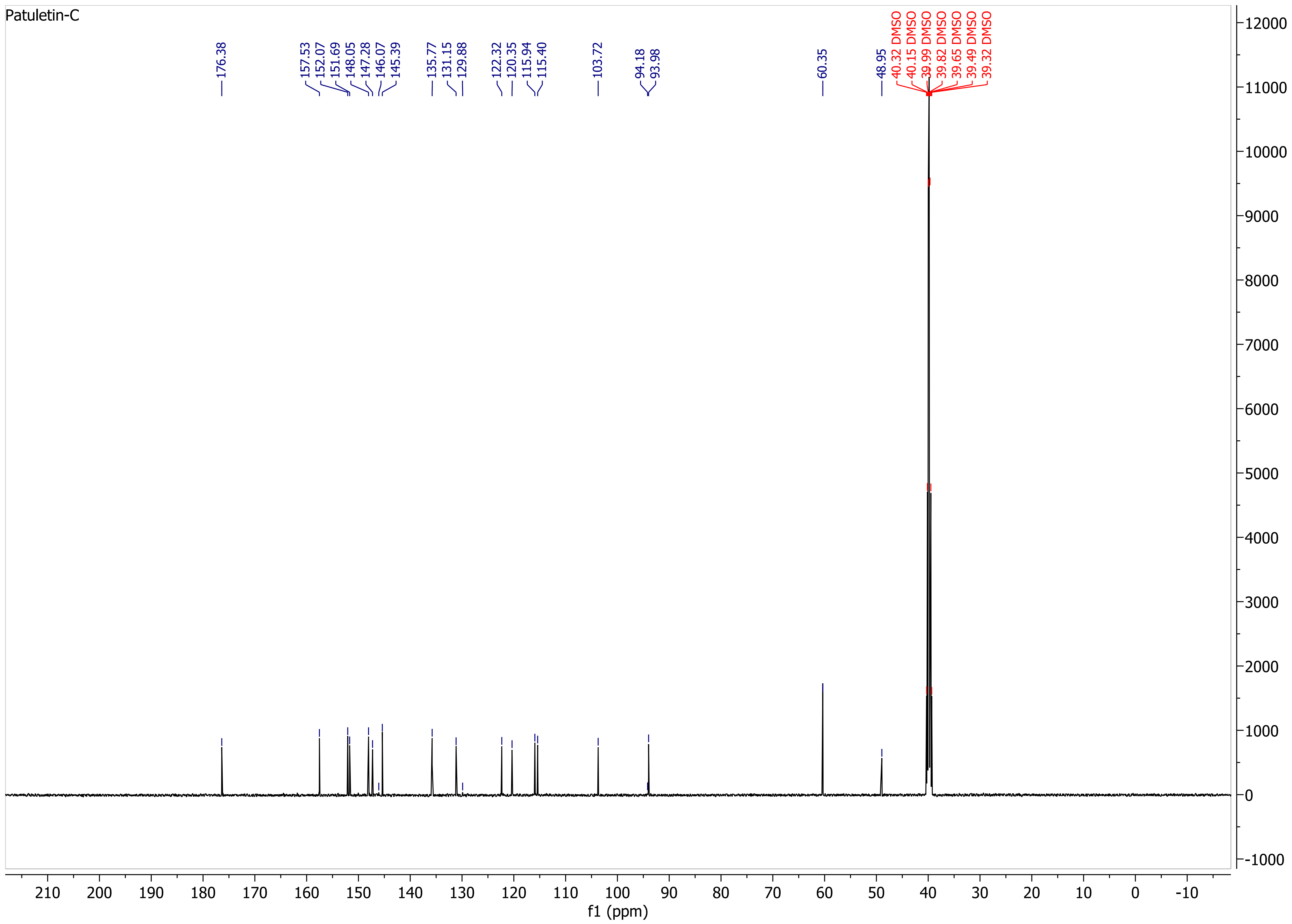
Patuletin-C
176.38
157.53
152.07
151.69
148.05
147.28
146.07
145.39
135.77
131.15
129.88
122.32
120.35
115.94
115.40
103.72
94.18
93.98
60.35
48.95
40.32 DMSO
40.15 DMSO
39.99 DMSO
39.82 DMSO
39.65 DMSO
39.49 DMSO
39.32 DMSO
f1 (ppm)

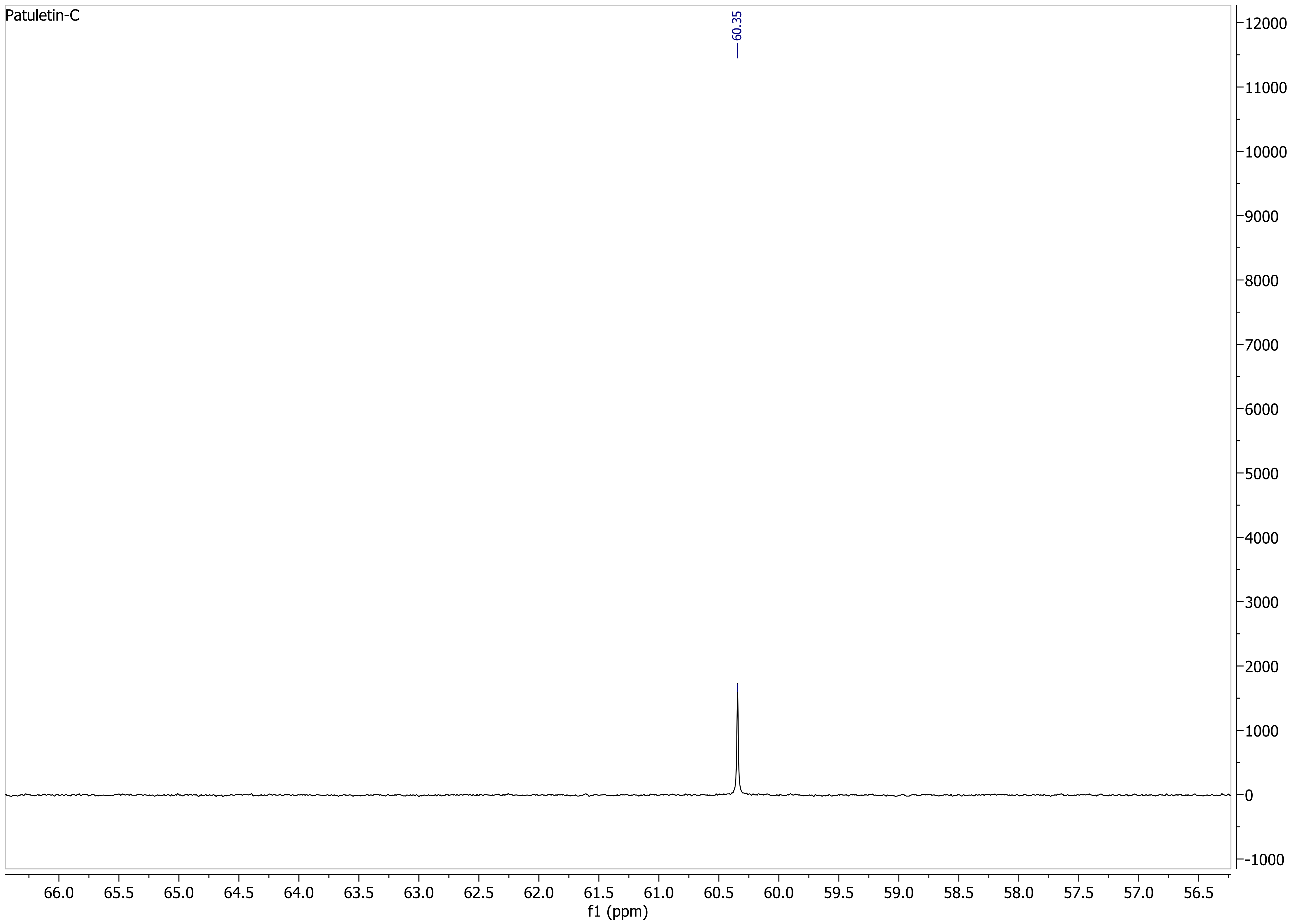
Patuletin-C
60.35
12000
11000
10000
9000
8000
7000
6000
5000
4000
3000
2000
1000
0
-1000
66.0
65.5
65.0
64.5
64.0
63.5
63.0
62.5
62.0
61.5
61.0
60.5
60.0
59.5
59.0
58.5
58.0
57.5
57.0
56.5
f1 (ppm)

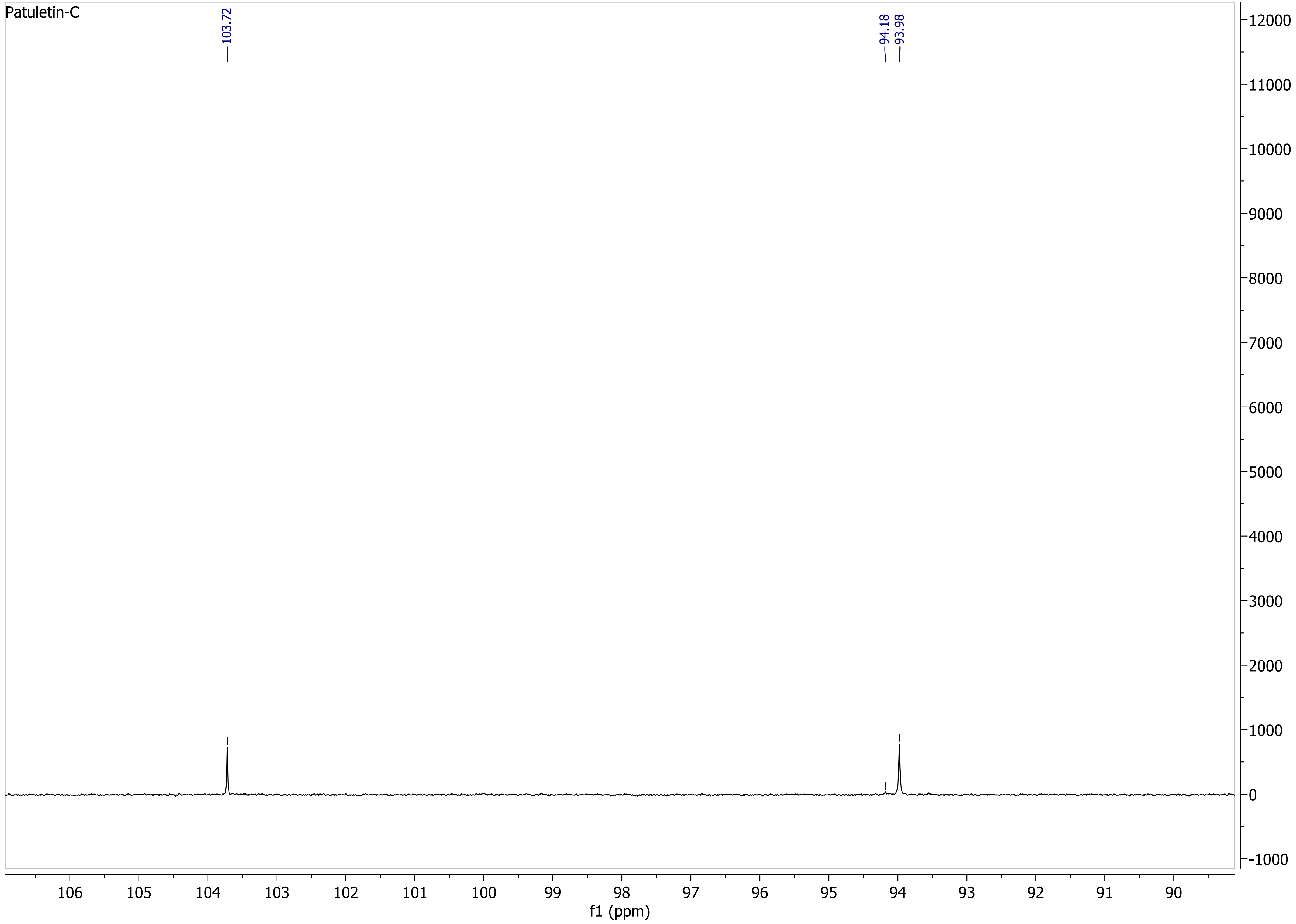
Patuletin-C
103.72
94.18
93.98
f1 (ppm)

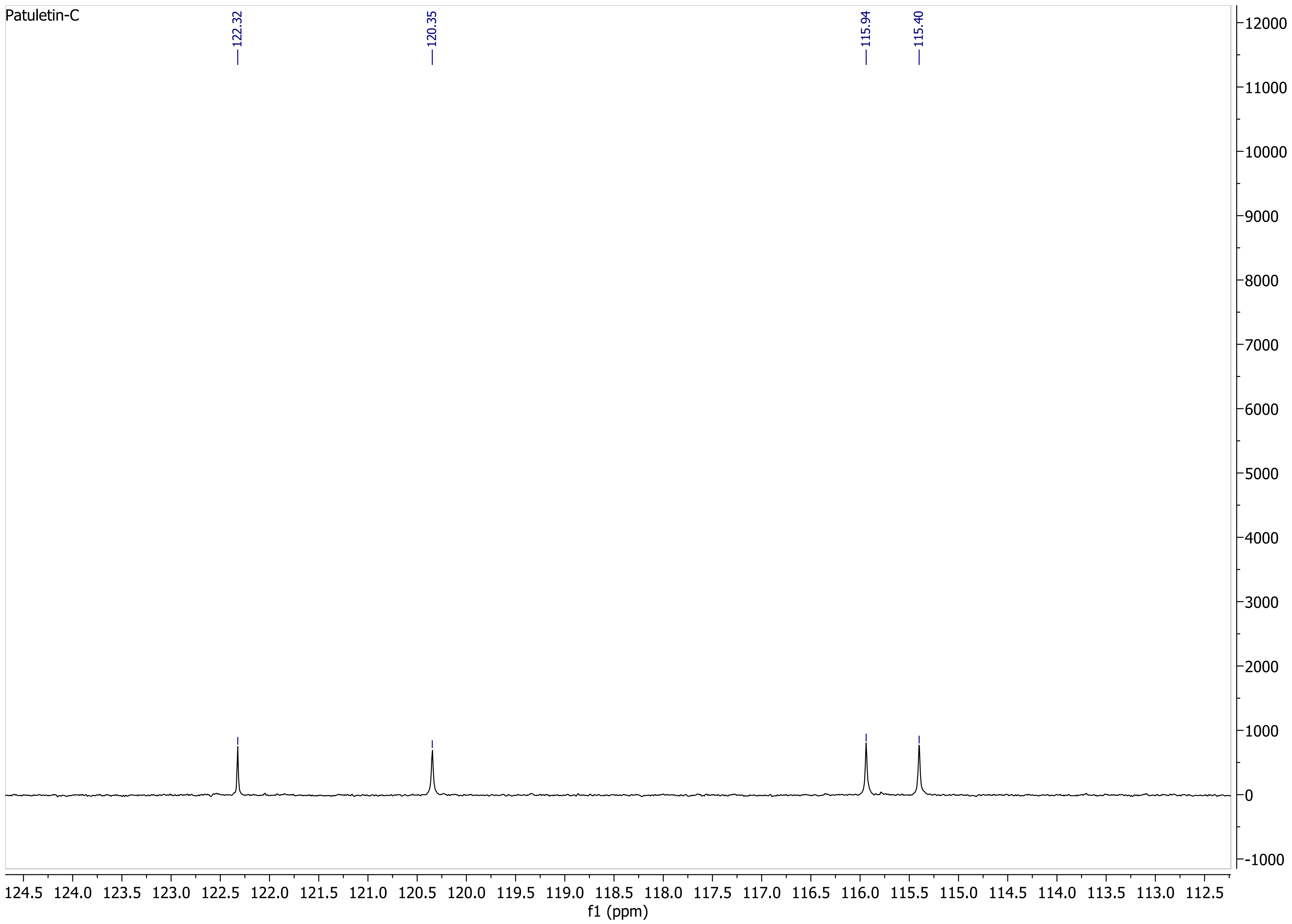
Patuletin-C
122.32
120.35
115.94
115.40
f1 (ppm)

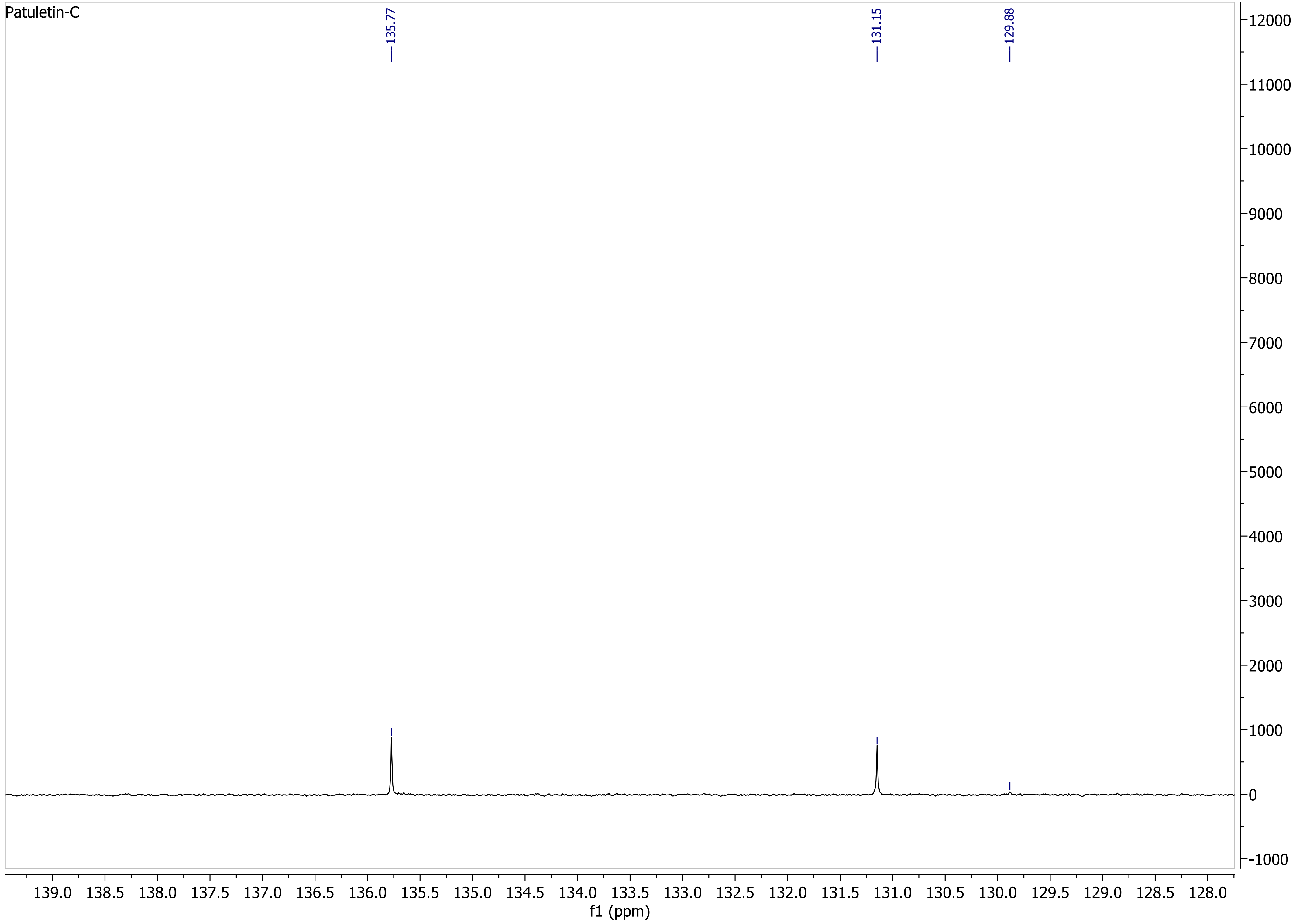

Patuletin-C
135.77
131.15
129.88
12000
11000
10000
9000
8000
7000
6000
5000
4000
3000
2000
1000
0
-1000
139.0 138.5 138.0 137.5 137.0 136.5 136.0 135.5 135.0 134.5 134.0 133.5 133.0 132.5 132.0 131.5 131.0 130.5 130.0 129.5 129.0 128.5 128.0
f1 (ppm)

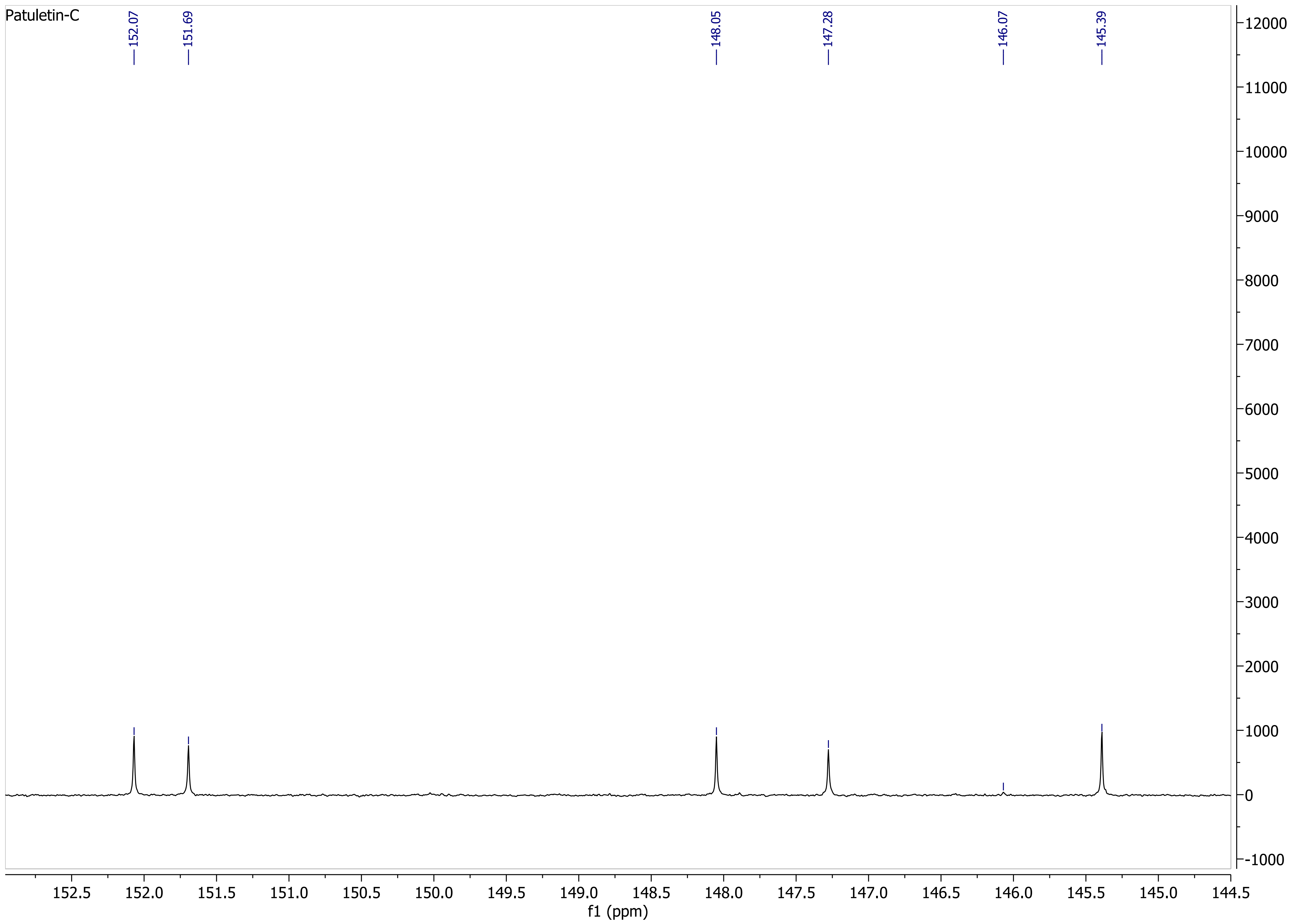
Patuletin-C
152.07
151.69
148.05
147.28
146.07
145.39
12000
11000
10000
9000
8000
7000
6000
5000
4000
3000
2000
1000
0
-1000
152.5
152.0
151.5
151.0
150.5
150.0
149.5
149.0
148.5
148.0
147.5
147.0
146.5
146.0
145.5
145.0
144.5
f1 (ppm)

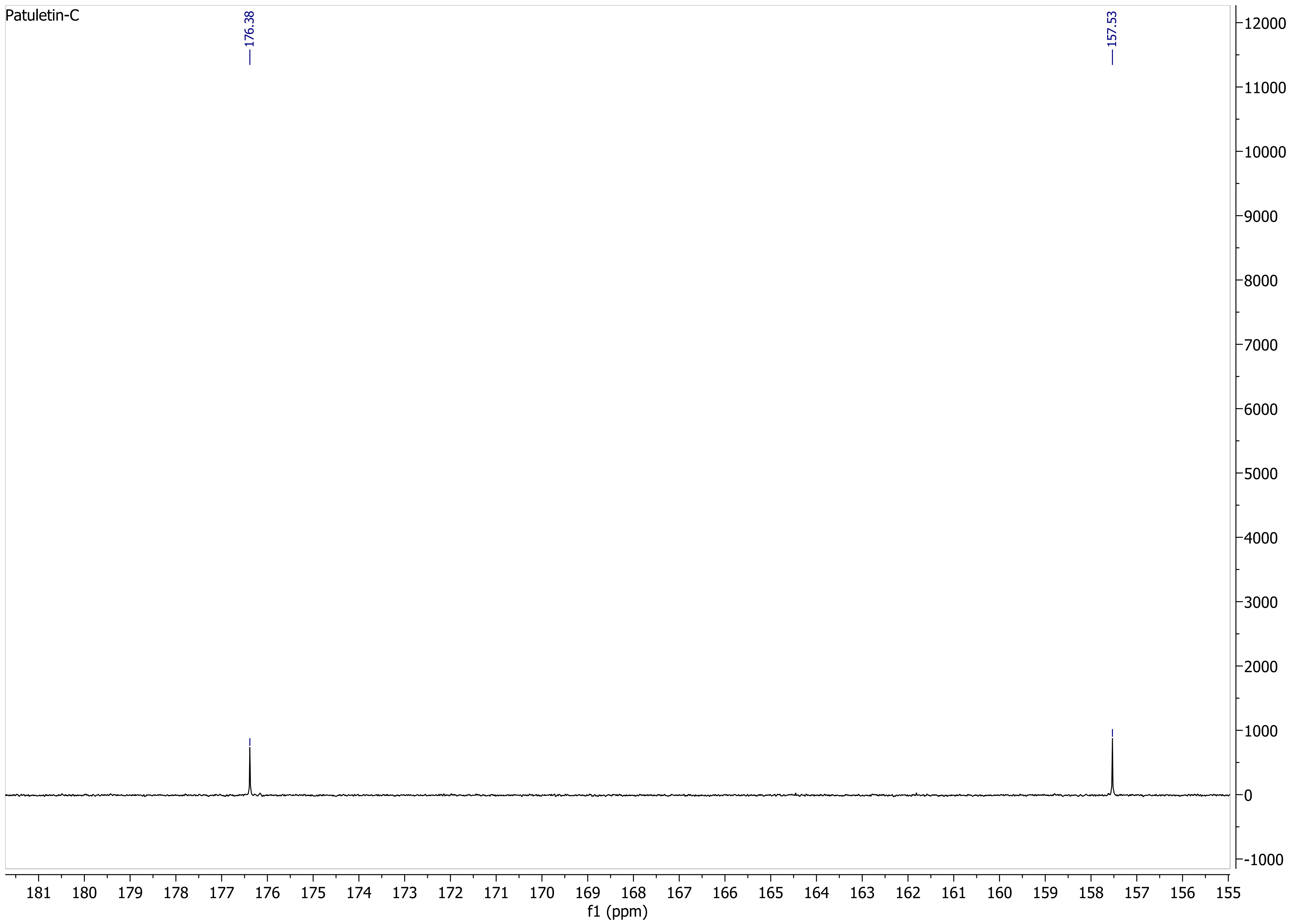
Patuletin-C
176.38
157.53
f1 (ppm)

Toxicity Report

Patuletin

TOPKAT_Mouse_Female_FDA_None_vs_Carcinogen

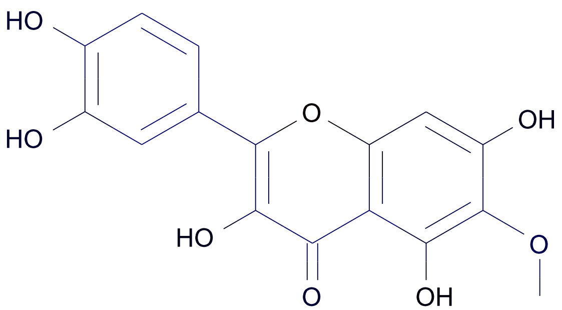

$C_{16}H_{12}O_8$
Molecular Weight: 332.26168
ALogP: 1.614
Rotatable Bonds: 2
Acceptors: 8
Donors: 5

Model Prediction

Prediction: Non-Carcinogen
Probability: 0.209
Enrichment: 0.651
Bayesian Score: -8.04
Mahalanobis Distance: 14.5
Mahalanobis Distance p-value: 2.3e-006

Prediction: Positive if the Bayesian score is above the estimated best cutoff value from minimizing the false positive and false negative rate.
Probability: The esimated probability that the sample is in the positive category. This assumes that the Bayesian score follows a normal distribution and is different from the prediction using a cutoff.
Enrichment: An estimate of enrichment, that is, the increased likelihood (versus random) of this sample being in the category.
Bayesian Score: The standard Laplacian-modified Bayesian score.
Mahalanobis Distance: The Mahalanobis distance (MD) is the distance to the center of the training data. The larger the MD, the less trustworthy the prediction.
Mahalanobis Distance p-value: The p-value gives the fraction of training data with an MD greater than or equal to the one for the given sample, assuming normally distributed data. The smaller the p-value, the less trustworthy the prediciton. For highly non-normal X properties (e.g., fingerprints), the MD p-value is wildly inaccurate.

Structural Similar Compounds

Name	Olsalazine	Minocycline	Tetracycline
Structure	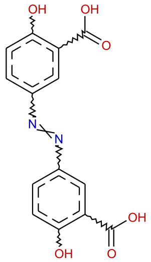	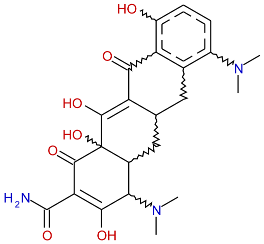	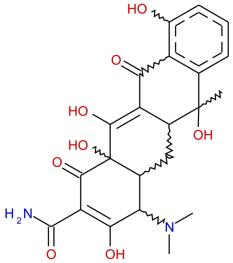
Actual Endpoint	Non-Carcinogen	Non-Carcinogen	Non-Carcinogen
Predicted Endpoint	Carcinogen	Non-Carcinogen	Non-Carcinogen
Distance	0.677	0.749	0.798
Reference	US FDA (Centre for Drug Eval.& Res./Off. Testing & Res.) Sept. 1997	US FDA (Centre for Drug Eval.& Res./Off. Testing & Res.) Sept. 1997	US FDA (Centre for Drug Eval.& Res./Off. Testing & Res.) Sept. 1997

Model Applicability

Unknown features are fingerprint features in the query molecule, but not found or appearing too infrequently in the training set.

1. All properties and OPS components are within expected ranges.
2. Unknown ECFP_2 feature: 1796421070: [*]OC(=C([*])[*])[c](:[*]):[*]

Feature Contribution

Top features for positive contribution

Fingerprint	Bit/Smiles	Feature Structure	Score	Carcinogen in training set
ECFP_6	2106656448	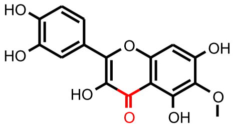 [*]C(=O)[*]	0.254	31 out of 77

ECFP_6	-560785749	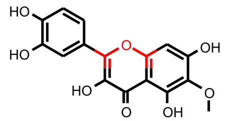 [*]C(=[*])O[c](:[*]):[*]	0.212	1 out of 2
ECFP_6	683445015	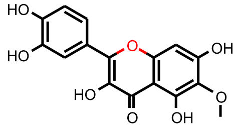 [*]O[*]	0.181	18 out of 48
**Top Features for negative contribution**				
**Fingerprint**	**Bit/Smiles**	**Feature Structure**	**Score**	**Carcinogen in training set**
ECFP_6	1717462980	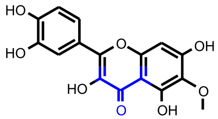 [*]C(=[*])C(=O)[c](:[*]):[*]	-1.25	0 out of 8
ECFP_6	-219423964	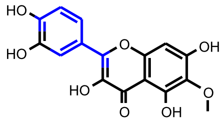 [*]C(=[*])[c]1:[cH]:[*]:[c]([*]):[cH]:[cH]:1	-0.935	0 out of 5
ECFP_6	1307307440	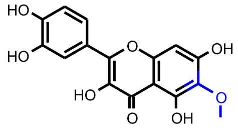 [*]:[c](:[*])OC	-0.558	4 out of 25

Remdesivir

TOPKAT_Mouse_Female_FDA_None_vs_Carcinogen

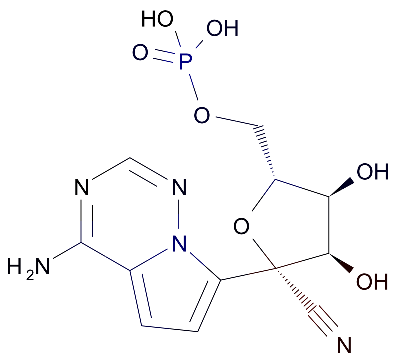

$C_{12}H_{14}N_5O_7P$
Molecular Weight: 371.24262
ALogP: -1.502
Rotatable Bonds: 4
Acceptors: 11
Donors: 5

Model Prediction

Prediction: Non-Carcinogen
Probability: 0.206
Enrichment: 0.642
Bayesian Score: -7.17
Mahalanobis Distance: 12.9
Mahalanobis Distance p-value: 0.00074

Prediction: Positive if the Bayesian score is above the estimated best cutoff value from minimizing the false positive and false negative rate.
Probability: The esimated probability that the sample is in the positive category. This assumes that the Bayesian score follows a normal distribution and is different from the prediction using a cutoff.
Enrichment: An estimate of enrichment, that is, the increased likelihood (versus random) of this sample being in the category.
Bayesian Score: The standard Laplacian-modified Bayesian score.
Mahalanobis Distance: The Mahalanobis distance (MD) is the distance to the center of the training data. The larger the MD, the less trustworthy the prediction.
Mahalanobis Distance p-value: The p-value gives the fraction of training data with an MD greater than or equal to the one for the given sample, assuming normally distributed data. The smaller the p-value, the less trustworthy the prediciton. For highly non-normal X properties (e.g., fingerprints), the MD p-value is wildly inaccurate.

Structural Similar Compounds

Name	Famotidine	Tetracycline	Oxytetracycline
Structure	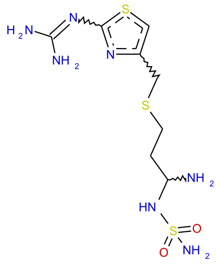	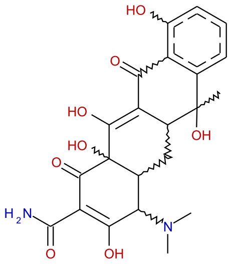	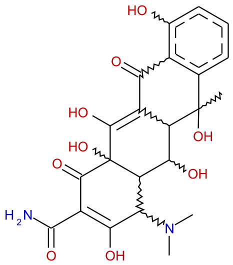
Actual Endpoint	Non-Carcinogen	Non-Carcinogen	Non-Carcinogen
Predicted Endpoint	Non-Carcinogen	Non-Carcinogen	Non-Carcinogen
Distance	0.846	0.848	0.870
Reference	US FDA (Centre for Drug Eval.& Res./Off. Testing & Res.) Sept. 1997	US FDA (Centre for Drug Eval.& Res./Off. Testing & Res.) Sept. 1997	US FDA (Centre for Drug Eval.& Res./Off. Testing & Res.) Sept. 1997

Model Applicability

Unknown features are fingerprint features in the query molecule, but not found or appearing too infrequently in the training set.

1. All properties and OPS components are within expected ranges.
2. Unknown ECFP_2 feature: 1126642748: [*]OP(=O)(O)O
3. Unknown ECFP_2 feature: -1250439909: [*]COP(=[*])([*])[*]
4. Unknown ECFP_2 feature: 1258791451: [*][C@@H]1[*][*]O[C@]1(C#[*])[c](:[*]):[*]
5. Unknown ECFP_2 feature: -1507082173: [*][c]1:[*]:[*]:[c](:[*]):n:1:n:[*]
6. Unknown ECFP_2 feature: -66263742: [*]C([*])([*])[c]1:n(:[*]):[*]:[*]:c:1

Feature Contribution

Top features for positive contribution				
**Fingerprint**	**Bit/Smiles**	**Feature Structure**	**Score**	**Carcinogen in training set**

ECFP_6	-1114776580	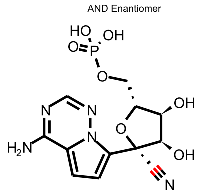 [*]C#[*]	0.755	11 out of 15
ECFP_6	-521596699	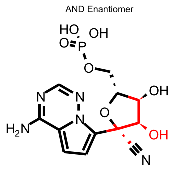 [*][C@@H]1[*][*]C([*])([*])[C@@H]1O	0.451	3 out of 5
ECFP_6	-264833661	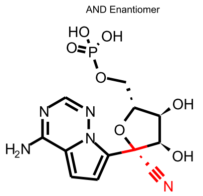 [*]C([*])([*])C#N	0.424	1 out of 1

Top Features for negative contribution

Fingerprint	Bit/Smiles	Feature Structure	Score	Carcinogen in training set
ECFP_6	1334415134	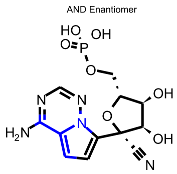 [*][c](:[*]):[c]1:[cH]:[*]:[*]:n:1:[*]	-0.935	0 out of 5
ECFP_6	2100964382	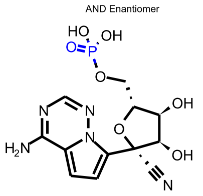 [*]P(=O)([*])[*]	-0.935	0 out of 5

ECFP_6	-826638028	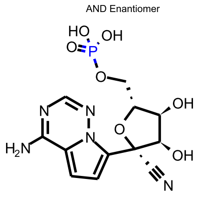<br>[*]P(=[*])([*])[*]	-0.935	0 out of 5

Patuletin

TOPKAT_Mouse_Male_FDA_None_vs_Carcinogen

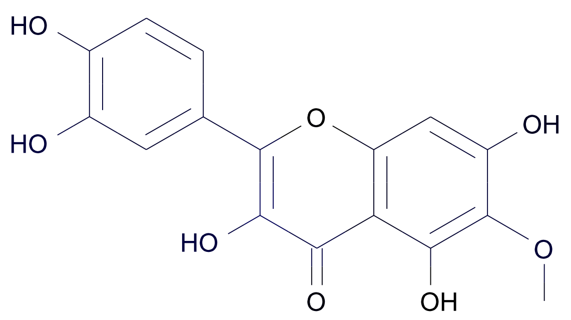

$C_{16}H_{12}O_8$
Molecular Weight: 332.26168
ALogP: 1.614
Rotatable Bonds: 2
Acceptors: 8
Donors: 5

Model Prediction

Prediction: Non-Carcinogen

Probability: 0.166

Enrichment: 0.565

Bayesian Score: -7.44

Mahalanobis Distance: 14.5

Mahalanobis Distance p-value: 9.1e-007

Prediction: Positive if the Bayesian score is above the estimated best cutoff value from minimizing the false positive and false negative rate.
Probability: The esimated probability that the sample is in the positive category. This assumes that the Bayesian score follows a normal distribution and is different from the prediction using a cutoff.
Enrichment: An estimate of enrichment, that is, the increased likelihood (versus random) of this sample being in the category.
Bayesian Score: The standard Laplacian-modified Bayesian score.
Mahalanobis Distance: The Mahalanobis distance (MD) is the distance to the center of the training data. The larger the MD, the less trustworthy the prediction.
Mahalanobis Distance p-value: The p-value gives the fraction of training data with an MD greater than or equal to the one for the given sample, assuming normally distributed data. The smaller the p-value, the less trustworthy the prediciton. For highly non-normal X properties (e.g., fingerprints), the MD p-value is wildly inaccurate.

Structural Similar Compounds

Name	Olsalazine	Minocycline	Tetracycline
Structure	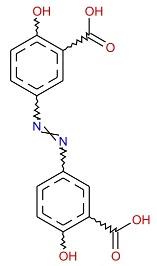	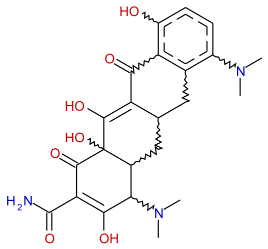	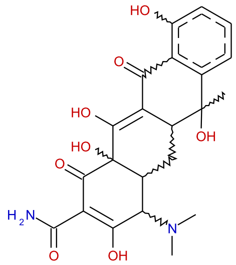
Actual Endpoint	Non-Carcinogen	Non-Carcinogen	Non-Carcinogen
Predicted Endpoint	Carcinogen	Non-Carcinogen	Non-Carcinogen
Distance	0.641	0.725	0.773
Reference	US FDA (Centre for Drug Eval.& Res./Off. Testing & Res.) Sept. 1997	US FDA (Centre for Drug Eval.& Res./Off. Testing & Res.) Sept. 1997	US FDA (Centre for Drug Eval.& Res./Off. Testing & Res.) Sept. 1997

Model Applicability

Unknown features are fingerprint features in the query molecule, but not found or appearing too infrequently in the training set.

1. All properties and OPS components are within expected ranges.
2. Unknown FCFP_2 feature: -1678245750: [*]OC(=C([*])[*])[c](:[*]):[*]

Feature Contribution

Top features for positive contribution

Fingerprint	Bit/Smiles	Feature Structure	Score	Carcinogen in training set
FCFP_6	1872154524	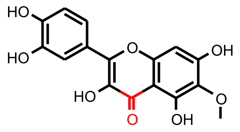 [*]C(=O)[*]	0.205	69 out of 213

FCFP_6	0	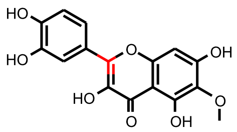<br>[*]C(=[*])[*]	0.114	90 out of 305
FCFP_6	1	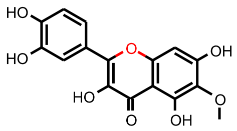<br>[*]O[*]	0.0783	76 out of 267

Top Features for negative contribution

Fingerprint	Bit/Smiles	Feature Structure	Score	Carcinogen in training set
FCFP_6	-1305924292	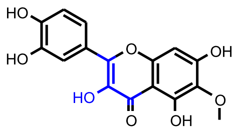<br>[*]C(=C(O)C(=[*])[*])[*]	-0.839	0 out of 5
FCFP_6	-1549192822	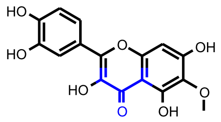<br>[*]C(=[*])C(=O)[c](:[*]):[*]	-0.489	3 out of 21
FCFP_6	523826990	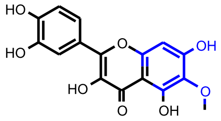<br>[*]O[c]1:[c]([*]):[*]:[c]([*]):[cH]:[c]:1<br>O	-0.423	0 out of 2

Remdesivir

TOPKAT_Mouse_Male_FDA_None_vs_Carcinogen

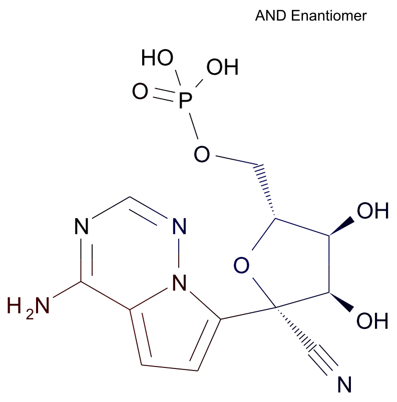

$C_{12}H_{14}N_5O_7P$
Molecular Weight: 371.24262
ALogP: -1.502
Rotatable Bonds: 4
Acceptors: 11
Donors: 5

Model Prediction

Prediction: Non-Carcinogen
Probability: 0.239
Enrichment: 0.812
Bayesian Score: -2.82
Mahalanobis Distance: 19.2
Mahalanobis Distance p-value: 7.81e-017

Prediction: Positive if the Bayesian score is above the estimated best cutoff value from minimizing the false positive and false negative rate.
Probability: The esimated probability that the sample is in the positive category. This assumes that the Bayesian score follows a normal distribution and is different from the prediction using a cutoff.
Enrichment: An estimate of enrichment, that is, the increased likelihood (versus random) of this sample being in the category.
Bayesian Score: The standard Laplacian-modified Bayesian score.
Mahalanobis Distance: The Mahalanobis distance (MD) is the distance to the center of the training data. The larger the MD, the less trustworthy the prediction.
Mahalanobis Distance p-value: The p-value gives the fraction of training data with an MD greater than or equal to the one for the given sample, assuming normally distributed data. The smaller the p-value, the less trustworthy the prediciton. For highly non-normal X properties (e.g., fingerprints), the MD p-value is wildly inaccurate.

Structural Similar Compounds

Name	Famotidine	Tetracycline	Ribavirin
Structure	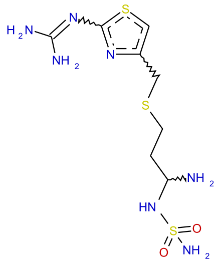	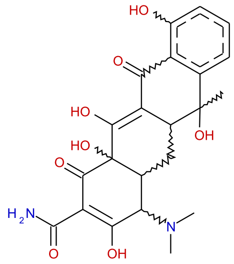	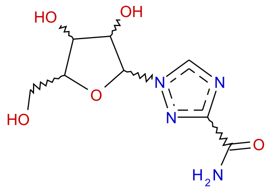
Actual Endpoint	Non-Carcinogen	Non-Carcinogen	Non-Carcinogen
Predicted Endpoint	Non-Carcinogen	Non-Carcinogen	Non-Carcinogen
Distance	0.813	0.843	0.860
Reference	US FDA (Centre for Drug Eval.& Res./Off. Testing & Res.) Sept. 1997	US FDA (Centre for Drug Eval.& Res./Off. Testing & Res.) Sept. 1997	US FDA (Centre for Drug Eval.& Res./Off. Testing & Res.) Sept. 1997

Model Applicability

Unknown features are fingerprint features in the query molecule, but not found or appearing too infrequently in the training set.

1. All properties and OPS components are within expected ranges.
2. Unknown FCFP_2 feature: 472180098: [*]OP(=O)(O)O
3. Unknown FCFP_2 feature: -332197802: [*][c]1:[*]:[*]:[c](:[*]):n:1:n:[*]

Feature Contribution

Top features for positive contribution

Fingerprint	Bit/Smiles	Feature Structure	Score	Carcinogen in training set
FCFP_6	-450797925	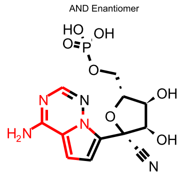 N[c]1:n:[cH]:[*]:n2:[*]:[*]:[cH]:[c]:1:2	0.676	2 out of 2

FCFP_6	-1151884458	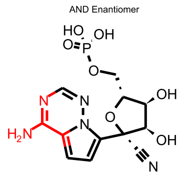<br>[*]:n:[c](N):[c](:[*]<br>):[*]	0.348	6 out of 15
FCFP_6	-1280036918	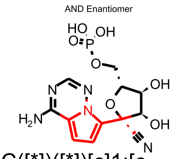<br>[*]C([*])([*])[c]1:[c<br>H]:[cH]:[c](:[*]):n:<br>1:[*]	0.333	7 out of 18

Top Features for negative contribution

**Fingerprint**	**Bit/Smiles**	**Feature Structure**	**Score**	**Carcinogen in training set**
FCFP_6	-124685461	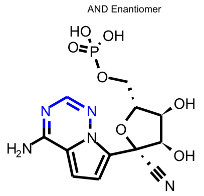<br>[*]:n:[cH]:n:[*]	-0.731	1 out of 12
FCFP_6	-1277879912	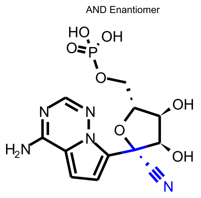<br>[*]C([*])([*])C#N	-0.582	0 out of 3
FCFP_6	422052003	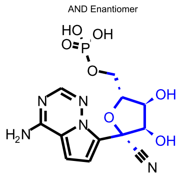<br>[*]C[C@H]1OC([*])([*]<br>)[C@H](O)[C@@H]1O	-0.582	0 out of 3

Patuletin

TOPKAT_Ocular_Irritancy_Mild_vs_Moderate_Severe

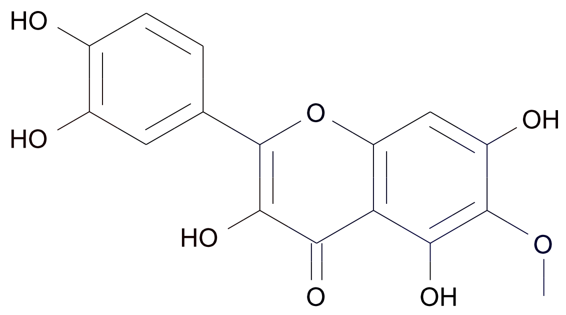

$C_{16}H_{12}O_8$
Molecular Weight: 332.26168
ALogP: 1.614
Rotatable Bonds: 2
Acceptors: 8
Donors: 5

Structural Similar Compounds

Name	ANTHRAQUINONE; 1;5-DIAMINO-4;8-DIHYDROXY-3-(p-METHOXYPHENYL)-	ANTHRAQUINONE; 1;4;5;8-TETRAHYDROXY-	2-Naphthalenesulfonic acid; 5;6'-iminobis(1-hydroxy-
Structure	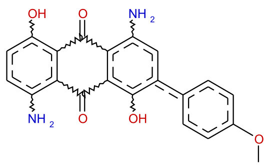	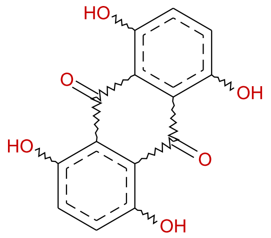	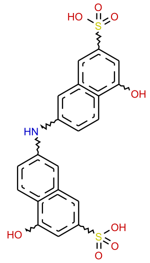
Actual Endpoint	Mild	Mild	Mild
Predicted Endpoint	Mild	Mild	Mild
Distance	0.727	0.754	0.760
Reference	28ZPAK 245;72	28ZPAK-;104;72	Prehled Prumyslove Toxikologie; Organicke Latky; Marhold; J. pp 1065;86

Model Prediction

Prediction: Mild
Probability: 0.805
Enrichment: 1.17
Bayesian Score: -0.821
Mahalanobis Distance: 13.3
Mahalanobis Distance p-value: 3.72e-008

Prediction: Positive if the Bayesian score is above the estimated best cutoff value from minimizing the false positive and false negative rate.
Probability: The esimated probability that the sample is in the positive category. This assumes that the Bayesian score follows a normal distribution and is different from the prediction using a cutoff.
Enrichment: An estimate of enrichment, that is, the increased likelihood (versus random) of this sample being in the category.
Bayesian Score: The standard Laplacian-modified Bayesian score.
Mahalanobis Distance: The Mahalanobis distance (MD) is the distance to the center of the training data. The larger the MD, the less trustworthy the prediction.
Mahalanobis Distance p-value: The p-value gives the fraction of training data with an MD greater than or equal to the one for the given sample, assuming normally distributed data. The smaller the p-value, the less trustworthy the prediciton. For highly non-normal X properties (e.g., fingerprints), the MD p-value is wildly inaccurate.

Model Applicability

Unknown features are fingerprint features in the query molecule, but not found or appearing too infrequently in the training set.

1. All properties and OPS components are within expected ranges.
2. Unknown FCFP_2 feature: -1678245750: [*]OC(=C([*])[*])[c](:[*]):[*]
3. Unknown FCFP_2 feature: -1305924292: [*]C(=C(O)C(=[*])[*])[*]

Feature Contribution

Top features for positive contribution				
Fingerprint	Bit/Smiles	Feature Structure	Score	Moderate_Severe in training set

FCFP_10	-548632217	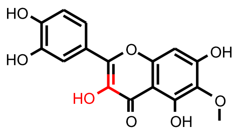<br>[*]C(=[*])O	0.319	54 out of 59
FCFP_10	7	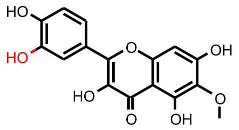<br>[*]O	0.219	117 out of 142
FCFP_10	1727347865	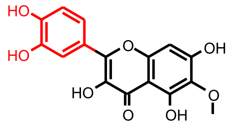<br>[*][c]1:[cH]:[cH]:[c](O):[c](O):[cH]:1	0.186	1 out of 1

Top Features for negative contribution

Fingerprint	Bit/Smiles	Feature Structure	Score	Moderate_Severe in training set
FCFP_10	-1977641857	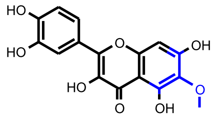<br>[*][c](:[*]):[c](OC):[c]([*]):[*]	-0.78	4 out of 15
FCFP_10	-1099193755	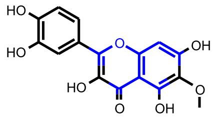<br>[*]C1=[*]C(=[*])[c]2:[c]([*]):[*]:[c]([*]):[cH]:[c]:2O1	-0.361	2 out of 5

FCFP_10	136627117	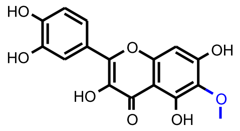 [*]OC	-0.316	46 out of 96

Remdesivir

TOPKAT_Ocular_Irritancy_Mild_vs_Moderate_Severe

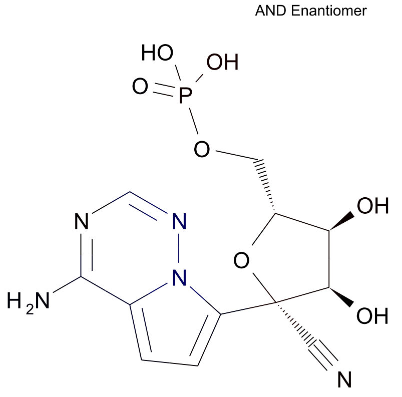

$C_{12}H_{14}N_5O_7P$

Molecular Weight: 371.24262

ALogP: -1.502

Rotatable Bonds: 4

Acceptors: 11

Donors: 5

Structural Similar Compounds

Name	1;3;6-NAPHTHALENE TRISULFONIC ACID;7-AMINO-	Methanol; (s-triazine-2;4;6-triyltrinitrilo)hexa-	2;2'-Biphenyldisulfonic acid; 4;4'-diamino-
Structure	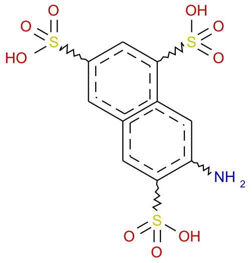	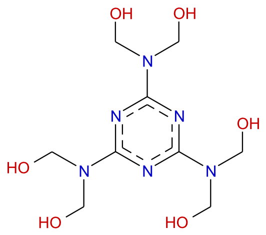	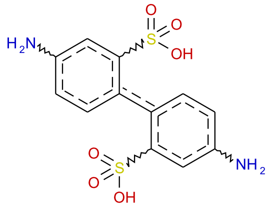
Actual Endpoint	Mild	Moderate_Severe	Mild
Predicted Endpoint	Mild	Moderate_Severe	Mild
Distance	0.776	0.802	0.878
Reference	28ZPAK-;190;72	Prehled Prumyslove Toxikologie; Organicke Latky; Marhold; J. -;876;86	Prehled Prumyslove Toxikologie; Organicke Latky; Marhold; J. pp 1061;86

Model Prediction

Prediction: Mild

Probability: 0.789

Enrichment: 1.15

Bayesian Score: -1.39

Mahalanobis Distance: 13.8

Mahalanobis Distance p-value: 1.42e-009

Prediction: Positive if the Bayesian score is above the estimated best cutoff value from minimizing the false positive and false negative rate.
Probability: The esimated probability that the sample is in the positive category. This assumes that the Bayesian score follows a normal distribution and is different from the prediction using a cutoff.
Enrichment: An estimate of enrichment, that is, the increased likelihood (versus random) of this sample being in the category.
Bayesian Score: The standard Laplacian-modified Bayesian score.
Mahalanobis Distance: The Mahalanobis distance (MD) is the distance to the center of the training data. The larger the MD, the less trustworthy the prediction.
Mahalanobis Distance p-value: The p-value gives the fraction of training data with an MD greater than or equal to the one for the given sample, assuming normally distributed data. The smaller the p-value, the less trustworthy the prediciton. For highly non-normal X properties (e.g., fingerprints), the MD p-value is wildly inaccurate.

Model Applicability

Unknown features are fingerprint features in the query molecule, but not found or appearing too infrequently in the training set.

1. OPS PC17 out of range. Value: 4.6782. Training min, max, SD, explained variance: -4.348, 3.9505, 1.094, 0.0146.
2. Unknown FCFP_2 feature: 472180098: [*]OP(=O)(O)O
3. Unknown FCFP_2 feature: -836603894: [*][C@@H]1[*][*]O[C@]1(C#[*])[c](:[*]):[*]
4. Unknown FCFP_2 feature: -124685461: [*]:n:c:n:[*]
5. Unknown FCFP_2 feature: -1151884458: [*]:n:[c](N):[c](:[*]):[*]

Feature Contribution

Top features for positive contribution

Fingerprint	Bit/Smiles	Feature Structure	Score	Moderate_Severe in training set

FCFP_10	1070061035	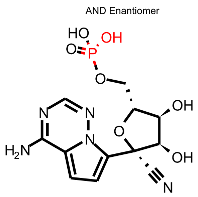 [*]P(=[*])([*])O	0.239	284 out of 338
FCFP_10	-1539132615	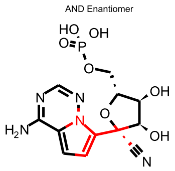 [*]C([*])([*])[c]1:[cH]:[*]:[*]:n:1:[*]	0.224	11 out of 13
FCFP_10	-1043250487	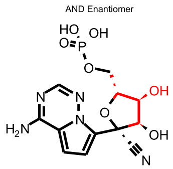 [*][C@H]1[*][*][C@H]([*])[C@H]1O	0.22	62 out of 75

Top Features for negative contribution

Fingerprint	Bit/Smiles	Feature Structure	Score	Moderate_Severe in training set
FCFP_10	4427049	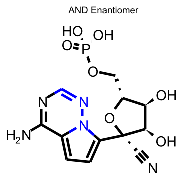 [*]:[cH]:n:n(:[*]):[*]	-1.29	0 out of 4
FCFP_10	-332197802	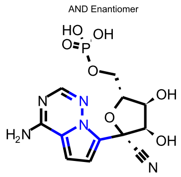 [*][c]1:[*]:[*]:[c](:[*]):n:1:n:[*]	-0.507	0 out of 1

FCFP_10	713358128	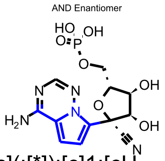<br>[*][c](:[*]):[c]1:[cH<br>]:[cH]:[c]([*]):n:1:<br>[*]	-0.307	8 out of 17

Patuletin

TOPKAT_Ocular_Irritancy_None_vs_Irritant

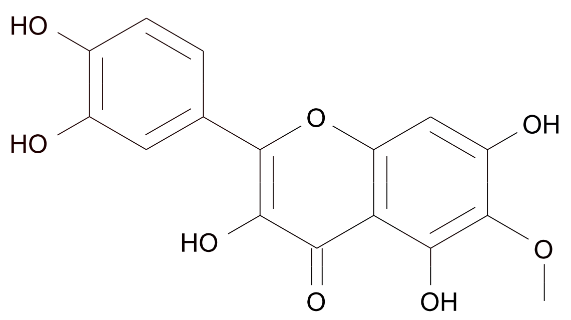

$C_{16}H_{12}O_8$
Molecular Weight: 332.26168
ALogP: 1.614
Rotatable Bonds: 2
Acceptors: 8
Donors: 5

Model Prediction

Prediction: Irritant
Probability: 1
Enrichment: 1.18
Bayesian Score: 1.34
Mahalanobis Distance: 9.63
Mahalanobis Distance p-value: 0.203

Prediction: Positive if the Bayesian score is above the estimated best cutoff value from minimizing the false positive and false negative rate.
Probability: The esimated probability that the sample is in the positive category. This assumes that the Bayesian score follows a normal distribution and is different from the prediction using a cutoff.
Enrichment: An estimate of enrichment, that is, the increased likelihood (versus random) of this sample being in the category.
Bayesian Score: The standard Laplacian-modified Bayesian score.
Mahalanobis Distance: The Mahalanobis distance (MD) is the distance to the center of the training data. The larger the MD, the less trustworthy the prediction.
Mahalanobis Distance p-value: The p-value gives the fraction of training data with an MD greater than or equal to the one for the given sample, assuming normally distributed data. The smaller the p-value, the less trustworthy the prediciton. For highly non-normal X properties (e.g., fingerprints), the MD p-value is wildly inaccurate.

Structural Similar Compounds

Name	ANTHRAQUINONE; 1;5-DIAMINO-4;8-DIHYDROXY-3-(p-METHOXYPHENYL)-	2-Naphthalenesulfonic acid; 5;6'-iminobis(1-hydroxy-	ANTHRAQUINONE; 1;4;5;8-TETRAHYDROXY-
Structure	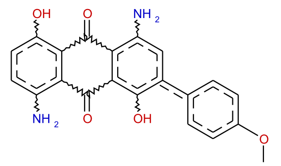	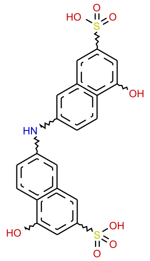	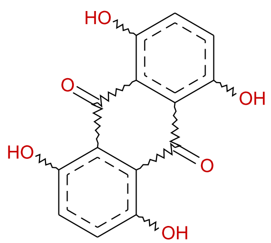
Actual Endpoint	Irritant	Irritant	Irritant
Predicted Endpoint	Irritant	Irritant	Irritant
Distance	0.727	0.739	0.750
Reference	28ZPAK 245;72	Prehled Prumyslove Toxikologie; Organicke Latky; Marhold; J. pp 1065;86	28ZPAK-;104;72

Model Applicability

Unknown features are fingerprint features in the query molecule, but not found or appearing too infrequently in the training set.

1. All properties and OPS components are within expected ranges.
2. Unknown FCFP_2 feature: -1678245750: [*]OC(=C([*])[*])[c](:[*]):[*]
3. Unknown FCFP_2 feature: -1305924292: [*]C(=C(O)C(=[*])[*])[*]

Feature Contribution

Top features for positive contribution				
Fingerprint	Bit/Smiles	Feature Structure	Score	Irritant in training set

FCFP_12	-548632217	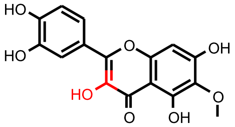 [*]C(=[*])O	0.177	59 out of 61
FCFP_12	-1099193755	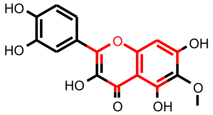 [*]C1=[*]C(=[*])[c]2:[c]([*]):[*]:[c]([*]):[cH]:[c]:2O1	0.175	5 out of 5
FCFP_12	-204034463	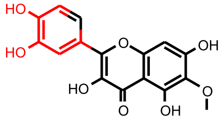 [*][c]1:[*]:[cH]:[c](O):[c](O):[cH]:1	0.175	5 out of 5
**Top Features for negative contribution**				
**Fingerprint**	**Bit/Smiles**	**Feature Structure**	**Score**	**Irritant in training set**
FCFP_12	1673930087	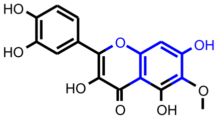 [*]O[c]1:[cH]:[c](O):[c]([*]):[*]:[c]:1[*]	-0.218	5 out of 8
FCFP_12	0	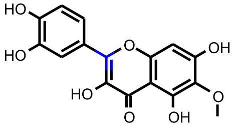 [*]C(=[*])[*]	0	1184 out of 1397

FCFP_12	203677720	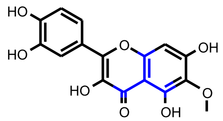 [*]C(=[*])[c](:[c](:[c]([*]):[*]):[c]([*]):[*]	0	319 out of 382

Remdesivir

TOPKAT_Ocular_Irritancy_None_vs_Irritant

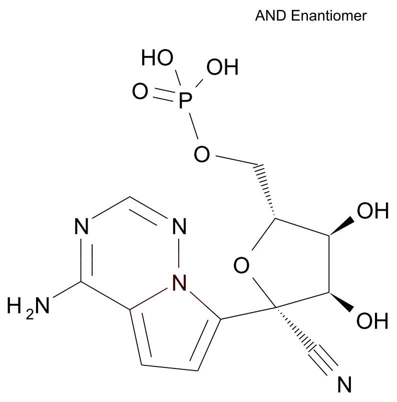

$C_{12}H_{14}N_5O_7P$

Molecular Weight: 371.24262

ALogP: -1.502

Rotatable Bonds: 4

Acceptors: 11

Donors: 5

Structural Similar Compounds

Name	1;3;6-NAPHTHALENE TRISULFONIC ACID;7-AMINO-	Methanol; (s-triazine-2;4;6-triyltrinitrilo)hexa-	2;2'-Biphenyldisulfonic acid; 4;4'-diamino-
Structure	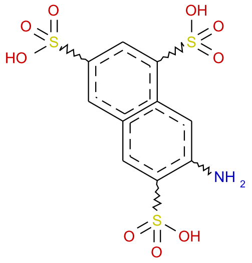	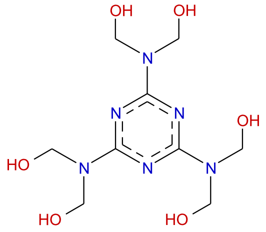	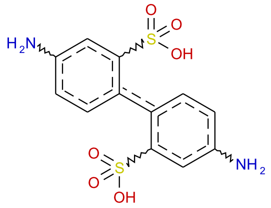
Actual Endpoint	Irritant	Irritant	Irritant
Predicted Endpoint	Irritant	Irritant	Irritant
Distance	0.766	0.795	0.859
Reference	28ZPAK-;190;72	Prehled Prumyslove Toxikologie; Organicke Latky; Marhold; J. -;876;86	Prehled Prumyslove Toxikologie; Organicke Latky; Marhold; J. pp 1061;86

Model Prediction

Prediction: Irritant

Probability: 1

Enrichment: 1.18

Bayesian Score: 1.33

Mahalanobis Distance: 10.7

Mahalanobis Distance p-value: 0.0147

Prediction: Positive if the Bayesian score is above the estimated best cutoff value from minimizing the false positive and false negative rate.
Probability: The esimated probability that the sample is in the positive category. This assumes that the Bayesian score follows a normal distribution and is different from the prediction using a cutoff.
Enrichment: An estimate of enrichment, that is, the increased likelihood (versus random) of this sample being in the category.
Bayesian Score: The standard Laplacian-modified Bayesian score.
Mahalanobis Distance: The Mahalanobis distance (MD) is the distance to the center of the training data. The larger the MD, the less trustworthy the prediction.
Mahalanobis Distance p-value: The p-value gives the fraction of training data with an MD greater than or equal to the one for the given sample, assuming normally distributed data. The smaller the p-value, the less trustworthy the prediciton. For highly non-normal X properties (e.g., fingerprints), the MD p-value is wildly inaccurate.

Model Applicability

Unknown features are fingerprint features in the query molecule, but not found or appearing too infrequently in the training set.

1. All properties and OPS components are within expected ranges.
2. Unknown FCFP_2 feature: 472180098: [*]OP(=O)(O)O
3. Unknown FCFP_2 feature: -124685461: [*]:n:c:n:[*]
4. Unknown FCFP_2 feature: -1151884458: [*]:n:[c](N):[c](:[*]):[*]

Feature Contribution

Top features for positive contribution				
Fingerprint	Bit/Smiles	Feature Structure	Score	Irritant in training set

FCFP_12	1747237384	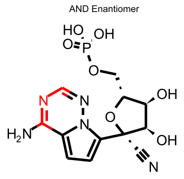 [*][c](:[*]):n:[cH]:[*]	0.208	44 out of 44
FCFP_12	178336375	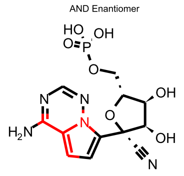 [*][c](:[*]):[c]1:[cH]:[*]:[*]:n:1:[*]	0.202	19 out of 19
FCFP_12	713358128	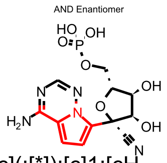 [*][c](:[*]):[c]1:[cH]:[cH]:[c]([*]):n:1:[*]	0.2	17 out of 17
**Top Features for negative contribution**				
**Fingerprint**	**Bit/Smiles**	**Feature Structure**	**Score**	**Irritant in training set**
FCFP_12	-836603894	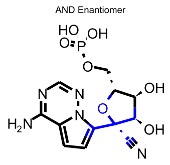 [*][C@@H]1[*][*]O[C@]1(C#[*])[c](:[*]):[*]	-0.592	0 out of 1
FCFP_12	-1277879912	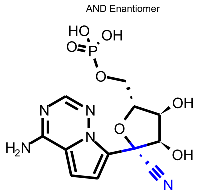 [*]C([*])([*])C#N	-0.0939	33 out of 45

FCFP_12	-1272768868	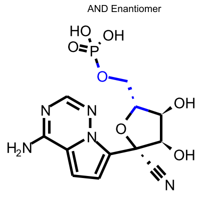 [*]OCC([*])[*]	0	396 out of 514

Patuletin

TOPKAT_Rat_Female_FDA_None_vs_Carcinogen

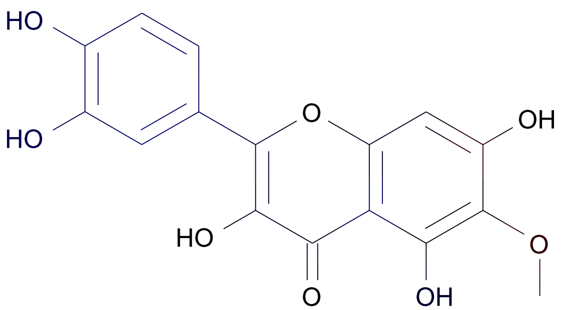

$C_{16}H_{12}O_8$

Molecular Weight: 332.26168

ALogP: 1.614

Rotatable Bonds: 2

Acceptors: 8

Donors: 5

Model Prediction

Prediction: Non-Carcinogen

Probability: 0.228

Enrichment: 0.709

Bayesian Score: -4.4

Mahalanobis Distance: 11.8

Mahalanobis Distance p-value: 0.00851

Prediction: Positive if the Bayesian score is above the estimated best cutoff value from minimizing the false positive and false negative rate.
Probability: The esimated probability that the sample is in the positive category. This assumes that the Bayesian score follows a normal distribution and is different from the prediction using a cutoff.
Enrichment: An estimate of enrichment, that is, the increased likelihood (versus random) of this sample being in the category.
Bayesian Score: The standard Laplacian-modified Bayesian score.
Mahalanobis Distance: The Mahalanobis distance (MD) is the distance to the center of the training data. The larger the MD, the less trustworthy the prediction.
Mahalanobis Distance p-value: The p-value gives the fraction of training data with an MD greater than or equal to the one for the given sample, assuming normally distributed data. The smaller the p-value, the less trustworthy the prediciton. For highly non-normal X properties (e.g., fingerprints), the MD p-value is wildly inaccurate.

Structural Similar Compounds

Name	Olsalazine	Carbidopa	Lodoxamide
Structure	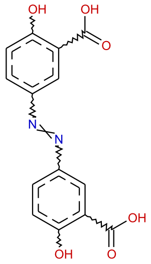	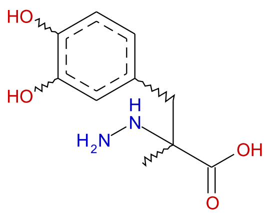	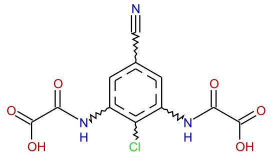
Actual Endpoint	Non-Carcinogen	Non-Carcinogen	Non-Carcinogen
Predicted Endpoint	Non-Carcinogen	Non-Carcinogen	Non-Carcinogen
Distance	0.695	0.749	0.754
Reference	US FDA (Centre for Drug Eval.& Res./Off. Testing & Res.) Sept. 1997	US FDA (Centre for Drug Eval.& Res./Off. Testing & Res.) Sept. 1997	US FDA (Centre for Drug Eval.& Res./Off. Testing & Res.) Sept. 1997

Model Applicability

Unknown features are fingerprint features in the query molecule, but not found or appearing too infrequently in the training set.

1. All properties and OPS components are within expected ranges.

Feature Contribution

Top features for positive contribution

Fingerprint	Bit/Smiles	Feature Structure	Score	Carcinogen in training set
ECFP_12	2052151141	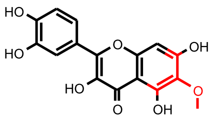 [*][c](:[*]):[c](OC):[c]([*]):[*]	0.668	4 out of 5

ECFP_12	-1531301414	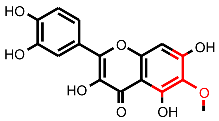<br>[*]O[c](:[c]([*]):[*]<br>):[c]([*]):[*]	0.454	5 out of 9
ECFP_12	683445015	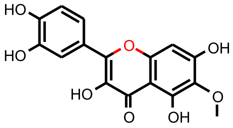<br>[*]O[*]	0.294	28 out of 66
**Top Features for negative contribution**				
**Fingerprint**	**Bit/Smiles**	**Feature Structure**	**Score**	**Carcinogen in training set**
ECFP_12	-1660913849	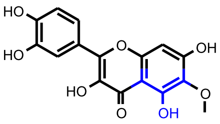<br>[*][c](:[*]):[c](O):[<br>c]([*]):[*]	-0.941	0 out of 5
ECFP_12	-181568884	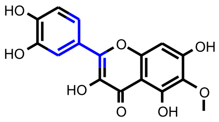<br>[*]C(=[*])[c](:[cH]:[<br>*]):[cH]:[*]	-0.505	3 out of 18
ECFP_12	1310213750	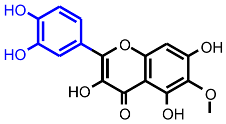<br>[*][c]1:[cH]:[cH]:[c]<br>(O):[c](O):[cH]:1	-0.485	0 out of 2

Remdesivir

TOPKAT_Rat_Female_FDA_None_vs_Carcinogen

AND Enantiomer

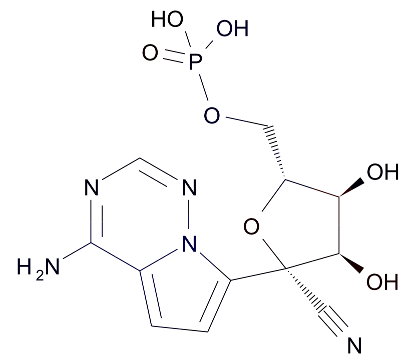

$C_{12}H_{14}N_5O_7P$
Molecular Weight: 371.24262
ALogP: -1.502
Rotatable Bonds: 4
Acceptors: 11
Donors: 5

Model Prediction

Prediction: Non-Carcinogen
Probability: 0.243
Enrichment: 0.756
Bayesian Score: -3.24
Mahalanobis Distance: 13.8
Mahalanobis Distance p-value: 5.04e-006

Prediction: Positive if the Bayesian score is above the estimated best cutoff value from minimizing the false positive and false negative rate.
Probability: The esimated probability that the sample is in the positive category. This assumes that the Bayesian score follows a normal distribution and is different from the prediction using a cutoff.
Enrichment: An estimate of enrichment, that is, the increased likelihood (versus random) of this sample being in the category.
Bayesian Score: The standard Laplacian-modified Bayesian score.
Mahalanobis Distance: The Mahalanobis distance (MD) is the distance to the center of the training data. The larger the MD, the less trustworthy the prediction.
Mahalanobis Distance p-value: The p-value gives the fraction of training data with an MD greater than or equal to the one for the given sample, assuming normally distributed data. The smaller the p-value, the less trustworthy the prediciton. For highly non-normal X properties (e.g., fingerprints), the MD p-value is wildly inaccurate.

Structural Similar Compounds

Name	Streptozocin	Tetracycline	Famotidine
Structure	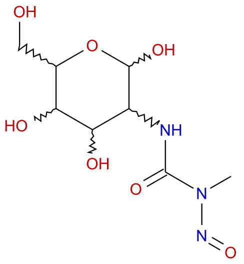	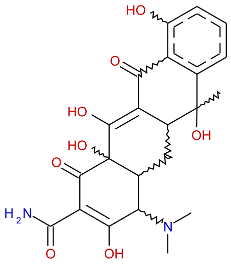	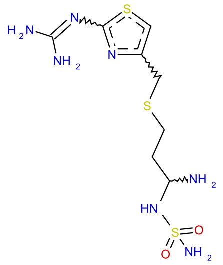
Actual Endpoint	Carcinogen	Non-Carcinogen	Non-Carcinogen
Predicted Endpoint	Carcinogen	Non-Carcinogen	Non-Carcinogen
Distance	0.810	0.858	0.861
Reference	US FDA (Centre for Drug Eval.& Res./Off. Testing & Res.) Sept. 1997	US FDA (Centre for Drug Eval.& Res./Off. Testing & Res.) Sept. 1997	US FDA (Centre for Drug Eval.& Res./Off. Testing & Res.) Sept. 1997

Model Applicability

Unknown features are fingerprint features in the query molecule, but not found or appearing too infrequently in the training set.

1. All properties and OPS components are within expected ranges.
2. Unknown ECFP_2 feature: 1126642748: [*]OP(=O)(O)O
3. Unknown ECFP_2 feature: -1250439909: [*]COP(=[*])([*])[*]
4. Unknown ECFP_2 feature: 1258791451: [*][C@@H]1[*][*]O[C@]1(C#[*])[c](:[*]):[*]
5. Unknown ECFP_2 feature: -1507082173: [*][c]1:[*]:[*]:[c](:[*]):n:1:n:[*]
6. Unknown ECFP_2 feature: -66263742: [*]C([*])([*])[c]1:n(:[*]):[*]:[*]:c:1

Feature Contribution

Top features for positive contribution				
Fingerprint	Bit/Smiles	Feature Structure	Score	Carcinogen in training set

ECFP_12	-553149446	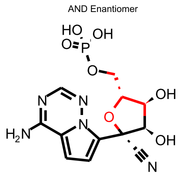 [*]C[C@H]1O[*][*][C@@H]1[*]	0.575	3 out of 4
ECFP_12	-1114776580	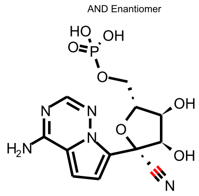 [*]C#[*]	0.461	10 out of 19
ECFP_12	-521596699	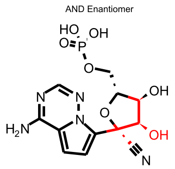 [*][C@@H]1[*][*]C([*])([*])[C@@H]1O	0.445	3 out of 5

Top Features for negative contribution

Fingerprint	Bit/Smiles	Feature Structure	Score	Carcinogen in training set
ECFP_12	-1687549011	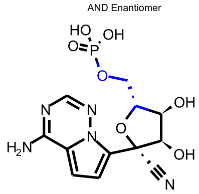 [*]OCC([*])[*]	-0.661	0 out of 3
ECFP_12	2024329577	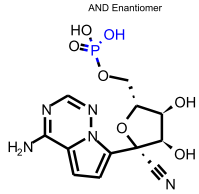 [*]P(=[*])([*])O	-0.661	0 out of 3

ECFP_12	-1734834311	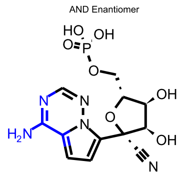<br>[*]:n:[c](N):[c](:[*]<br>):[*]	-0.56	1 out of 8

Patuletin

TOPKAT_Rat_Male_FDA_None_vs_Carcinogen

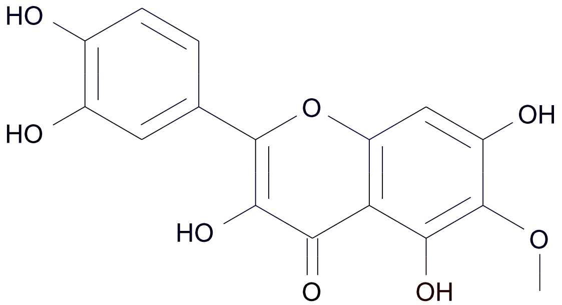

$C_{16}H_{12}O_8$
Molecular Weight: 332.26168
ALogP: 1.614
Rotatable Bonds: 2
Acceptors: 8
Donors: 5

Structural Similar Compounds

Name	Olsalazine	Carbidopa	Lodoxamide
Structure	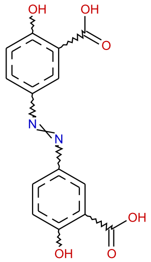	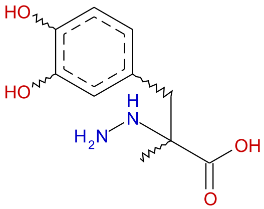	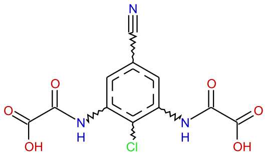
Actual Endpoint	Non-Carcinogen	Non-Carcinogen	Non-Carcinogen
Predicted Endpoint	Carcinogen	Non-Carcinogen	Non-Carcinogen
Distance	0.651	0.715	0.726
Reference	US FDA (Centre for Drug Eval.& Res./Off. Testing & Res.) Sept. 1997	US FDA (Centre for Drug Eval.& Res./Off. Testing & Res.) Sept. 1997	US FDA (Centre for Drug Eval.& Res./Off. Testing & Res.) Sept. 1997

Model Prediction

Prediction: Non-Carcinogen
Probability: 0.282
Enrichment: 0.845
Bayesian Score: -2.87
Mahalanobis Distance: 12.1
Mahalanobis Distance p-value: 0.0207

Prediction: Positive if the Bayesian score is above the estimated best cutoff value from minimizing the false positive and false negative rate.
Probability: The esimated probability that the sample is in the positive category. This assumes that the Bayesian score follows a normal distribution and is different from the prediction using a cutoff.
Enrichment: An estimate of enrichment, that is, the increased likelihood (versus random) of this sample being in the category.
Bayesian Score: The standard Laplacian-modified Bayesian score.
Mahalanobis Distance: The Mahalanobis distance (MD) is the distance to the center of the training data. The larger the MD, the less trustworthy the prediction.
Mahalanobis Distance p-value: The p-value gives the fraction of training data with an MD greater than or equal to the one for the given sample, assuming normally distributed data. The smaller the p-value, the less trustworthy the prediciton. For highly non-normal X properties (e.g., fingerprints), the MD p-value is wildly inaccurate.

Model Applicability

Unknown features are fingerprint features in the query molecule, but not found or appearing too infrequently in the training set.

1. All properties and OPS components are within expected ranges.

Feature Contribution

Top features for positive contribution

Fingerprint	Bit/Smiles	Feature Structure	Score	Carcinogen in training set
SCFP_6	-1547271378	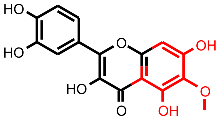 [*][c]1:[*]:[cH]:[c](O):[c](OC):[c]:1O	0.603	2 out of 2

SCFP_6	392579710	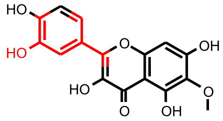 [*]C(=[*])[c]1:[cH]:[*]:[c]([*]):[c](O):[cH]:1	0.425	2 out of 3
SCFP_6	1157879834	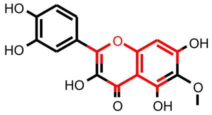 [*]C1=[*]C(=[*])[c]2: [c]([*]):[*]:[c]([*]):[cH]:[c]:2O1	0.198	1 out of 2

Top Features for negative contribution

Fingerprint	Bit/Smiles	Feature Structure	Score	Carcinogen in training set
SCFP_6	2116304939	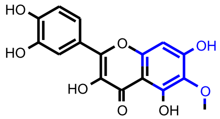 [*]O[c]1:[c]([*]):[*] :[c]([*]):[cH]:[c]:1 O	-0.825	0 out of 4
SCFP_6	-700178387	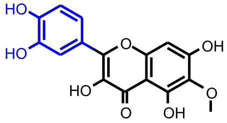 [*][c]1:[cH]:[cH]:[c] (O):[c](O):[cH]:1	-0.496	0 out of 2
SCFP_6	616636418	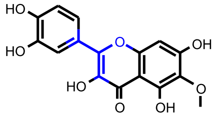 [*]OC(=C([*])[*])[c](:[*]):[*]	-0.278	0 out of 1

Remdesivir

TOPKAT_Rat_Male_FDA_None_vs_Carcinogen

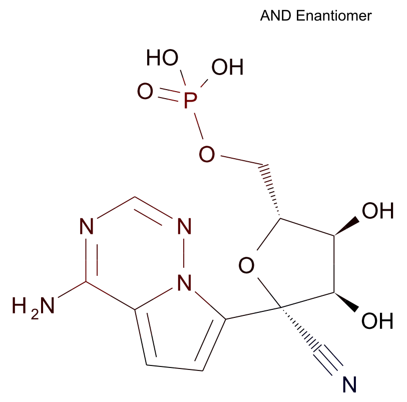

$C_{12}H_{14}N_5O_7P$

Molecular Weight: 371.24262

ALogP: -1.502

Rotatable Bonds: 4

Acceptors: 11

Donors: 5

Structural Similar Compounds

Name	Streptozocin	Famotidine	Tetracycline
Structure	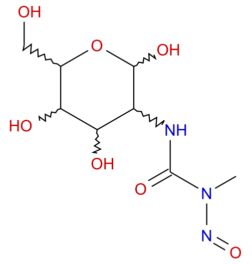	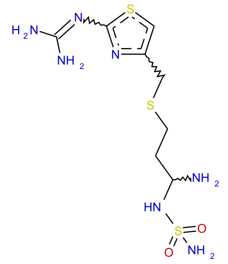	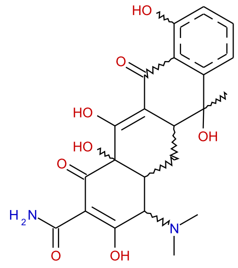
Actual Endpoint	Carcinogen	Non-Carcinogen	Non-Carcinogen
Predicted Endpoint	Carcinogen	Non-Carcinogen	Non-Carcinogen
Distance	0.789	0.850	0.856
Reference	US FDA (Centre for Drug Eval.& Res./Off. Testing & Res.) Sept. 1997	US FDA (Centre for Drug Eval.& Res./Off. Testing & Res.) Sept. 1997	US FDA (Centre for Drug Eval.& Res./Off. Testing & Res.) Sept. 1997

Model Prediction

Prediction: Carcinogen

Probability: 0.481

Enrichment: 1.44

Bayesian Score: 3.82

Mahalanobis Distance: 14.1

Mahalanobis Distance p-value: 3.32e-005

Prediction: Positive if the Bayesian score is above the estimated best cutoff value from minimizing the false positive and false negative rate.
Probability: The esimated probability that the sample is in the positive category. This assumes that the Bayesian score follows a normal distribution and is different from the prediction using a cutoff.
Enrichment: An estimate of enrichment, that is, the increased likelihood (versus random) of this sample being in the category.
Bayesian Score: The standard Laplacian-modified Bayesian score.
Mahalanobis Distance: The Mahalanobis distance (MD) is the distance to the center of the training data. The larger the MD, the less trustworthy the prediction.
Mahalanobis Distance p-value: The p-value gives the fraction of training data with an MD greater than or equal to the one for the given sample, assuming normally distributed data. The smaller the p-value, the less trustworthy the prediciton. For highly non-normal X properties (e.g., fingerprints), the MD p-value is wildly inaccurate.

Model Applicability

Unknown features are fingerprint features in the query molecule, but not found or appearing too infrequently in the training set.

1. All properties and OPS components are within expected ranges.

Feature Contribution

Top features for positive contribution

Fingerprint	Bit/Smiles	Feature Structure	Score	Carcinogen in training set
SCFP_6	-1029620989	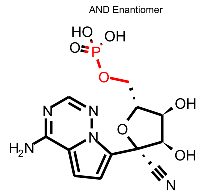 [*]COP(=[*])([*])[*]	0.712	3 out of 3

SCFP_6	1245795878	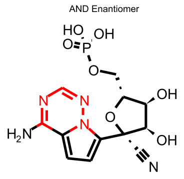 [*][c]1:[*]:n(:[*]):n :[cH]:n:1	0.603	2 out of 2
SCFP_6	149212520	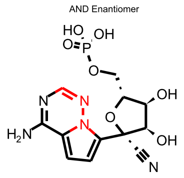 [*]:[cH]:n:n(:[*]):[*]	0.543	9 out of 15
**Top Features for negative contribution**				
**Fingerprint**	**Bit/Smiles**	**Feature Structure**	**Score**	**Carcinogen in training set**
SCFP_6	-1019297400	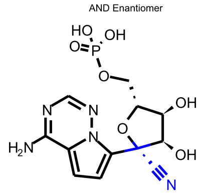 [*]C([*])([*])C#N	-0.674	0 out of 3
SCFP_6	194135988	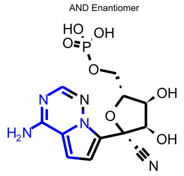 N[c]1:n:[cH]:[*]:n2:[*]:[*]:[cH]:[c]:1:2	-0.278	0 out of 1
SCFP_6	-424515134	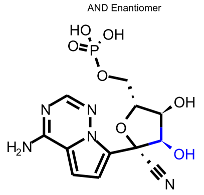 [*]C([*])O	-0.157	30 out of 110

Remdesivir

TOPKAT_Rat_Male_FDA_Single_vs_Multiple

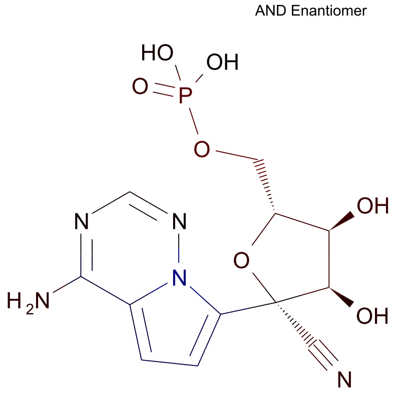

$C_{12}H_{14}N_5O_7P$

Molecular Weight: 371.24262

ALogP: -1.502

Rotatable Bonds: 4

Acceptors: 11

Donors: 5

Structural Similar Compounds

Name	Streptozocin	Minocycline	Ribavirin
Structure	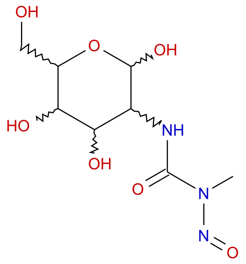	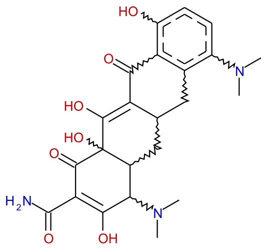	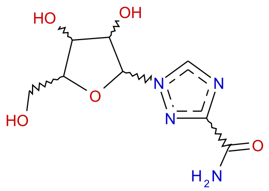
Actual Endpoint	Multiple-Carcinogen	Single-Carcinogen	Multiple-Carcinogen
Predicted Endpoint	Multiple-Carcinogen	Single-Carcinogen	Multiple-Carcinogen
Distance	0.817	0.908	0.929
Reference	US FDA (Centre for Drug Eval.& Res./Off. Testing & Res.) Sept. 1997	US FDA (Centre for Drug Eval.& Res./Off. Testing & Res.) Sept. 1997	US FDA (Centre for Drug Eval.& Res./Off. Testing & Res.) Sept. 1997

Model Prediction

Prediction: Multiple-Carcinogen

Probability: 0.556

Enrichment: 1.34

Bayesian Score: 3.52

Mahalanobis Distance: 14

Mahalanobis Distance p-value: 8.72e-005

Prediction: Positive if the Bayesian score is above the estimated best cutoff value from minimizing the false positive and false negative rate.
Probability: The esimated probability that the sample is in the positive category. This assumes that the Bayesian score follows a normal distribution and is different from the prediction using a cutoff.
Enrichment: An estimate of enrichment, that is, the increased likelihood (versus random) of this sample being in the category.
Bayesian Score: The standard Laplacian-modified Bayesian score.
Mahalanobis Distance: The Mahalanobis distance (MD) is the distance to the center of the training data. The larger the MD, the less trustworthy the prediction.
Mahalanobis Distance p-value: The p-value gives the fraction of training data with an MD greater than or equal to the one for the given sample, assuming normally distributed data. The smaller the p-value, the less trustworthy the prediciton. For highly non-normal X properties (e.g., fingerprints), the MD p-value is wildly inaccurate.

Model Applicability

Unknown features are fingerprint features in the query molecule, but not found or appearing too infrequently in the training set.

1. Num_H_Acceptors out of range. Value: 11. Training min, max, mean, SD: 0, 9, 3.8906, 2.196.

Feature Contribution

Top features for positive contribution

Fingerprint	Bit/Smiles	Feature Structure	Score	Multiple-Carcinogen in training set
SCFP_8	-1029620989	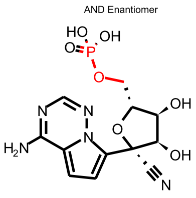 [*]COP(=[*])([*])[*]	0.649	3 out of 3

SCFP_8	2	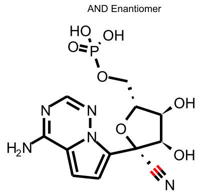<br>[*]C#[*]	0.584	6 out of 8
SCFP_8	-1486266146	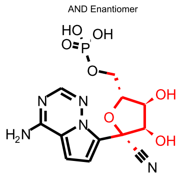<br>[*]C[C@H]1OC([*])([*]<br>)[C@H](O)[C@@H]1O	0.553	2 out of 2
**Top Features for negative contribution**				
**Fingerprint**	**Bit/Smiles**	**Feature Structure**	**Score**	**Multiple-Carcinogen in training set**
SCFP_8	-1381862798	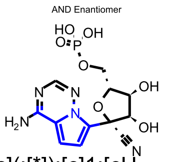<br>[*][c](:[*]):[c]1:[cH<br>]:[cH]:[c]([*]):n:1:<br>[*]	-0.572	1 out of 7
SCFP_8	1245795878	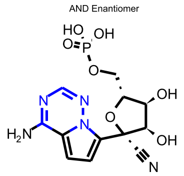<br>[*][c]1:[*]:n(:[*]):n<br>:[cH]:n:1	-0.546	0 out of 2
SCFP_8	-1375522316	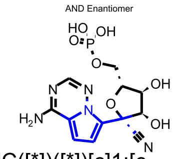<br>[*]C([*])([*])[c]1:[c<br>H]:[cH]:[c](:[*]):n:<br>1:[*]	-0.546	0 out of 2

Remdesivir

TOPKAT_Skin_Irritancy_Mild_vs_Moderate_Severe

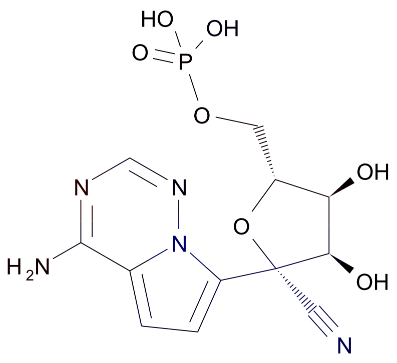

$C_{12}H_{14}N_5O_7P$
Molecular Weight: 371.24262
ALogP: -1.502
Rotatable Bonds: 4
Acceptors: 11
Donors: 5

Model Prediction

Prediction: Mild
Probability: 0.0911
Enrichment: 0.247
Bayesian Score: -8.73
Mahalanobis Distance: 13.5
Mahalanobis Distance p-value: 1.21e-009

Prediction: Positive if the Bayesian score is above the estimated best cutoff value from minimizing the false positive and false negative rate.
Probability: The esimated probability that the sample is in the positive category. This assumes that the Bayesian score follows a normal distribution and is different from the prediction using a cutoff.
Enrichment: An estimate of enrichment, that is, the increased likelihood (versus random) of this sample being in the category.
Bayesian Score: The standard Laplacian-modified Bayesian score.
Mahalanobis Distance: The Mahalanobis distance (MD) is the distance to the center of the training data. The larger the MD, the less trustworthy the prediction.
Mahalanobis Distance p-value: The p-value gives the fraction of training data with an MD greater than or equal to the one for the given sample, assuming normally distributed data. The smaller the p-value, the less trustworthy the prediciton. For highly non-normal X properties (e.g., fingerprints), the MD p-value is wildly inaccurate.

Structural Similar Compounds

Name	1,3,6-Naphthalenetrisulfonic acid, 7-amino-	2,7-Anthracenedisulfonic acid, 9,10-dihydro-4,5-diamino-9,10-dioxo- 1-hydroxy-, disodium salt	1,5-Naphthalenedisulfonic acid, 2-amino-
Structure	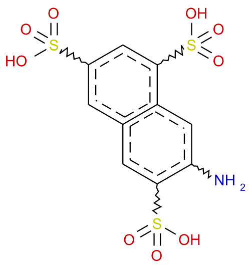	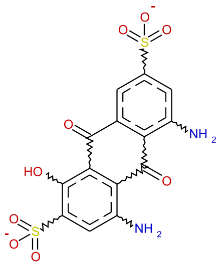	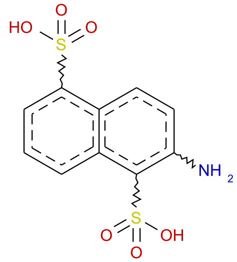
Actual Endpoint	Mild	Mild	Mild
Predicted Endpoint	Mild	Mild	Mild
Distance	0.759	1.033	1.137
Reference	85JCAE "Prehled Prumyslove Toxikologie; Organicke Latky," Marhold, J., Prague , Czechoslovakia, Avicenum, 1986 Volume(issue)/page/year: -,1058,1986	28ZPAK "Sbornik Vysledku Toxixologickeho Vysetreni Latek A Pripravku," Marhold d, J.V., Institut Pro Vychovu Vedoucicn Pracovniku Chemickeho Prumyclu Praha, Cz echoslovakia, 1972 Volume(issue)/page/year: -,239,1	85JCAE "Prehled Prumyslove Toxikologie; Organicke Latky," Marhold, J., Prague , Czechoslovakia, Avicenum, 1986 Volume(issue)/page/year: -,1058,1986

Model Applicability

Unknown features are fingerprint features in the query molecule, but not found or appearing too infrequently in the training set.

1. All properties and OPS components are within expected ranges.
2. Unknown FCFP_2 feature: 472180098: [*]OP(=O)(O)O
3. Unknown FCFP_2 feature: -332197802: [*][c]1:[*]:[*]:[c](:[*]):n:1:n:[*]

Feature Contribution

Top features for positive contribution

Fingerprint	Bit/Smiles	Feature Structure	Score	Moderate_Severe in training set

FCFP_12	-1151884458	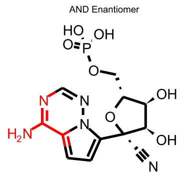 [*]:n:[c](N):[c](:[*]):[*]	0.385	1 out of 1
FCFP_12	76292238	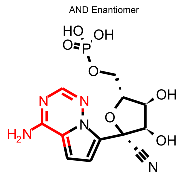 [*]:[c]1:[*]:n:[cH]:n :[c]:1N	0.385	1 out of 1
FCFP_12	-124685461	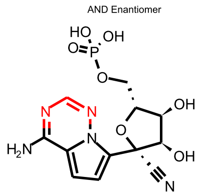 [*]:n:[cH]:n:[*]	0.206	2 out of 4

Top Features for negative contribution

Fingerprint	Bit/Smiles	Feature Structure	Score	Moderate_Severe in training set
FCFP_12	4427049	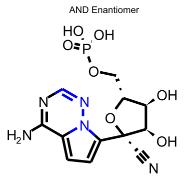 [*]:[cH]:n:n(:[*]):[*]	-0.893	0 out of 4
FCFP_12	-1277879912	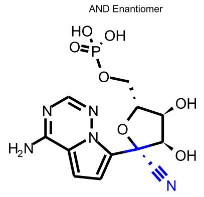 [*]C([*])([*])C#N	-0.548	5 out of 26

FCFP_12	-836603894	AND Enantiomer<br>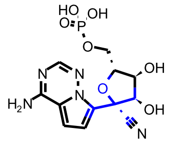<br>[*][C@@H]1[*][*]O[C@]1(C#[*])[c](:[*]):[*]	-0.543	0 out of 2

Patuletin

TOPKAT_Skin_Irritancy_None_vs_Irritant

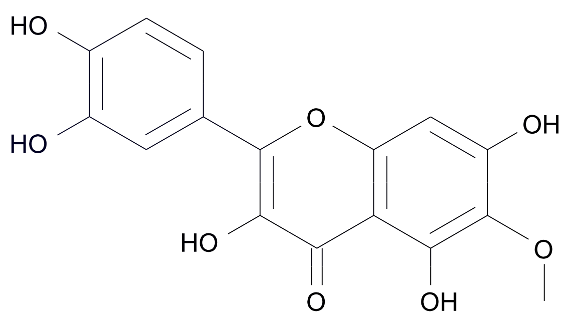

$C_{16}H_{12}O_8$
Molecular Weight: 332.26168
ALogP: 1.614
Rotatable Bonds: 2
Acceptors: 8
Donors: 5

Structural Similar Compounds

Name	Anthraquinone, 2-bromo-1,8-diamino-4,5-dihydroxy-	Anthraquinone, 1,4,5,8-tetrahydroxy-	Anthraquinone, 1,5-diamino-4,8-dihydroxy-
Structure	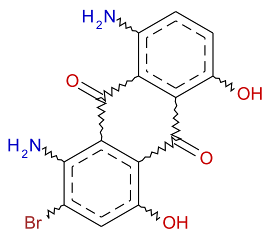	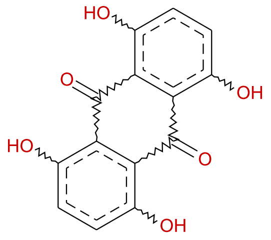	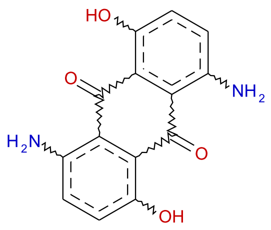
Actual Endpoint	Irritant	Irritant	Non-Irritant
Predicted Endpoint	Non-Irritant	Non-Irritant	Non-Irritant
Distance	0.756	0.779	0.786
Reference	28ZPAK "Sbornik Vysledku Toxixologickeho Vysetreni Latek A Pripravku," Marhol d, J.V., Institut Pro Vychovu Vedoucicn Pracovniku Chemickeho Prumyclu Praha, Cz echoslovakia, 1972 Volume(issue)/page/year: -,244,1	85JCAE "Prehled Prumyslove Toxikologie; Organicke Latky," Marhold, J., Prague , Czechoslovakia, Avicenum, 1986 Volume(issue)/page/year: -,655,1986	28ZPAK -,103,72

Model Prediction

Prediction: Non-Irritant
Probability: 0.958
Enrichment: 1.04
Bayesian Score: -1.64
Mahalanobis Distance: 13.8
Mahalanobis Distance p-value: 3.24e-010

Prediction: Positive if the Bayesian score is above the estimated best cutoff value from minimizing the false positive and false negative rate.
Probability: The esimated probability that the sample is in the positive category. This assumes that the Bayesian score follows a normal distribution and is different from the prediction using a cutoff.
Enrichment: An estimate of enrichment, that is, the increased likelihood (versus random) of this sample being in the category.
Bayesian Score: The standard Laplacian-modified Bayesian score.
Mahalanobis Distance: The Mahalanobis distance (MD) is the distance to the center of the training data. The larger the MD, the less trustworthy the prediction.
Mahalanobis Distance p-value: The p-value gives the fraction of training data with an MD greater than or equal to the one for the given sample, assuming normally distributed data. The smaller the p-value, the less trustworthy the prediciton. For highly non-normal X properties (e.g., fingerprints), the MD p-value is wildly inaccurate.

Model Applicability

Unknown features are fingerprint features in the query molecule, but not found or appearing too infrequently in the training set.

1. All properties and OPS components are within expected ranges.

Feature Contribution

Top features for positive contribution

Fingerprint	Bit/Smiles	Feature Structure	Score	Irritant in training set

FCFP_12	523826990	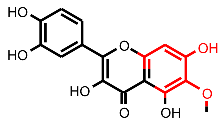<br>[*]O[c]1:[c]([*]):[*]<br>:[c]([*]):[cH]:[c]:1<br>O	0.0756	6 out of 6
FCFP_12	-1678245750	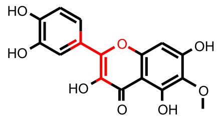<br>[*]OC(=C([*])[*])[c](<br>:[*]):[*]	0.0583	2 out of 2
**Top Features for negative contribution**				
**Fingerprint**	**Bit/Smiles**	**Feature Structure**	**Score**	**Irritant in training set**
FCFP_12	-204034463	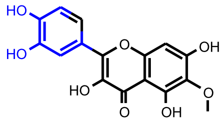<br>[*][c]1:[*]:[cH]:[c](<br>O):[c](O):[cH]:1	-0.222	2 out of 3
FCFP_12	949015626	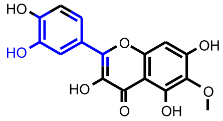<br>[*]C(=[*])[c]1:[cH]:[<br>*]:[c]([*]):[c](O):[<br>cH]:1	-0.222	2 out of 3
FCFP_12	-548632217	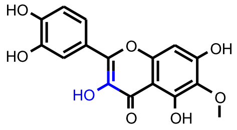<br>[*]C(=[*])O	-0.128	49 out of 61

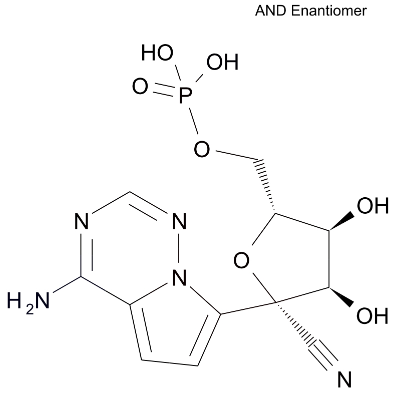

$C_{12}H_{14}N_5O_7P$

Molecular Weight: 371.24262

ALogP: -1.502

Rotatable Bonds: 4

Acceptors: 11

Donors: 5

Model Prediction

Prediction: Irritant

Probability: 0.976

Enrichment: 1.06

Bayesian Score: -0.492

Mahalanobis Distance: 13.2

Mahalanobis Distance p-value: 3.18e-008

Prediction: Positive if the Bayesian score is above the estimated best cutoff value from minimizing the false positive and false negative rate.
Probability: The esimated probability that the sample is in the positive category. This assumes that the Bayesian score follows a normal distribution and is different from the prediction using a cutoff.
Enrichment: An estimate of enrichment, that is, the increased likelihood (versus random) of this sample being in the category.
Bayesian Score: The standard Laplacian-modified Bayesian score.
Mahalanobis Distance: The Mahalanobis distance (MD) is the distance to the center of the training data. The larger the MD, the less trustworthy the prediction.
Mahalanobis Distance p-value: The p-value gives the fraction of training data with an MD greater than or equal to the one for the given sample, assuming normally distributed data. The smaller the p-value, the less trustworthy the prediciton. For highly non-normal X properties (e.g., fingerprints), the MD p-value is wildly inaccurate.

Structural Similar Compounds

Name	1,3,6-Naphthalenetrisulfonic acid, 7-amino-	2,2'-Benzidine disulfonic acid	2,7-Anthracenedisulfonic acid, 9,10-dihydro-4,5-diamino-9,10-dioxo- 1-hydroxy-, disodium salt
Structure	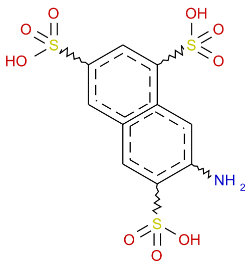	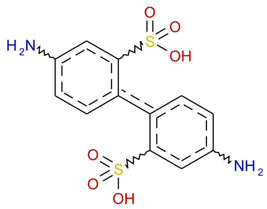	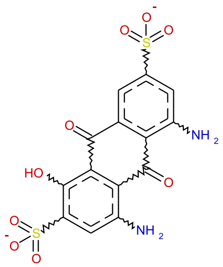
Actual Endpoint	Irritant	Non-Irritant	Irritant
Predicted Endpoint	Non-Irritant	Non-Irritant	Non-Irritant
Distance	0.755	0.896	1.025
Reference	85JCAE "Prehled Prumyslove Toxikologie; Organicke Latky," Marhold, J., Prague , Czechoslovakia, Avicenum, 1986 Volume(issue)/page/year: -,1058,1986	28ZPAK -,191,72	28ZPAK "Sbornik Vysledku Toxixologickeho Vysetreni Latek A Pripravku," Marhol d, J.V., Institut Pro Vychovu Vedoucicn Pracovniku Chemickeho Prumyclu Praha, Cz echoslovakia, 1972 Volume(issue)/page/year: -,239,1

Model Applicability

Unknown features are fingerprint features in the query molecule, but not found or appearing too infrequently in the training set.

1. All properties and OPS components are within expected ranges.
2. Unknown FCFP_2 feature: 472180098: [*]OP(=O)(O)O
3. Unknown FCFP_2 feature: -332197802: [*][c]1:[*]:[*]:[c](:[*]):n:1:n:[*]

Feature Contribution

Top features for positive contribution				
Fingerprint	Bit/Smiles	Feature Structure	Score	Irritant in training set

FCFP_12	654335567	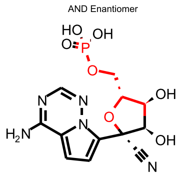 [*][C@H]1[*][*]O[C@@H]1COP(=[*])([*])[*]	0.0856	29 out of 29
FCFP_12	-1539132615	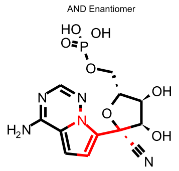 [*]C([*])([*])[c]1:[cH]:[*]:[*]:n:1:[*]	0.0795	9 out of 9
FCFP_12	-1280036918	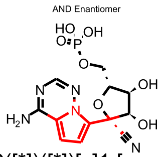 [*]C([*])([*])[c]1:[cH]:[cH]:[c](:[*]):n:1:[*]	0.0772	7 out of 7

Top Features for negative contribution

Fingerprint	Bit/Smiles	Feature Structure	Score	Irritant in training set
FCFP_12	1069584379	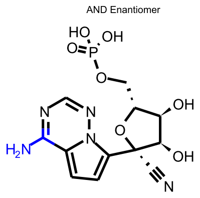 [*]:[c](:[*])N	-0.439	38 out of 65
FCFP_12	1618154665	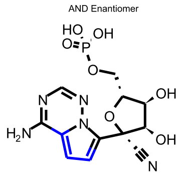 [*][c](:[*]):[cH]:[c]([*]):[*]	-0.0845	412 out of 490

FCFP_12	16	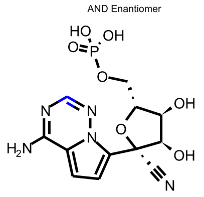 [*][c](:[*]):[*]	-0.0843	423 out of 503

Patuletin

TOPKAT_Carcinogenic_Potency_TD50_Mouse

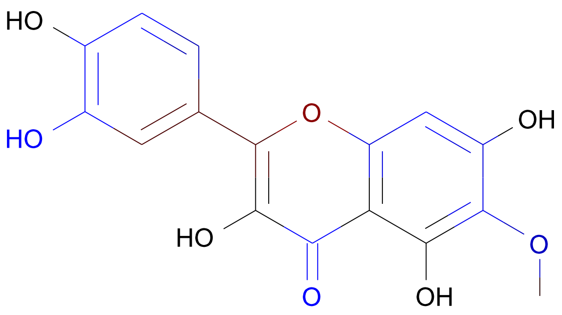

$C_{16}H_{12}O_8$

Molecular Weight: 332.26168

ALogP: 1.614

Rotatable Bonds: 2

Acceptors: 8

Donors: 5

Model Prediction

Prediction: 66.7

Unit: mg/kg_body_weight/day

Mahalanobis Distance: 13

Mahalanobis Distance p-value: 9.47e-008

Mahalanobis Distance: The Mahalanobis distance (MD) is a generalization of the Euclidean distance that accounts for correlations among the X properties. It is calculated as the distance to the center of the training data. The larger the MD, the less trustworthy the prediction.
Mahalanobis Distance p-value: The p-value gives the fraction of training data with an MD greater than or equal to the one for the given sample, assuming normally distributed data. The smaller the p-value, the less trustworthy the prediciton. For highly non-normal X properties (e.g., fingerprints), the MD p-value is wildly inaccurate.

Structural Similar Compounds

Name	Salicylazosulfapyridine	Triamterene	542
Structure	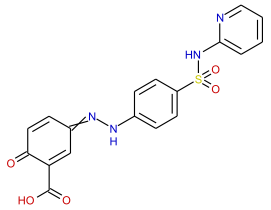	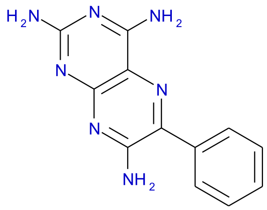	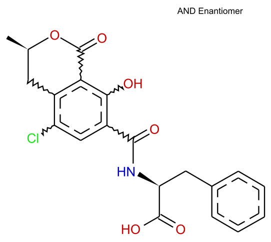
Actual Endpoint (-log C)	2.5034	3.62397	4.79932
Predicted Endpoint (-log C)	3.54214	4.35116	3.6353
Distance	0.795	0.797	0.812
Reference	CPDB	CPDB	CPDB

Model Applicability

Unknown features are fingerprint features in the query molecule, but not found or appearing too infrequently in the training set.

1. All properties and OPS components are within expected ranges.
2. Unknown ECFP_2 feature: 1796421070: [*]OC(=C([*])[*])[c](:[*]):[*]
3. Unknown ECFP_2 feature: 1793888374: [*]C(=C(O)C(=[*])[*])[*]

Feature Contribution

Top features for positive contribution

Fingerprint	Bit/Smiles	Feature Structure	Score
ECFP_6	683445015	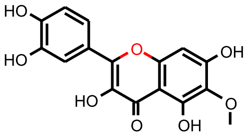 [*]O[*]	0.136

ECFP_6	-181568884	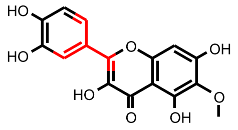 [*]C(=[*])[c](:[cH]:[*]):[cH]:[*]	0.0725
ECFP_6	734603939	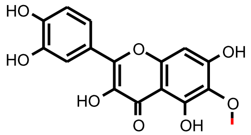 [*]C	0.0424

Top Features for negative contribution

Fingerprint	Bit/Smiles	Feature Structure	Score
ECFP_6	2106656448	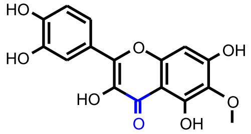 [*]C(=O)[*]	-0.275
ECFP_6	2019062761	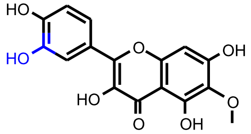 [*]:[c](:[*])O	-0.258
ECFP_6	1996767644	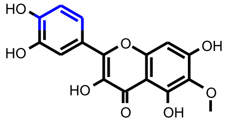 [*][c](:[*]):[cH]:[cH]:[*]	-0.251

Remdesivir

TOPKAT_Carcinogenic_Potency_TD50_Mouse

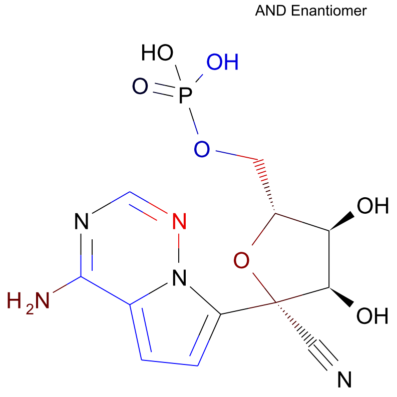

$C_{12}H_{14}N_5O_7P$
Molecular Weight: 371.24262
ALogP: -1.502
Rotatable Bonds: 4
Acceptors: 11
Donors: 5

Model Prediction

Prediction: 9.25
Unit: mg/kg_body_weight/day
Mahalanobis Distance: 14
Mahalanobis Distance p-value: 2.59e-010

Mahalanobis Distance: The Mahalanobis distance (MD) is a generalization of the Euclidean distance that accounts for correlations among the X properties. It is calculated as the distance to the center of the training data. The larger the MD, the less trustworthy the prediction.
Mahalanobis Distance p-value: The p-value gives the fraction of training data with an MD greater than or equal to the one for the given sample, assuming normally distributed data. The smaller the p-value, the less trustworthy the prediciton. For highly non-normal X properties (e.g., fingerprints), the MD p-value is wildly inaccurate.

Structural Similar Compounds

Name	377	(N-6)-(Methylnitroso)adenosine	338
Structure	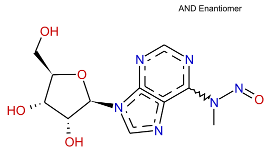	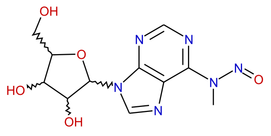	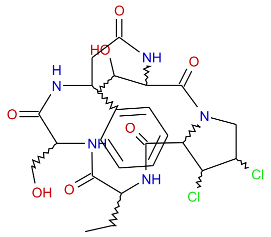
Actual Endpoint (-log C)	4.22928	4.22928	4.39533
Predicted Endpoint (-log C)	5.36013	5.36013	4.31268
Distance	0.852	0.852	0.919
Reference	CPDB	CPDB	CPDB

Model Applicability

Unknown features are fingerprint features in the query molecule, but not found or appearing too infrequently in the training set.

1. All properties and OPS components are within expected ranges.
2. Unknown ECFP_2 feature: 1126642748: [*]OP(=O)(O)O
3. Unknown ECFP_2 feature: 2024329577: [*]P(=[*])([*])O
4. Unknown ECFP_2 feature: -194719409: [*][C@@H]1[*][*]C([*])([*])O1
5. Unknown ECFP_2 feature: 1258791451: [*][C@@H]1[*][*]O[C@]1(C#[*])[c](:[*]):[*]
6. Unknown ECFP_2 feature: -264833661: [*]C([*])([*])C#N
7. Unknown ECFP_2 feature: -1507082173: [*][c]1:[*]:[*]:[c](:[*]):n:1:n:[*]
8. Unknown ECFP_2 feature: -676555381: [*]:n(:[*]):n:c:[*]
9. Unknown ECFP_2 feature: -66263742: [*]C([*])([*])[c]1:n(:[*]):[*]:[*]:c:1

Feature Contribution

Top features for positive contribution

Fingerprint	Bit/Smiles	Feature Structure	Score

ECFP_6	655739385	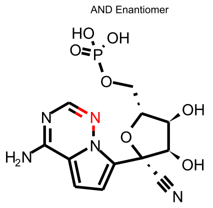 [*]:n:[*]	0.229
ECFP_6	1572579716	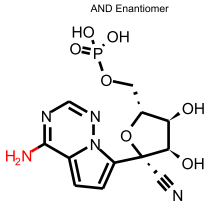 [*]N	0.225
ECFP_6	1559650422	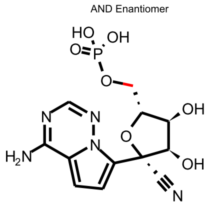 [*]C[*]	0.203

Top Features for negative contribution

**Fingerprint**	**Bit/Smiles**	**Feature Structure**	**Score**
ECFP_6	1996767644	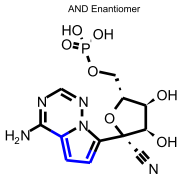 [*][c](:[*]):[cH]:[cH]:[*]	-0.251
ECFP_6	642810091	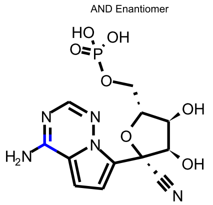 [*][c](:[*]):[*]	-0.247

ECFP_6	-182236392	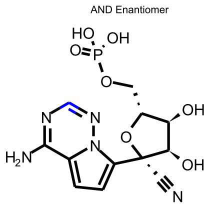 [*]:[cH]:[*]	-0.232

Patuletin

TOPKAT_Carcinogenic_Potency_TD50_Rat

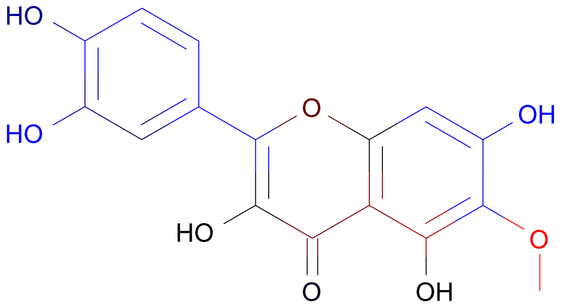

$C_{16}H_{12}O_8$
Molecular Weight: 332.26168
ALogP: 1.614
Rotatable Bonds: 2
Acceptors: 8
Donors: 5

Model Prediction

Prediction: 7.46
Unit: mg/kg_body_weight/day
Mahalanobis Distance: 10.8
Mahalanobis Distance p-value: 0.0373

Mahalanobis Distance: The Mahalanobis distance (MD) is a generalization of the Euclidean distance that accounts for correlations among the X properties. It is calculated as the distance to the center of the training data. The larger the MD, the less trustworthy the prediction.
Mahalanobis Distance p-value: The p-value gives the fraction of training data with an MD greater than or equal to the one for the given sample, assuming normally distributed data. The smaller the p-value, the less trustworthy the prediciton. For highly non-normal X properties (e.g., fingerprints), the MD p-value is wildly inaccurate.

Structural Similar Compounds

Name	Quercetin	Hematoxylin	454
Structure	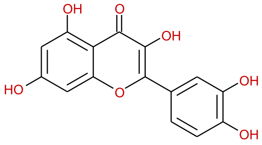	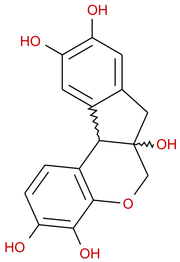	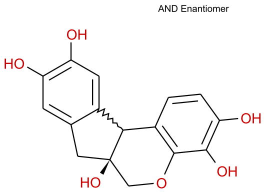
Actual Endpoint (-log C)	4.47602	2.48041	2.48041
Predicted Endpoint (-log C)	3.79194	4.42178	4.42178
Distance	0.258	0.616	0.616
Reference	CPDB	CPDB	CPDB

Model Applicability

Unknown features are fingerprint features in the query molecule, but not found or appearing too infrequently in the training set.

1. All properties and OPS components are within expected ranges.

Feature Contribution

Top features for positive contribution			
**Fingerprint**	**Bit/Smiles**	**Feature Structure**	**Score**
FCFP_6	136627117	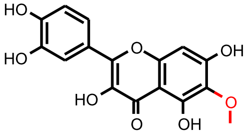 [*]OC	0.69

FCFP_6	1	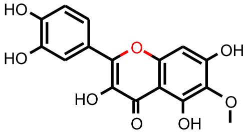 [*]O[*]	0.234
FCFP_6	203677720	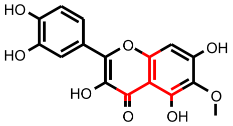 [*]C(=[*])[c](:[c]([*]):[*]):[c]([*]):[*]	0.137

Top Features for negative contribution

Fingerprint	Bit/Smiles	Feature Structure	Score
FCFP_6	7	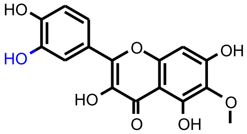 [*]O	-0.372
FCFP_6	16	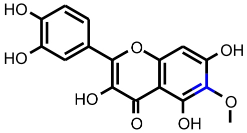 [*][c](:[*]):[*]	-0.354
FCFP_6	74595001	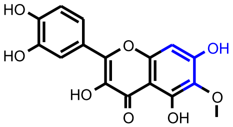 [*][c](:[*]):[c](O):[cH]:[*]	-0.267

Remdesivir

TOPKAT_Carcinogenic_Potency_TD50_Rat

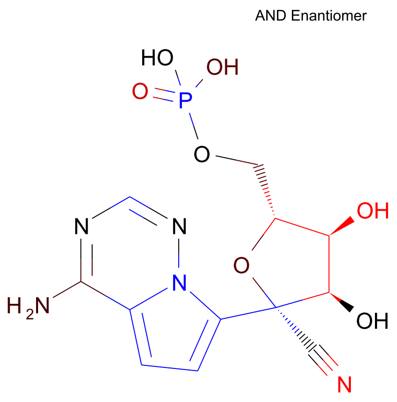

$C_{12}H_{14}N_5O_7P$

Molecular Weight: 371.24262

ALogP: -1.502

Rotatable Bonds: 4

Acceptors: 11

Donors: 5

Model Prediction

Prediction: 1.01

Unit: mg/kg_body_weight/day

Mahalanobis Distance: 16.2

Mahalanobis Distance p-value: 4.38e-015

Mahalanobis Distance: The Mahalanobis distance (MD) is a generalization of the Euclidean distance that accounts for correlations among the X properties. It is calculated as the distance to the center of the training data. The larger the MD, the less trustworthy the prediction.
Mahalanobis Distance p-value: The p-value gives the fraction of training data with an MD greater than or equal to the one for the given sample, assuming normally distributed data. The smaller the p-value, the less trustworthy the prediciton. For highly non-normal X properties (e.g., fingerprints), the MD p-value is wildly inaccurate.

Structural Similar Compounds

Name	b-Thioguanine deoxyriboside	Hexamethylmelamine	604
Structure	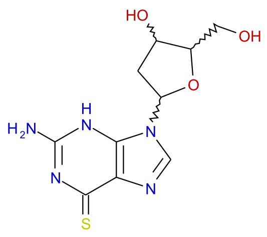	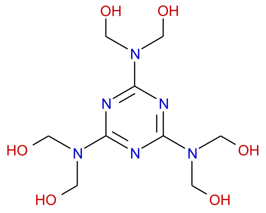	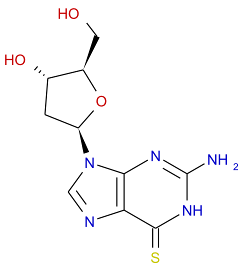
Actual Endpoint (-log C)	5.13004	4.47751	5.13004
Predicted Endpoint (-log C)	4.82552	3.76275	4.96687
Distance	0.805	0.832	0.835
Reference	CPDB	CPDB	CPDB

Model Applicability

Unknown features are fingerprint features in the query molecule, but not found or appearing too infrequently in the training set.

1. All properties and OPS components are within expected ranges.
2. Unknown FCFP_2 feature: 472180098: [*]OP(=O)(O)O
3. Unknown FCFP_2 feature: -836603894: [*][C@@H]1[*][*]O[C@]1(C#[*])[c](:[*]):[*]

Feature Contribution

Top features for positive contribution

Fingerprint	Bit/Smiles	Feature Structure	Score
FCFP_6	-1043250487	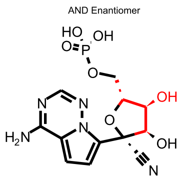 [*][C@H]1[*][*][C@H]([*])[C@H]1O	1.15

FCFP_6	9	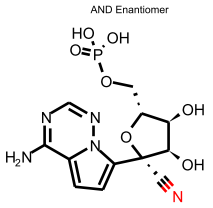 [*]#N	0.385
FCFP_6	1	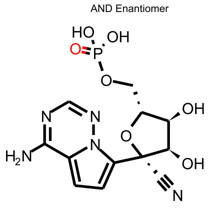 [*]O[*]	0.234

Top Features for negative contribution

Fingerprint	Bit/Smiles	Feature Structure	Score
FCFP_6	-1280036918	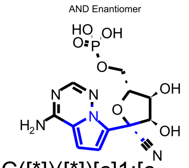 [*]C([*])([*])[c]1:[cH]:[cH]:[c](:[*]):n:1:[*]	-0.363
FCFP_6	16	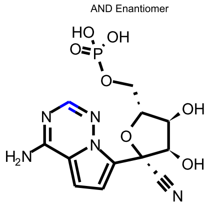 [*][c](:[*]):[*]	-0.354
FCFP_6	17	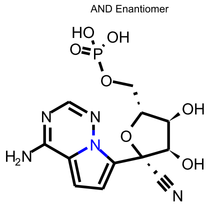 [*]:n(:[*]):[*]	-0.149

Patuletin

TOPKAT_Chronic_LOAEL

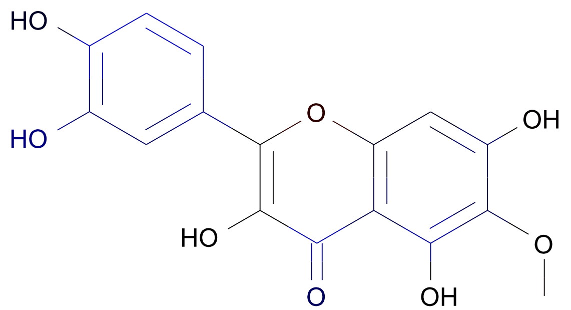

$C_{16}H_{12}O_8$
Molecular Weight: 332.26168
ALogP: 1.614
Rotatable Bonds: 2
Acceptors: 8
Donors: 5

Model Prediction

Prediction: 0.189
Unit: g/kg_body_weight
Mahalanobis Distance: 19.2
Mahalanobis Distance p-value: 5.07e-006

Mahalanobis Distance: The Mahalanobis distance (MD) is a generalization of the Euclidean distance that accounts for correlations among the X properties. It is calculated as the distance to the center of the training data. The larger the MD, the less trustworthy the prediction.
Mahalanobis Distance p-value: The p-value gives the fraction of training data with an MD greater than or equal to the one for the given sample, assuming normally distributed data. The smaller the p-value, the less trustworthy the prediciton. For highly non-normal X properties (e.g., fingerprints), the MD p-value is wildly inaccurate.

Structural Similar Compounds

Name	QUERCETIN	OLSALAZINE.NA	C.I. DISPERSE BLUE I
Structure	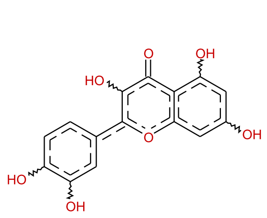	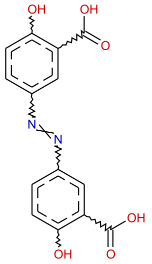	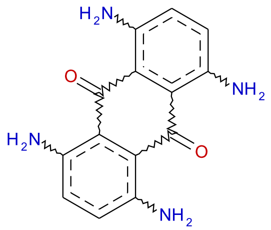
Actual Endpoint (-log C)	2.87829	3.17932	3.6327
Predicted Endpoint (-log C)	3.12498	2.89417	3.26657
Distance	0.255	0.577	0.707
Reference	NTP 409 79	NDA-19715	NTP REPORT # 299

Model Applicability

Unknown features are fingerprint features in the query molecule, but not found or appearing too infrequently in the training set.

1. All properties and OPS components are within expected ranges.
2. Unknown ECFP_6 feature: -1531301414: [*]O[c](:[c]([*]):[*]):[c]([*]):[*]
3. Unknown ECFP_6 feature: -570915357: [*]O[c](:[cH]:[*]):[c]([*]):[*]
4. Unknown ECFP_6 feature: -813997308: [*]C(=[*])[c](:[c]([*]):[*]):[c]([*]):[*]
5. Unknown ECFP_6 feature: -1660913849: [*][c](:[*]):[c](O):[c]([*]):[*]
6. Unknown ECFP_6 feature: -560785749: [*]C(=[*])O[c](:[*]):[*]
7. Unknown ECFP_6 feature: 1796421070: [*]OC(=C([*])[*])[c](:[*]):[*]
8. Unknown ECFP_6 feature: 1793888374: [*]C(=C(O)C(=[*])[*])[*]
9. Unknown ECFP_6 feature: 1717462980: [*]C(=[*])C(=O)[c](:[*]):[*]
10. Unknown ECFP_6 feature: -181568884: [*]C(=[*])[c](:[cH]:[*]):[cH]:[*]
11. Unknown ECFP_6 feature: 2019062761: [*]:[c](:[*])O
12. Unknown ECFP_6 feature: 1307307440: [*]:[c](:[*])OC

Feature Contribution

Top features for positive contribution			
**Fingerprint**	**Bit/Smiles**	**Feature Structure**	**Score**

ECFP_6	683445015	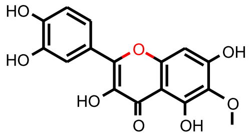 [*]O[*]	0.0734
FCFP_6	1036089772	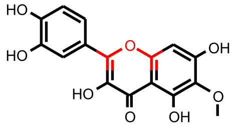 [*]C(=[*])O[c](:[*]):[*]	0.073
FCFP_6	136627117	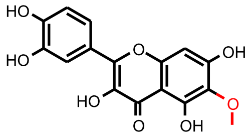 [*]OC	0.0538
**Top Features for negative contribution**			
**Fingerprint**	**Bit/Smiles**	**Feature Structure**	**Score**
ECFP_6	2106656448	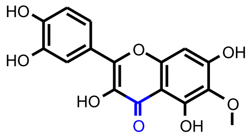 [*]C(=O)[*]	-0.11
FCFP_6	1	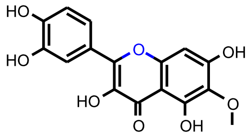 [*]O[*]	-0.102

FCFP_6	-453677277	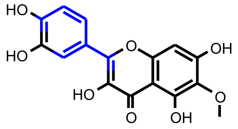<br>[*]C(=[*])[c]1:[cH]:[*]:[c]([*]):[cH]:[cH]:1	-0.0906

AND Enantiomer

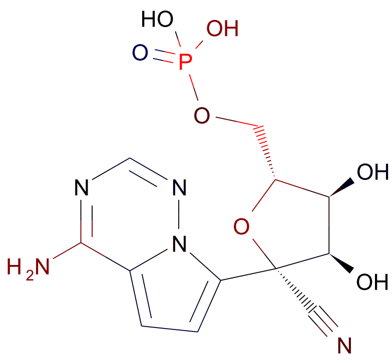

$C_{12}H_{14}N_5O_7P$
Molecular Weight: 371.24262
ALogP: -1.502
Rotatable Bonds: 4
Acceptors: 11
Donors: 5

Model Prediction

Prediction: 0.00379
Unit: g/kg_body_weight
Mahalanobis Distance: 47.7
Mahalanobis Distance p-value: 2.93e-054

Mahalanobis Distance: The Mahalanobis distance (MD) is a generalization of the Euclidean distance that accounts for correlations among the X properties. It is calculated as the distance to the center of the training data. The larger the MD, the less trustworthy the prediction.
Mahalanobis Distance p-value: The p-value gives the fraction of training data with an MD greater than or equal to the one for the given sample, assuming normally distributed data. The smaller the p-value, the less trustworthy the prediciton. For highly non-normal X properties (e.g., fingerprints), the MD p-value is wildly inaccurate.

Structural Similar Compounds

Name	TETRACYCLINE .HCL	4;4'-DIAMINO-2;2'-STILBENEDIS	OXYTETRACYCLINE .HCL
Structure	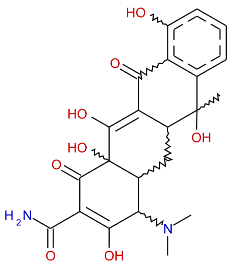	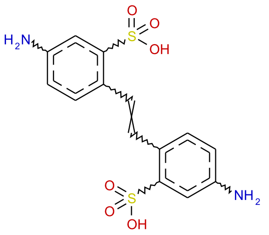	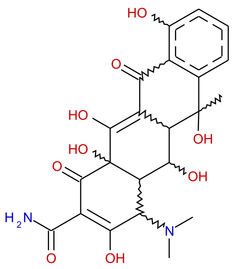
Actual Endpoint (-log C)	2.85193	2.47175	2.56626
Predicted Endpoint (-log C)	3.94748	3.53715	3.75581
Distance	0.746	0.746	0.802
Reference	NTP REPORT # 344	NTP 412 82	NTP REPORT # 315

Model Applicability

Unknown features are fingerprint features in the query molecule, but not found or appearing too infrequently in the training set.

1. All properties and OPS components are within expected ranges.
2. Unknown FCFP_2 feature: 472180098: [*]OP(=O)(O)O
3. Unknown FCFP_2 feature: -332197802: [*][c]1:[*]:[*]:[c](:[*]):n:1:n:[*]
4. Unknown ECFP_6 feature: -1114776580: [*]C#[*]
5. Unknown ECFP_6 feature: -1101847286: [*]#N
6. Unknown ECFP_6 feature: 672362763: [*]:n(:[*]):[*]
7. Unknown ECFP_6 feature: 1126642748: [*]OP(=O)(O)O
8. Unknown ECFP_6 feature: 2100964382: [*]P(=O)([*])[*]
9. Unknown ECFP_6 feature: 2024329577: [*]P(=[*])([*])O
10. Unknown ECFP_6 feature: -1250439909: [*]COP(=[*])([*])[*]
11. Unknown ECFP_6 feature: -1687549011: [*]OCC([*])[*]
12. Unknown ECFP_6 feature: -194719409: [*][C@@H]1[*][*]C([*])([*])O1
13. Unknown ECFP_6 feature: -553149446: [*]C[C@H]1O[*][*][C@@H]1[*]
14. Unknown ECFP_6 feature: 305695353: [*][C@H]1[*][*][C@H]([*])[C@H]1O
15. Unknown ECFP_6 feature: -521596699: [*][C@@H]1[*][*]C([*])([*])[C@@H]1O
16. Unknown ECFP_6 feature: 1258791451: [*][C@@H]1[*][*]O[C@]1(C#[*])[c](:[*]):[*]
17. Unknown ECFP_6 feature: 2024749573: [*]C([*])O
18. Unknown ECFP_6 feature: -264833661: [*]C([*])([*])C#N
19. Unknown ECFP_6 feature: 1412053881: [*]C#N

20. Unknown ECFP_6 feature: -1507082173: [*][c]1:[*]:[*]:[c](:[*]):n:1:n:[*]
21. Unknown ECFP_6 feature: -676555381: [*]:[cH]:n:n(:[*]):[*]
22. Unknown ECFP_6 feature: -710237522: [*]:n:[cH]:n:[*]
23. Unknown ECFP_6 feature: -677309799: [*][c](:[*]):n:[cH]:[*]
24. Unknown ECFP_6 feature: -1734834311: [*]:n:[c](N):[c](:[*]):[*]
25. Unknown ECFP_6 feature: 1334415134: [*][c](:[*]):[c]1:[cH]:[*]:[*]:n:1:[*]
26. Unknown ECFP_6 feature: -66263742: [*]C([*])([*])[c]1:[cH]:[*]:[*]:n:1:[*]
27. Unknown ECFP_6 feature: -938530932: [*]:[c](:[*])N

Feature Contribution

Top features for positive contribution

Fingerprint	Bit/Smiles	Feature Structure	Score
ECFP_6	-167460056	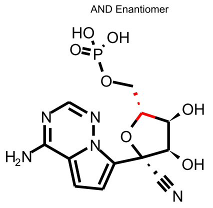 [*]C([*])[*]	0.136
FCFP_6	-1143715940	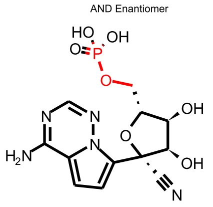 [*]COP(=[*])([*])[*]	0.13
ECFP_6	1559650422	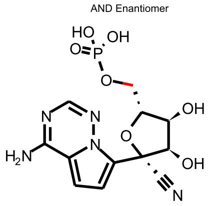 [*]C[*]	0.129

Top Features for negative contribution

Fingerprint	Bit/Smiles	Feature Structure	Score

FCFP_6	1	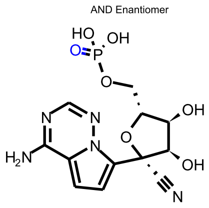 [*]O[*]	-0.102
ECFP_6	1996767644	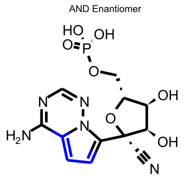 [*][c](:[*]):[cH]:[cH]:[*]	-0.0497
FCFP_6	16	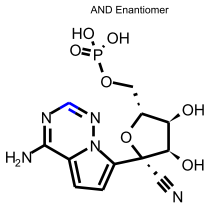 [*][c](:[*]):[*]	-0.0462

Patuletin

TOPKAT_Rat_Maximum_Tolerated_Dose_Feed

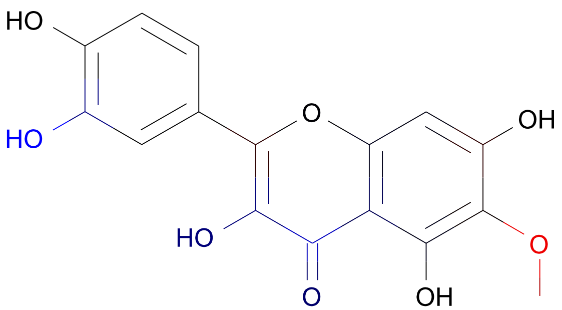

$C_{16}H_{12}O_8$

Molecular Weight: 332.26168

ALogP: 1.614

Rotatable Bonds: 2

Acceptors: 8

Donors: 5

Model Prediction

Prediction: 1.06

Unit: g/kg_body_weight

Mahalanobis Distance: 8.52

Mahalanobis Distance p-value: 0.00606

Mahalanobis Distance: The Mahalanobis distance (MD) is a generalization of the Euclidean distance that accounts for correlations among the X properties. It is calculated as the distance to the center of the training data. The larger the MD, the less trustworthy the prediction.

Mahalanobis Distance p-value: The p-value gives the fraction of training data with an MD greater than or equal to the one for the given sample, assuming normally distributed data. The smaller the p-value, the less trustworthy the prediciton. For highly non-normal X properties (e.g., fingerprints), the MD p-value is wildly inaccurate.

Structural Similar Compounds

Name	QUERCETIN	50%1,4,5,8-TETRAAMINOANTHRAQUIONONE + DERIVATIVES	4,4'-DIAMINO-2,2'-STILBENEDISULFONIC ACID.2NaSALT
Structure	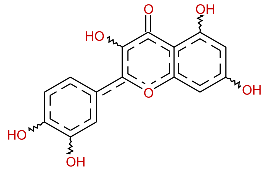	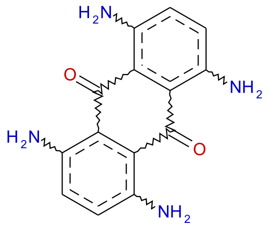	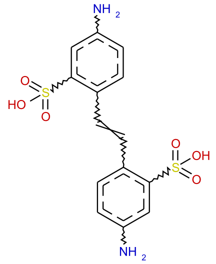
Actual Endpoint (-log C)	2.2016	3.0764	2.50759
Predicted Endpoint (-log C)	2.27782	3.08142	3.26068
Distance	0.149	0.686	0.731
Reference	NCI/NTP TR-409	NCI/NTP TR-299	NCI/NTP TR-412

Model Applicability

Unknown features are fingerprint features in the query molecule, but not found or appearing too infrequently in the training set.

1. All properties and OPS components are within expected ranges.

Feature Contribution

Top features for positive contribution

Fingerprint	Bit/Smiles	Feature Structure	Score
FCFP_2	136627117	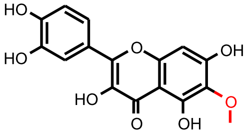 [*]OC	0.173

FCFP_2	1036089772	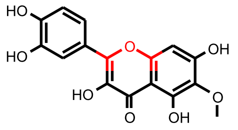 [*]C(=[*])O[c](:[*]):[*]	0.0749
FCFP_2	332760439	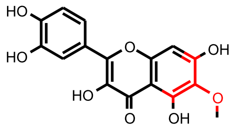 [*]O[c](:[c]([*]):[*]):[c]([*]):[*]	0.0611

Top Features for negative contribution

Fingerprint	Bit/Smiles	Feature Structure	Score
FCFP_2	7	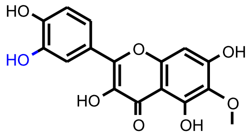 [*]O	-0.214
FCFP_2	-549108873	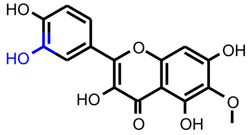 [*]:[c](:[*])O	-0.127
FCFP_2	-548632217	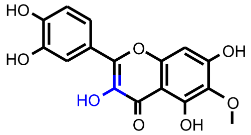 [*]C(=[*])O	-0.119

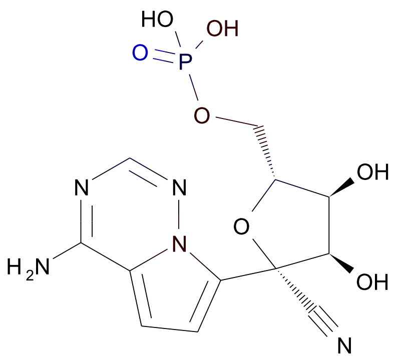

$C_{12}H_{14}N_5O_7P$
Molecular Weight: 371.24262
ALogP: -1.502
Rotatable Bonds: 4
Acceptors: 11
Donors: 5

Model Prediction

Prediction: 0.235
Unit: g/kg_body_weight
Mahalanobis Distance: 9.52
Mahalanobis Distance p-value: 0.000247

Mahalanobis Distance: The Mahalanobis distance (MD) is a generalization of the Euclidean distance that accounts for correlations among the X properties. It is calculated as the distance to the center of the training data. The larger the MD, the less trustworthy the prediction.
Mahalanobis Distance p-value: The p-value gives the fraction of training data with an MD greater than or equal to the one for the given sample, assuming normally distributed data. The smaller the p-value, the less trustworthy the prediciton. For highly non-normal X properties (e.g., fingerprints), the MD p-value is wildly inaccurate.

Structural Similar Compounds

Name	4,4'-DIAMINO-2,2'-STILBENEDISULFONIC ACID.2NaSALT	OXYTETRACYCLINE	50%1,4,5,8-TETRAAMINOANTHRAQUIONONE + DERIVATIVES
Structure	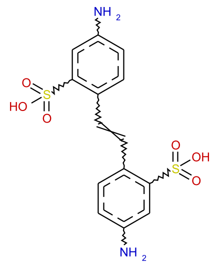	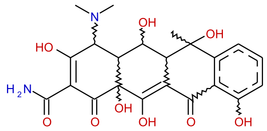	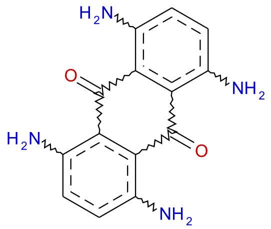
Actual Endpoint (-log C)	2.50759	2.36214	3.0764
Predicted Endpoint (-log C)	3.26068	2.77834	3.08142
Distance	0.743	0.818	0.989
Reference	NCI/NTP TR-412	NCI/NTP TR-315	NCI/NTP TR-299

Model Applicability

Unknown features are fingerprint features in the query molecule, but not found or appearing too infrequently in the training set.

1. Molecular_PolarSurfaceArea out of range. Value: 206.26. Training min, max, mean, SD: 0, 201.84, 63.052, 40.7.
2. Unknown FCFP_2 feature: 472180098: [*]OP(=O)(O)O
3. Unknown FCFP_2 feature: -836603894: [*][C@@H]1[*][*]O[C@]1(C#[*])[c](:[*]):[*]
4. Unknown FCFP_2 feature: -1277879912: [*]C([*])([*])C#N
5. Unknown FCFP_2 feature: -332197802: [*][c]1:[*]:[*]:[c](:[*]):n:1:n:[*]
6. Unknown FCFP_2 feature: -124685461: [*]:n:c:n:[*]

Feature Contribution

Top features for positive contribution			
Fingerprint	Bit/Smiles	Feature Structure	Score

FCFP_2	-1143715940	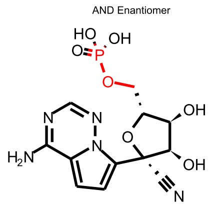 [*]COP(=[*])([*])[*]	0.095
FCFP_2	3	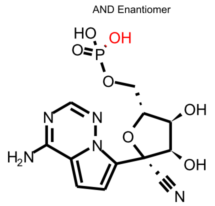 [*]O	0.0737
FCFP_2	17	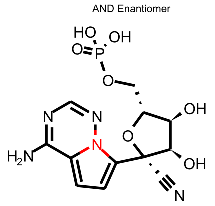 [*]:n(:[*]):[*]	0.0441
**Top Features for negative contribution**			
**Fingerprint**	**Bit/Smiles**	**Feature Structure**	**Score**
FCFP_2	1872154524	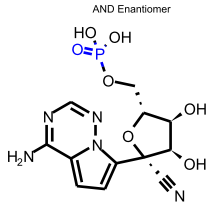 [*]C(=O)[*]	-0.105
FCFP_2	1	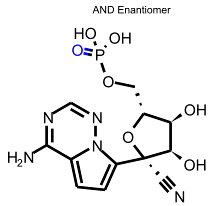 [*]O[*]	-0.0796

FCFP_2	16	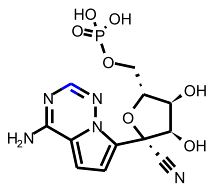 [*][c](:[*]):[*]	-0.0512

Patuletin

TOPKAT_Rat_Maximum_Tolerated_Dose_Gavage

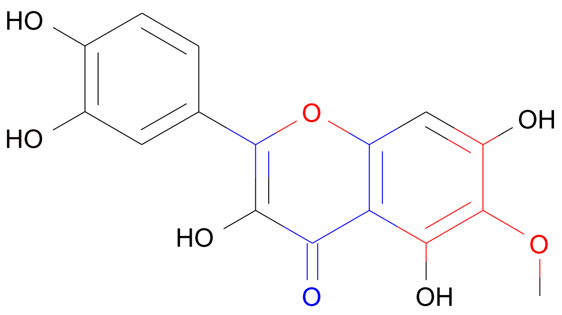

$C_{16}H_{12}O_8$
Molecular Weight: 332.26168
ALogP: 1.614
Rotatable Bonds: 2
Acceptors: 8
Donors: 5

Model Prediction

Prediction: 0.000111
Unit: g/kg_body_weight
Mahalanobis Distance: 17.3
Mahalanobis Distance p-value: 4.62e-016

Mahalanobis Distance: The Mahalanobis distance (MD) is a generalization of the Euclidean distance that accounts for correlations among the X properties. It is calculated as the distance to the center of the training data. The larger the MD, the less trustworthy the prediction.
Mahalanobis Distance p-value: The p-value gives the fraction of training data with an MD greater than or equal to the one for the given sample, assuming normally distributed data. The smaller the p-value, the less trustworthy the prediciton. For highly non-normal X properties (e.g., fingerprints), the MD p-value is wildly inaccurate.

Structural Similar Compounds

Name	OCHRATOXIN	AMPICILLIN TRIHYDRATE	HC RED 3
Structure	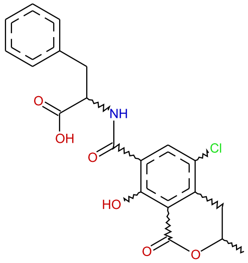	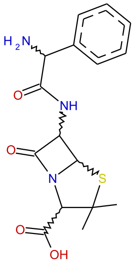	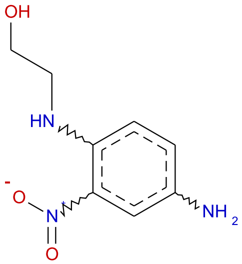
Actual Endpoint (-log C)	6.28396	2.36724	2.59592
Predicted Endpoint (-log C)	5.12358	2.27651	3.285
Distance	0.926	1.070	1.091
Reference	NCI/NTP TR-358	NCI/NTP TR-318	NCI/NTP TR-281

Model Applicability

Unknown features are fingerprint features in the query molecule, but not found or appearing too infrequently in the training set.

1. Num_H_Donors out of range. Value: 5. Training min, max, mean, SD: 0, 3, 0.4375, 0.8311.
2. Num_H_Acceptors out of range. Value: 8. Training min, max, mean, SD: 0, 6, 1.6146, 1.644.
3. Molecular_PolarSASA out of range. Value: 233.06. Training min, max, mean, SD: 0, 223.97, 50.816, 55.15.
4. OPS PC1 out of range. Value: 8.81. Training min, max, SD, explained variance: -4.0008, 7.9165, 2.861, 0.2531.
5. OPS PC9 out of range. Value: 3.4228. Training min, max, SD, explained variance: -2.7086, 2.9267, 1.019, 0.0321.
6. Unknown FCFP_2 feature: -1678245750: [*]OC(=C([*])[*])[c](:[*]):[*]
7. Unknown FCFP_2 feature: -1305924292: [*]C(=C(O)C(=[*])[*])[*]
8. Unknown FCFP_2 feature: -1549192822: [*]C(=[*])C(=O)[c](:[*]):[*]

Feature Contribution

Top features for positive contribution			
Fingerprint	Bit/Smiles	Feature Structure	Score

FCFP_2	332760439	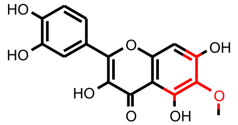 [*]O[c](:[c]([*]):[*]<br>):[c]([*]):[*]	0.672
FCFP_2	1	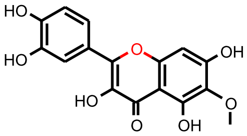 [*]O[*]	0.511
FCFP_2	136627117	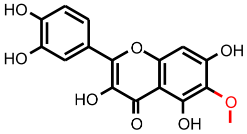 [*]OC	0.0304
**Top Features for negative contribution**			
**Fingerprint**	**Bit/Smiles**	**Feature Structure**	**Score**
FCFP_2	203677720	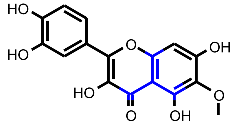 [*]C(=[*])[c](:[c]([*<br>]):[*]):[c]([*]):[*]	-0.406
FCFP_2	1872154524	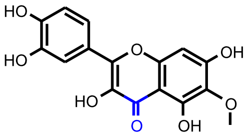 [*]C(=O)[*]	-0.307

FCFP_2	0	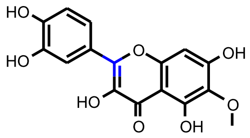<br>[*]C(=[*])[*]	-0.29

Remdesivir

TOPKAT_Rat_Maximum_Tolerated_Dose_Gavage

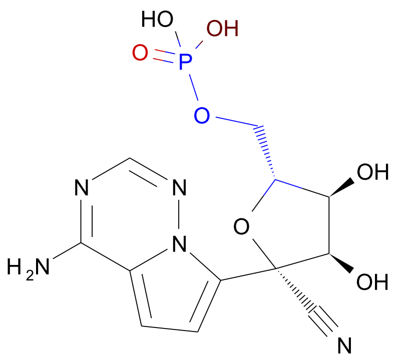

$C_{12}H_{14}N_5O_7P$
Molecular Weight: 371.24262
ALogP: -1.502
Rotatable Bonds: 4
Acceptors: 11
Donors: 5

Model Prediction

Prediction: 0.000298
Unit: g/kg_body_weight
Mahalanobis Distance: 17.2
Mahalanobis Distance p-value: 5.05e-016

Mahalanobis Distance: The Mahalanobis distance (MD) is a generalization of the Euclidean distance that accounts for correlations among the X properties. It is calculated as the distance to the center of the training data. The larger the MD, the less trustworthy the prediction.
Mahalanobis Distance p-value: The p-value gives the fraction of training data with an MD greater than or equal to the one for the given sample, assuming normally distributed data. The smaller the p-value, the less trustworthy the prediciton. For highly non-normal X properties (e.g., fingerprints), the MD p-value is wildly inaccurate.

Structural Similar Compounds

Name	AMPICILLIN TRIHYDRATE	OCHRATOXIN	PENICILLIN VK
Structure	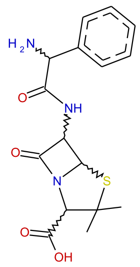	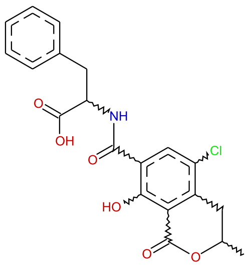	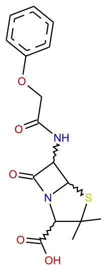
Actual Endpoint (-log C)	2.36724	6.28396	2.54455
Predicted Endpoint (-log C)	2.27651	5.12358	3.9702
Distance	1.255	1.482	1.498
Reference	NCI/NTP TR-318	NCI/NTP TR-358	NCI/NTP TR-336

Model Applicability

Unknown features are fingerprint features in the query molecule, but not found or appearing too infrequently in the training set.

1. Num_H_Donors out of range. Value: 5. Training min, max, mean, SD: 0, 3, 0.4375, 0.8311.
2. Num_H_Acceptors out of range. Value: 11. Training min, max, mean, SD: 0, 6, 1.6146, 1.644.
3. Molecular_PolarSASA out of range. Value: 321.97. Training min, max, mean, SD: 0, 223.97, 50.816, 55.15.
4. Molecular_PolarSurfaceArea out of range. Value: 206.26. Training min, max, mean, SD: 0, 138.03, 28.978, 32.1.
5. OPS PC1 out of range. Value: 9.0116. Training min, max, SD, explained variance: -4.0008, 7.9165, 2.861, 0.2531.
6. OPS PC5 out of range. Value: -4.1876. Training min, max, SD, explained variance: -3.4, 4.1587, 1.489, 0.0686.
7. OPS PC9 out of range. Value: -2.7276. Training min, max, SD, explained variance: -2.7086, 2.9267, 1.019, 0.0321.
8. Unknown FCFP_2 feature: 472180098: [*]OP(=O)(O)O
9. Unknown FCFP_2 feature: -836603894: [*][C@@H]1[*][*]O[C@]1(C#[*])[c](:[*]):[*]
10. Unknown FCFP_2 feature: -1277879912: [*]C([*])([*])C#N
11. Unknown FCFP_2 feature: -1362791977: [*]C#N
12. Unknown FCFP_2 feature: -332197802: [*][c]1:[*]:[*]:[c](:[*]):n:1:n:[*]
13. Unknown FCFP_2 feature: -124685461: [*]:n:c:n:[*]

Feature Contribution

Top features for positive contribution

Fingerprint	Bit/Smiles	Feature Structure	Score
FCFP_2	1	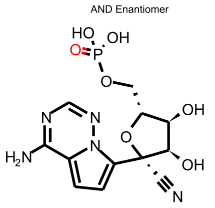 [*]O[*]	0.511
FCFP_2	3	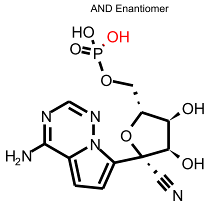 [*]O	0.104

Top Features for negative contribution

Fingerprint	Bit/Smiles	Feature Structure	Score
FCFP_2	1872154524	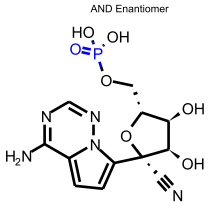 [*]C(=O)[*]	-0.307
FCFP_2	0	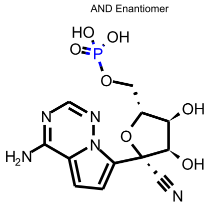 [*]C(=[*])[*]	-0.29

FCFP_2	-1272768868	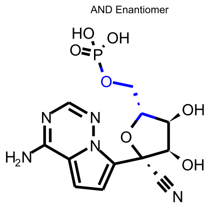[*]OCC([*])[*]	-0.271

Patuletin

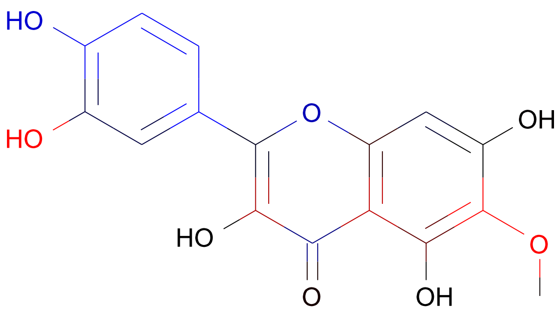

$C_{16}H_{12}O_8$
Molecular Weight: 332.26168
ALogP: 1.614
Rotatable Bonds: 2
Acceptors: 8
Donors: 5

Structural Similar Compounds

Name	QUERCETIN	ANTHRAQUINONE; 1;5-DIAMINO-4;8-DIHYDROXY-3-(p-METHOXYPHENYL)-	7H-PYRROLO[2;3-d]PYRIMIDIN-4-OL; 7-.beta.-d-RIBOFURANOSYL-
Structure	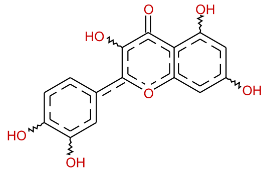	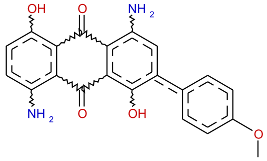	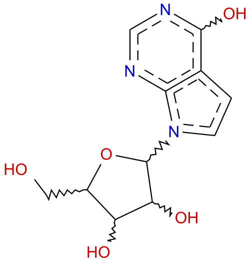
Actual Endpoint (-log C)	3.274	1.771	4.012
Predicted Endpoint (-log C)	2.40427	2.1122	3.00148
Distance	0.274	0.657	0.751
Reference	PSEBAA 77;269;51	28ZPAK -;245;72	CNREA8 29;116;69

Model Prediction

Prediction: 0.902
Unit: g/kg_body_weight
Mahalanobis Distance: 17.8
Mahalanobis Distance p-value: 0.00152

Mahalanobis Distance: The Mahalanobis distance (MD) is a generalization of the Euclidean distance that accounts for correlations among the X properties. It is calculated as the distance to the center of the training data. The larger the MD, the less trustworthy the prediction.
Mahalanobis Distance p-value: The p-value gives the fraction of training data with an MD greater than or equal to the one for the given sample, assuming normally distributed data. The smaller the p-value, the less trustworthy the prediciton. For highly non-normal X properties (e.g., fingerprints), the MD p-value is wildly inaccurate.

Model Applicability

Unknown features are fingerprint features in the query molecule, but not found or appearing too infrequently in the training set.

1. All properties and OPS components are within expected ranges.
2. Unknown FCFP_6 feature: 16: [*][c](:[*]):[*]
3. Unknown FCFP_6 feature: 74595001: [*][c](:[*]):[c](O):[cH]:[*]
4. Unknown FCFP_6 feature: 1618154665: [*][c](:[*]):[cH]:[c]([*]):[*]
5. Unknown FCFP_6 feature: -1678245750: [*]OC(=C([*])[*])[c](:[*]):[*]
6. Unknown FCFP_6 feature: -1305924292: [*]C(=C(O)C(=[*])[*])[*]
7. Unknown FCFP_6 feature: -549108873: [*]:[c](:[*])O

Feature Contribution

Top features for positive contribution			
Fingerprint	Bit/Smiles	Feature Structure	Score

ECFP_6	642810091	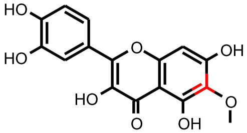 [*][c](:[*]):[*]	0.281
ECFP_6	-560785749	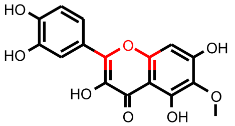 [*]C(=[*])O[c](:[*]):[*]	0.259
FCFP_6	136627117	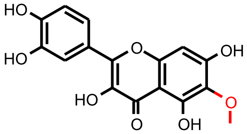 [*]OC	0.17

Top Features for negative contribution

Fingerprint	Bit/Smiles	Feature Structure	Score
ECFP_6	2106656448	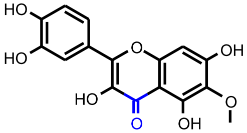 [*]C(=O)[*]	-0.352
ECFP_6	683445015	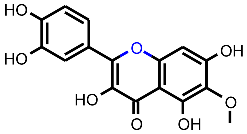 [*]O[*]	-0.266

FCFP_6	946589555	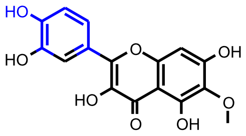 [*][c]1:[*]:[c]([*]):[c](O):[cH]:[cH]:1	-0.204

Remdesivir

TOPKAT_Rat_Oral_LD50

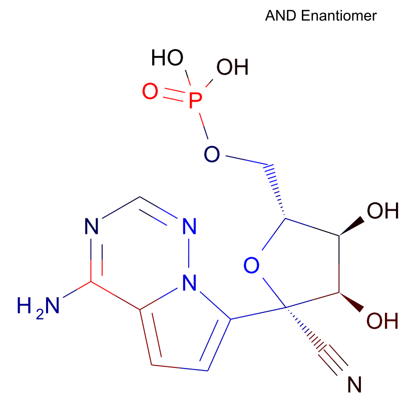

$C_{12}H_{14}N_5O_7P$

Molecular Weight: 371.24262

ALogP: -1.502

Rotatable Bonds: 4

Acceptors: 11

Donors: 5

Model Prediction

Prediction: 0.309

Unit: g/kg_body_weight

Mahalanobis Distance: 29.4

Mahalanobis Distance p-value: 1.72e-059

Mahalanobis Distance: The Mahalanobis distance (MD) is a generalization of the Euclidean distance that accounts for correlations among the X properties. It is calculated as the distance to the center of the training data. The larger the MD, the less trustworthy the prediction.

Mahalanobis Distance p-value: The p-value gives the fraction of training data with an MD greater than or equal to the one for the given sample, assuming normally distributed data. The smaller the p-value, the less trustworthy the prediciton. For highly non-normal X properties (e.g., fingerprints), the MD p-value is wildly inaccurate.

Structural Similar Compounds

Name	5'-ADENYLIC ACID; POTASSIUM SALT (K STRIPPED)	INOSINATE; DISODIUM SALT (Na STRIPPED)	INOSINE-5'-PHOSPHORIC ACID
Structure	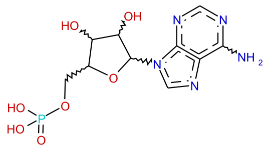	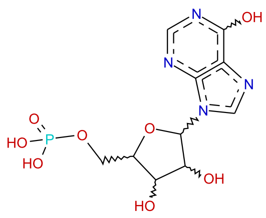	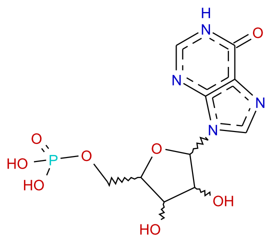
Actual Endpoint (-log C)	1.49	1.34	1.338
Predicted Endpoint (-log C)	2.45569	2.92201	1.35922
Distance	0.361	0.428	0.592
Reference	OYYAA2 4;689;70	AJINO* -;-;73	ARTODN 47;77;81

Model Applicability

Unknown features are fingerprint features in the query molecule, but not found or appearing too infrequently in the training set.

1. OPS PC10 out of range. Value: 15.526. Training min, max, SD, explained variance: -6.0395, 14.892, 2.468, 0.0220.
2. Unknown ECFP_2 feature: 1258791451: [*][C@@H]1[*][*]O[C@]1(C#[*])[c](:[*]):[*]
3. Unknown ECFP_2 feature: -264833661: [*]C([*])([*])C#N
4. Unknown ECFP_2 feature: -66263742: [*]C([*])([*])[c]1:n(:[*]):[*]:[*]:c:1
5. Unknown FCFP_6 feature: 16: [*][c](:[*]):[*]
6. Unknown FCFP_6 feature: 472180098: [*]OP(=O)(O)O
7. Unknown FCFP_6 feature: -836603894: [*][C@@H]1[*][*]O[C@]1(C#[*])[c](:[*]):[*]
8. Unknown FCFP_6 feature: -332197802: [*][c]1:[*]:[*]:[c](:[*]):n:1:n:[*]
9. Unknown FCFP_6 feature: 4427049: [*]:[cH]:n:n(:[*]):[*]
10. Unknown FCFP_6 feature: -124685461: [*]:n:[cH]:n:[*]
11. Unknown FCFP_6 feature: 1747237384: [*][c](:[*]):n:[cH]:[*]
12. Unknown FCFP_6 feature: -1151884458: [*]:n:[c](N):[c](:[*]):[*]
13. Unknown FCFP_6 feature: 1618154665: [*][c](:[*]):[cH]:[c]([*]):[*]
14. Unknown FCFP_6 feature: 1069584379: [*]:[c](:[*])N

Feature Contribution

Top features for positive contribution

Fingerprint	Bit/Smiles	Feature Structure	Score
ECFP_6	642810091	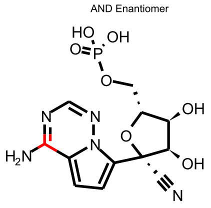 [*][c](:[*]):[*]	0.281
ECFP_6	-826638028	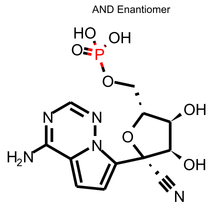 [*]P(=[*])([*])[*]	0.225
ECFP_6	2100964382	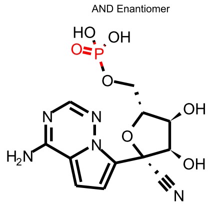 [*]P(=O)([*])[*]	0.166

Top Features for negative contribution

Fingerprint	Bit/Smiles	Feature Structure	Score
ECFP_6	683445015	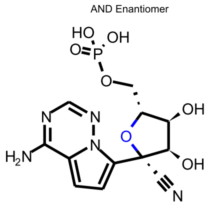 [*]O[*]	-0.266

ECFP_6	655739385	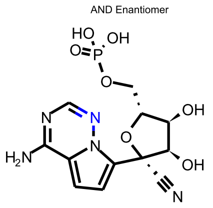<br>[*]:n:[*]	-0.239
FCFP_6	-1539132615	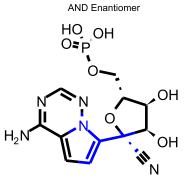<br>[*]C([*])([*])[c]1:[cH]:[*]:[*]:n:1:[*]	-0.2
